# Synthesis, Structure and Stereochemistry of Dispirocompounds Based on Imidazothiazolotriazine and Pyrrolidineoxindole

**DOI:** 10.3390/ijms232213820

**Published:** 2022-11-10

**Authors:** Alexei N. Izmest’ev, Valentina A. Karnoukhova, Alexander A. Larin, Angelina N. Kravchenko, Leonid L. Fershtat, Galina A. Gazieva

**Affiliations:** 1 N. D. Zelinsky Institute of Organic Chemistry, Russian Academy of Sciences, 47 Leninsky Prosp., Moscow 119991, Russia; 2 A. N. Nesmeyanov Institute of Organoelement Compounds, Russian Academy of Sciences, 28 Vavilova Street, Moscow 119991, Russia

**Keywords:** dispirooxindoles, 1,3-dipolar cycloaddition, oxindolylidene imidazothiazolotriazines, azomethine ylides, rearrangement

## Abstract

Methods for the synthesis of two types of isomeric dispirocompounds based on imidazothiazolotriazine and pyrrolidineoxindole, differing in the structure of imidazothiazolotriazine fragment, namely, linear dispiro[imidazo[4,5-*e*]thiazolo[3,2-*b*][1,2,4]triazine-6,3′-pyrrolidine- 4′,3″-indolines] and angular dispiro[imidazo[4,5-*e*]thiazolo[2,3-*c*][1,2,4]triazine-7,3′-pyrrolidine-4′,3″-indolines] were proposed. The first method relies on a 1,3-dipolar cycloaddition of azomethine ylides generated in situ from paraformaldehyde and N-alkylglycine derivatives to the corresponding oxindolylidene derivatives of imidazothiazolotriazine. The cycloaddition leads to a mixture of two diastereomers resulted from *anti*- and *syn*-approaches of azomethine ylide in approximately a 1:1 ratio, which were separated by column chromatography. Another method consists in rearrangement of linear dispiro[imidazo[4,5-*e*]thiazolo[3,2-*b*][1,2,4]triazine-6,3′-pyrrolidine-4′,3″-indolines] into hitherto unavailable angular dispiro[imidazo[4,5-*e*]thiazolo[2,3-*c*]-[1,2,4]triazine-7,3′-pyrrolidine-4′,3″-indolines] upon treatment with KOH. It was found that the *anti*-diastereomer of linear type underwent rearrangement into the isomeric angular *syn*-diastereomer, while the rearrangement of the linear *syn*-diastereomer gave the angular *anti*-diastereomer.

## 1. Introduction

**Figure 1:**
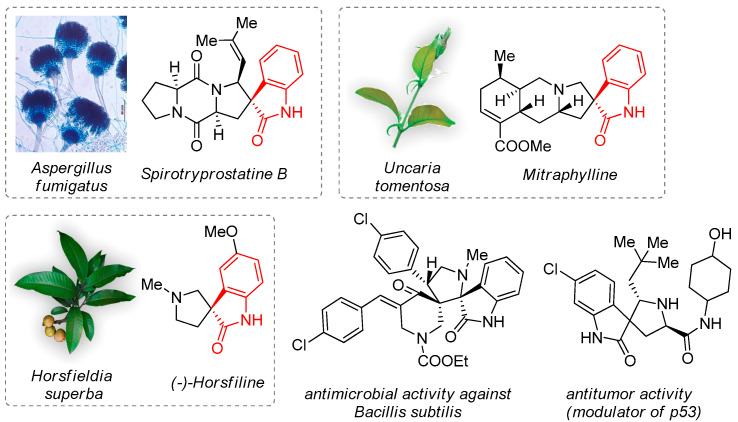
Biologically active natural and synthetic spiropyrrolidineoxindoles.

Spiro cyclic compounds have found great application in drug discovery due to the interesting conformational features and presence in natural products [1,2,3]. A conformational rigidity of the molecule allows spirocompounds to interact effectively with various active sites on many biological targets [3,4]. Therefore, the introduction of a spiro-junction in the structure affects the key properties of molecules, such as bioavailability, metabolic stability, binding ability, and target selectivity [5]. Synthetic and natural products containing a spiropyrrolidineoxindole fragment exhibit a wide scope of pharmacological activity, including antimicrobial, antitumor, antidiabetic, anticonvulsant, and antiviral effects (Figure 1) [6,7,8,9,10]. Promising biological properties encourage the researchers to develop efficient synthetic approaches to structures bearing such framework. There are numerous reports on the synthesis of various spiropyrrolidineoxindoles and dispiro-fused pyrrolidineoxindoles, additionally involving spiro-linked pharmacophore heterocycles, such as thiazolidine, imidazolidine, etc. [11,12,13,14,15]. Such hybridization resulted in a preparation of new compounds with cytotoxic, prooxidant and antidiabetic properties [15,16,17].

**Figure 2:**
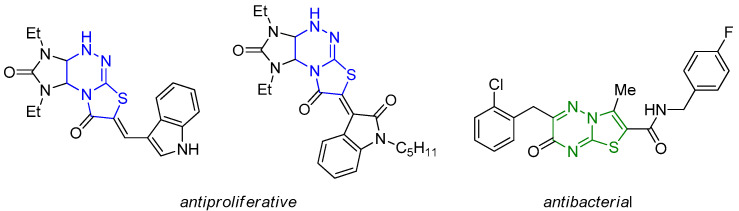
Biologically active thiazolo-1,2,4-triazines.

Over the last decade, our attention was focused on thiazolo-1,2,4-triazines [18,19,20,21,22,23] because these compounds showed antiproliferative and antibacterial activities (Figure 2) [20,21,22,23,24,25].

We previously published the synthesis of hybrids of imidazothiazolotriazine and 2,3′-spiropyrrolidinyloxindole by a 1,3-dipolar cycloaddition of an azomethine ylide generated in situ from isatins and sarcosine to arylmethylidene derivatives of imidazothiazolotriazine [26,27]. The reaction proceeded with high regio- and diastereoselectivity to give only one diastereomer. Here, we report the synthesis, structure and stereochemistry of dispirooxindoles based on imidazothiazolotriazine and 3,3′-spiropyrrolidinyloxindole.

## 2. Results and Discussion

### 2.1. Synthesis and Stereochemistry of Dispirooxindoles Based on Imidazothiazolotriazine and Pyrrolidineoxindole

**Scheme 1:**
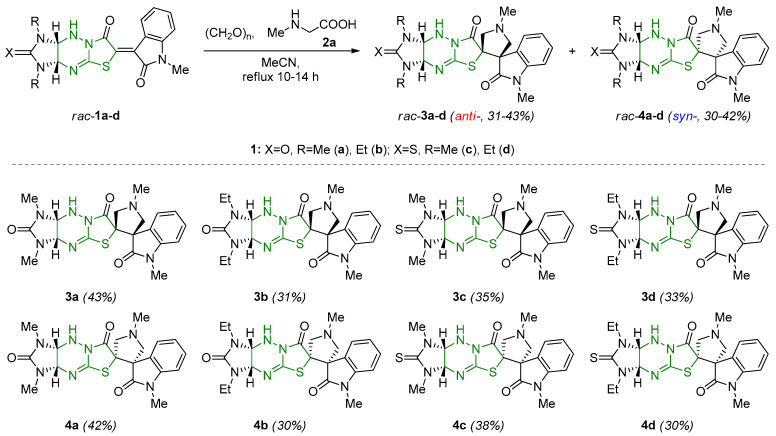
Synthesis of racemic spiropyrrolidineoxindoles **3a**–**d**, **4a**–**d** via 1,3-dipolar cycloaddition using paraformaldehyde and sarcosine as azomethine ylide precursors.

To synthesize target dispirooxindoles we used previously prepared racemic oxindolylideneimidazothiazolotriazines **1a**–**d** [20,28]. The cycloaddition of azomethine ylide generated from paraformaldehyde and sarcosine **2a** to dipolarophiles **1a**–**d** was carried out in acetonitrile under reflux for 10–14 h. According to the NMR spectroscopy data, the reaction proceeded unselectively to afford the mixture of two *ant*i- and *syn*-diastereomers **3** (3aR*,4’R*,6S*,9aS*-**3a**,**b**, 3aR*,4’S*,6R*,9aR*-**3c**,**d**) and **4** (3aR*,4’S*,6R*,9aS*-**4a**,**b**,3aR*,4’R*,6S*,9aR*-**4c**,**d**) in 1:1 ratio (Scheme 1). The mixtures were separated by column chromatography (*^i^*PrOH as eluent), and each of isomers **3a**–**d** and **4a**–**d** was isolated individually in the yields of 31–43 and 30–42%, respectively.

**Scheme 2:**
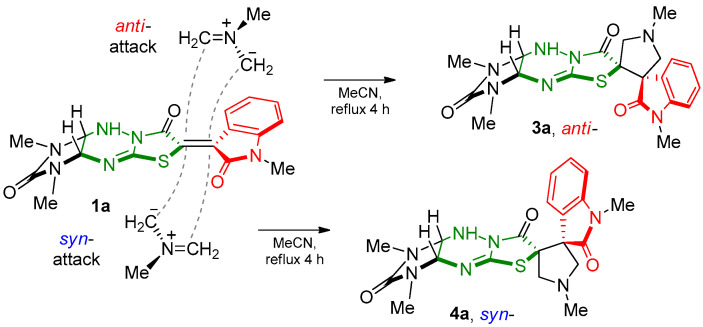
Modes of approach of azomethine ylide.

The stereoselectivity of the cycloaddition can be explained by the possible approach of sterically unhindered “small” azomethine ylide to the plane of the double bond of dipolarophile both from the opposite side (*anti*-) and from the same side (*syn*-) to which the imidazolidine cycle deviates (Scheme 2).

**Scheme 3:**
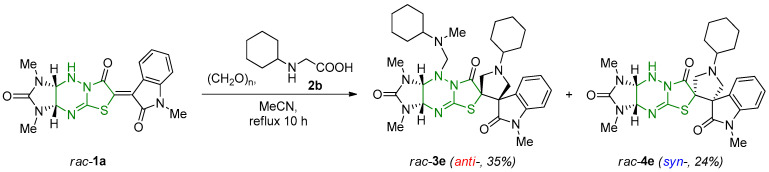
Synthesis of racemic spiropyrrolidineoxindoles **3e** and **4e** via 1,3-dipolar cycloaddition using paraformaldehyde and N-cyclohexylglycine as azomethine ylide precursors.

To increase the selectivity of the reaction we prepared more sterically hindered amino acid, i.e., N-cyclohexylglycine. Indeed, compounds **3** and **4** were obtained in 1.5:1 ratio (Scheme 3). In addition, diastereomer **3** underwent Mannich amino alkylation at the N(9) nitrogen atom. Since compounds **3e** (3aR*,4′R*,6S*,9aS*) and **4e** (3aR*,4′S*,6R*,9aS*) are not diastereomers, they were easily separated by crystallization. Earlier [29], we already observed aminomethylation at the oxindole nitrogen atom during cycloaddition reaction. Thus, we switched to N-methyloxindolylidene derivatives **1** and **5**.

**Scheme 4:**
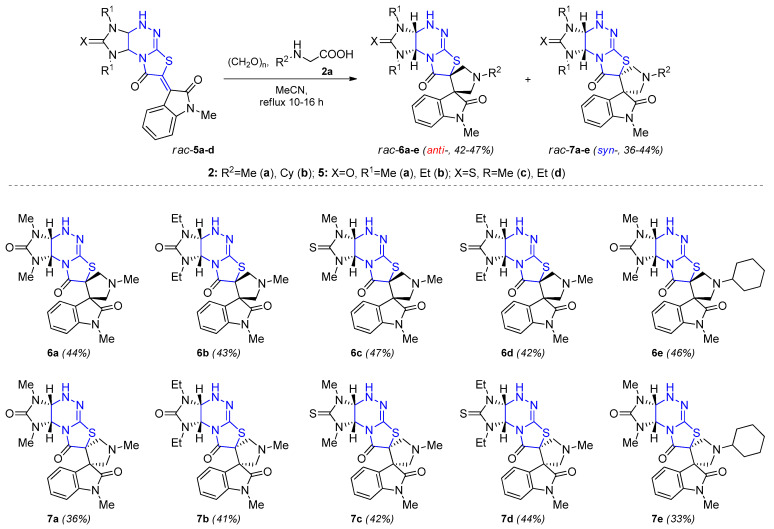
Synthesis of isomeric racemic spiropyrrolidineoxindoles **6a**–**e**, **7a**–**e** via 1,3-dipolar cycloaddition using paraformaldehyde and N-alkylglycine derivatives as azomethine ylide precursors.

Oxindolylidene derivatives of isomeric angular structure **5a**–**d** reacted with paraformaldehyde and amino acids **2a**,**b** similarly to provide the formation of hitherto unavailable *anti*- and *syn*-diastereomers **6** and **7** in approximately the same ratio (Scheme 4). The yields of diastereomers **6** (3aR*,4′R*,7S*,9aS*-**6a**,**b**,**e**, 3aR*,4′R*,7S*,9aR*-**6c**,**d**) and **7** (3aR*,4′S*,7R*,9aS*-**7a**,**b**,**e**, 3aR*,4′S*,7R*,9aR*-**7c**,**d**) after separation by column chromatography were 42–47% and 33–44%, respectively.

**Scheme 5:**
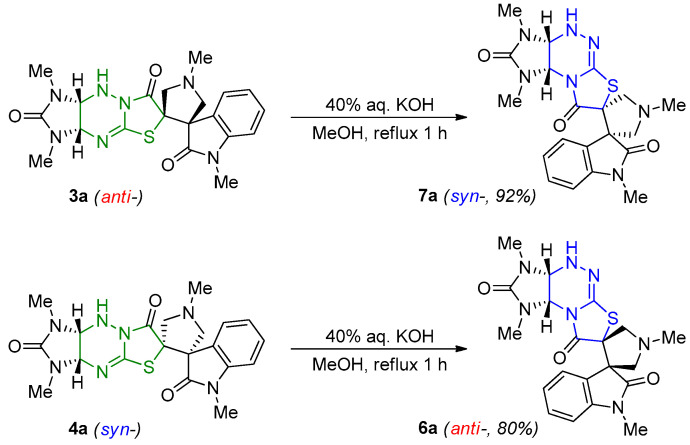
Synthesis of isomeric racemic spiropyrrolidineoxindoles **6a** and **7a** via rearrangement of spiropyrrolidineoxindoles **4a** and **3a**.

Another approach to the compounds **6** and **7** is the rearrangement of isomers **3** and **4** upon treatment with KOH under reflux in methanol. Taking diastereomers **3a** and **4a** as examples, we found that the *anti*-diastereomer **3a** (3aR*,4′R*,6S*,9aS*) underwent rearrangement into the isomeric *syn*-diastereomer **7a** (3aR*,4′S*,7R*,9aS*), while the rearrangement of the *syn*-diastereomer **4a** (3aR*,4′S*,6R*,9aS*) gave rise to the isomeric *anti*-diastereomer **6a** (3aR*,4′R*,7S*,9aS*). The yields of the rearrangement products **6a** and **7a** were 80 and 92%, respectively (Scheme 5).

**Scheme 6:**
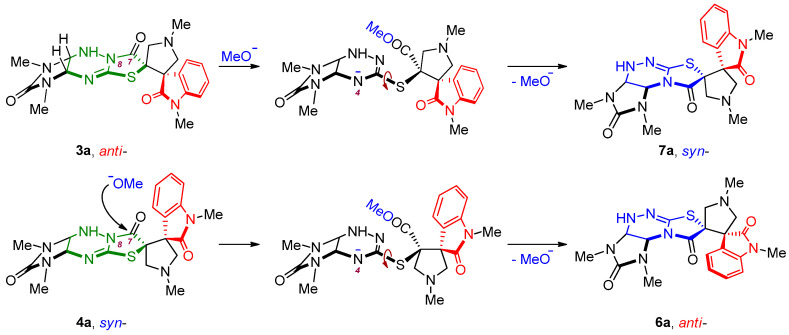
The presumable reaction mechanism of the rearrangement of compounds **3a** and **4a** into isomers **7a** and **6a**.

The presumable rearrangement mechanism is depicted in Scheme 6. We assume that the rearrangement occurs as transamidation reaction upon treatment with KOH in methanol [27]. The nucleophilic attack of the methoxide anion onto the carbon atom C(7) results in the cleavage of the C(7)–N(8) bond followed by rotation of the spiropyrrolidinyloxindole moiety around the C–S bond and the recyclization involving the nitrogen atom N(4) of the triazine ring (Scheme 6).

### 2.2. Structural Determination of Compounds ***3***,***4***,***6***,***7***

The structures of the compounds obtained were elucidated by spectral methods; single crystal X-ray diffraction of **3a**, **4e** and **7c** was also carried out.

**Figure 3:**
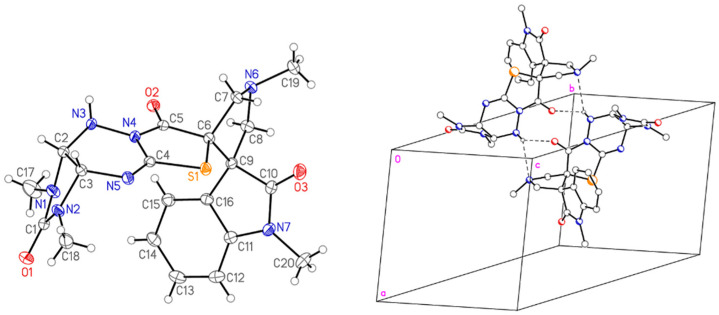
General view and fragment of crystal packing of **3a** in crystal. Anisotropic displacement parameters are drawn at 50% probability level.

**Figure 4:**
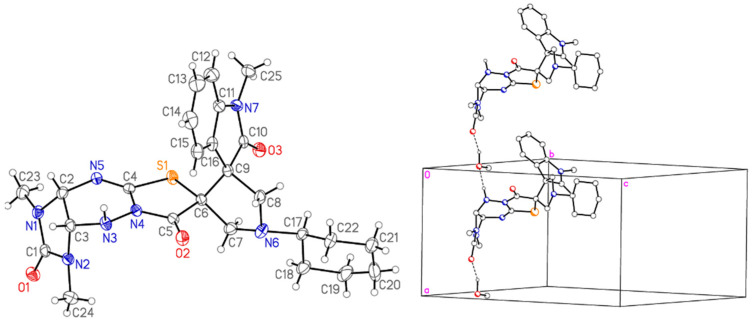
General view and fragment of crystal packing of **4e** in crystal (only one disordered component is shown). Anisotropic displacement parameters are drawn at 20% probability level.

**Figure 5:**
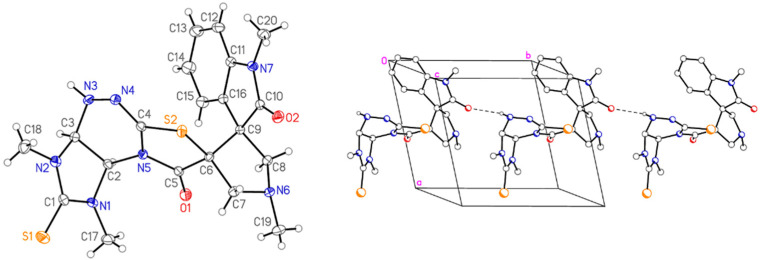
General view and fragment of crystal packing of **7c** in crystal. Anisotropic displacement parameters are drawn at 50% probability level.

Compounds **3a** and **7c** crystalize in centrosymmetric space groups P2_1_/c and $P\bar{1},$ respectively, while **4e** structure crystalizes in an acentric orthorhombic space group P2**_1_**2**_1_**2**_1_** as an inversion twin. Thus, these compounds crystalize as racemic mixtures of two enantiomers. The relative configurations of chiral centers of independent part of compounds **3a** (3aR*,4′R*,6S*,9aS*), **4e** (3aR*,4′S*,6R*,9aS*) and **7c** (3aR*,4′S*,7R*,9aR*) were unambiguously assigned by single crystal X-ray diffraction (Figure 3, Figure 4 and Figure 5).

Bond lengths and angles in the structures are within normal ranges with some exceptions, that was confirmed by the Mogul geometry check [30]. The exceptions are deviations of bond lengths and angles of the oxo- and thioimidazole cycle from average values for similar fragments of the previously published compounds. The list of selected bond lengths and angles can be seen in Table 1.

The five-membered imidazole fragment has an envelope conformation in **3a** (with atom C(2) deviated by 0.46(2) Å) and a twist conformation in **4e** and **7c**. The bicyclic thiazolotriazine fragment is almost planar in **3a** and **4e** with one carbon atom (C(2) and C(3), respectively) deviated by 0.37(3) and 0.47(3) Å from the mean plane, respectively. In **7c** this fragment is significantly twisted with atoms N(3), N(4) and S(2) deviated from the mean plane of C(3)-C(2)-N(5)-C(5)-C(6) atoms by 0.78(3), 0.34(3) and 0.46(3) Å, respectively. The five-membered pyrrolidine fragment has an envelope conformation in **3a**, **4e** and **7c** with atoms C(6), C(6) and C(9) deviated by 0.56(1), 0.57(2) and 0.60(1) Å, respectively. The bridgehead torsion angle H(2)-C(2)-C(3)-H(3) is in the range 30.03–32.86°, which additionally characterizes the distortion of the imidazothiazolotriazine fragments. The mean planes of neighboring spiro-jointed five-membered cycles are practically perpendicular to each other. The nitrogen atoms N(3) and N(6) are slightly pyramidalized with the sum of bond angles at these atoms less than 340° (Table S2).

Presence of H(N) atom allows H-bonding for three solids (Figure 3, Figure 4 and Figure 5). In **3a** centrosymmetric dimers are formed by two bifurcated contacts N(3)…O(2), N(3)…N(6). In **4e** and **7c** infinite chains are observed. For **4e** a solvent methanol acts as a bridge between two molecules.

**Figure 6:**
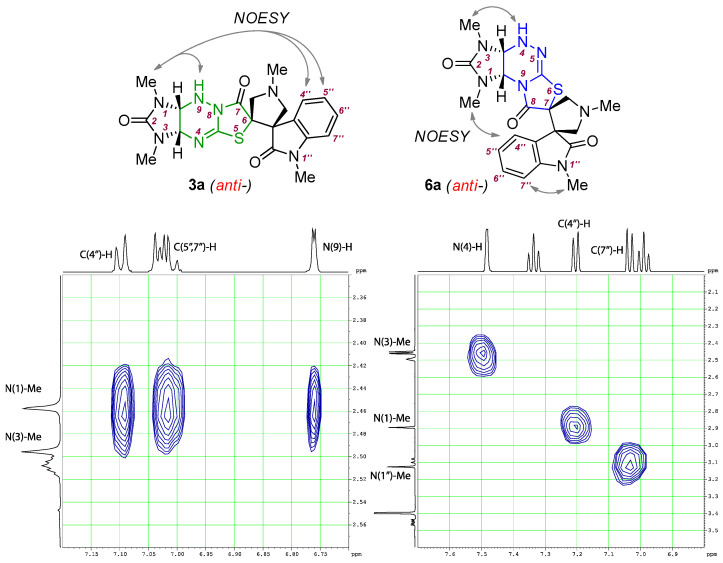
Fragment of the NOESY spectrum of anti-diastereomer **3a** with correlations of N(1)CH_3_-group protons with N(9)H and oxindole fragment H-4″, H-5″ protons.

The structures of compounds **3a**, **3b**, **6a** and **7b** were also confirmed by NMR data in DMSO solutions using NOESY 2D correlation technique. Hydrogen atoms of alkyl group at the nitrogen atom N(1) and hydrogen atoms H-4″, H-5″ of oxindole fragment in compounds **3** are spatially close. The presence of cross-peaks of these protons in the NOESY spectra is characteristic of the *anti*-diastereomers **3a**, **3b**. In the NOESY spectra of *anti*-diastereomer **6a**, correlations between protons of N(1)Me group and H-4″ of oxindole fragment, between protons of N(3)Me and N(4)H groups, as well as between protons H-7″ and N(1′)Me of oxindole fragment were observed. In the NOESY spectrum of *syn*-diastereomer **7b** cross-peaks of NMe group and oxindole fragment protons are absent (see Figure 6 and Supplementary Information).

**Figure 7:**
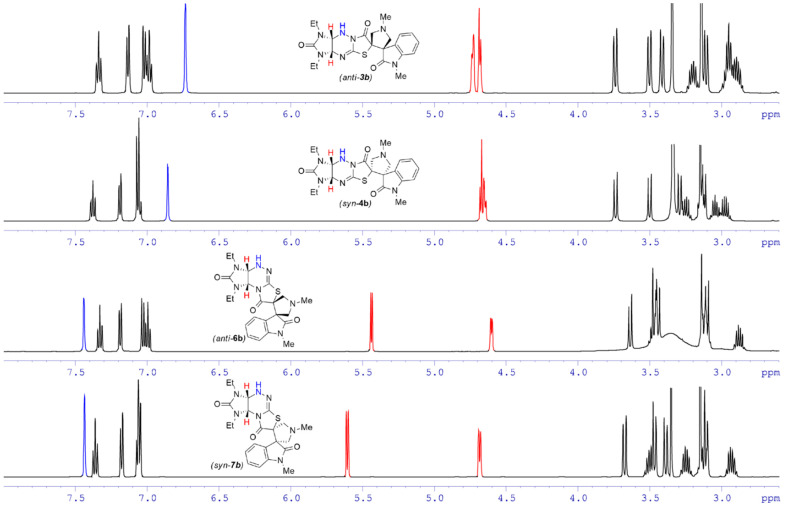
^1^H NMR spectra of compounds **3b**, **4b**, **6b** and **7b** in DMSO-*d*_6_ in the region of 2.6–8.0 ppm.

In the spectra of angular structures **4** and **7**, downfield shifts of the proton signal of the NH group from 6.73–7.05 to 7.43–7.66 ppm were observed in comparison with the spectra of the linear structures **3** and **6**. Downfield shifts from 4.59–4.87 to 5.35–5.85 ppm were also revealed for the signal of one of the bridging protons H(3a) or H(9a) (Figure 7).

Thus, we obtained two diastereomers of each type of dispirocompounds of linear and angular structure in individual form and proved their structures and stereochemistry by spectral methods and single crystal X-ray diffraction.

## 3. Materials and Methods

### 3.1. General Information

Melting points were determined in open glass capillaries on a Gallenkamp (Sanyo) melting point apparatus. IR spectra were recorded on a Bruker ALPHA instrument in KBr pellets. High resolution mass spectra (HRMS) were measured on a Bruker micrOTOF II instrument using electrospray ionization (ESI). The measurements were done in a positive ion mode (interface capillary voltage—4500 V) or in a negative ion mode (3200 V); mass range from *m/z* 50 to *m/z* 3000 Da; external or internal calibration was done with electrospray calibrant solution (Fluka). A syringe injection was used for solutions in acetonitrile or methanol (flow rate 3 mL min^−1^). Nitrogen was applied as a dry gas; the interface temperature was set at 180 °C. ^1^H NMR and ^13^C NMR spectra were recorded on a Bruker AM300 spectrometer operating at 300.13 MHz and 75.5 MHz, respectively, Bruker DRX 500 spectrometer (500.13 MHz and 125.76 MHz, respectively) and Bruker DRX 600 spectrometer (Bruker, USA; 600.13 MHz and 150.90 MHz, respectively) using DMSO-*d*_6_ as a solvent. Chemical shifts (*δ*) are given in ppm from TMS as an internal standard. All reactions were run in air.

Column chromatography was carried out with silica gel (Acros Silica gel, for column chromatography, 60 Angstrom pore size, 0.040–0.063 mm) and to monitor the preparative separations, analytical thin layer chromatography (TLC) was performed at room temperature on pre-coated 0.25 mm thick silica gel 60 F_254_ aluminum plates 20 × 20 cm Merck, Darmstadt, Germany. Propanol-2 was used as eluent without preliminary purification.

The X-ray data collection for samples **3a** and **7c** were performed on a Bruker APEX DUO diffractometer equipped with an Apex II CCD detector (graphite monochromator, ω-scans), and for sample **4e** on a Bruker Quest D8 diffractometer equipped with a Photon-III area-detector (graphite monochromator, shutterless φ- and ω-scan technique), using Mo Kα-radiation (λ = 0.71073 Å).

The intensity data were integrated by the Bruker SAINT software package [31] and were corrected for absorption and decay using the SADABS program [32]. The structures were solved using the SHELXT program [33] and refined by the full-matrix least-squares technique against F^2^_hkl_ in anisotropic approximation for non-hydrogen atoms with the SHELXL program [34]. Hydrogen atoms connected to heteroatoms were found from difference Fourier synthesis and refined isotropically. Other hydrogen atoms were placed in calculated positions and refined in the riding model with U_iso_(H) = 1.5U_eq_(C_m_) for methyl groups and 1.2U_eq_(C_i_) for other carbon atoms to which corresponding H atoms are bonded. The structure **4e** was refined as an inversion twin with calculated BASF parameter equal to 0.58. The cyclohexyl fragment in **4e** was found to be disordered by two positions with refined relative occupancies 0.68:0.32, ISOR instruction was used for the disordered atoms. For **4e** a SQUEEZE method implemented in the PLATON program [35] was applied to model the disordered methanol molecule.

Detailed crystallographic information is given in Table S1. CCDC 2215817-2215819 contain supplementary crystallographic data for this paper. The data can be obtained free of charge from The Cambridge Crystallographic Data Centre via www.ccdc.cam.ac.uk/structures (accessed on 20 October 2022).

### 3.2. General Procedure for the Preparation of Dispiro[imidazo[4,5-e]thiazolo[3,2-b][1,2,4]triazine-6,3′-pyrrolidine-4′,3′′-indole] ***3a**–**e*** and ***4a**–**e***

Dipolarophile **1a**–**d** (0.5 mmol), paraformaldehyde (0.060 g, 2.0 mmol) and N-alkylglycine **1a**,**b** (2.0 mmol) were charged in a flask and acetonitrile (40 mL) was added. The resulting suspension was refluxed with stirring for 10–14 h until the red color disappeared. The solvent was then removed under reduced pressure to afford a solid product. This solid was dissolved in isopropyl alcohol (10 mL) and separated via column chromatography using isopropyl alcohol as eluent. *Anti*-diastereomers **3a**–**e** have a retention factor *R_f_* = 0.20–0.39, *syn*-diastereomers **4a**–**e** have a retention factor *R_f_* = 0.43–0.60.

(3aR*,4′R*,6S*,9aS*)-1,**1′**,**1″**,**3-Tetramethyl-3**,**3a**,**9**,**9a-tetrahydro-7*H*-dispiro[imidazo[4**,**5-*e*]thiazolo[3**,**2-*b*][1,2,4]triazine-6**,**3′-pyrrolidine-4′**,**3″-indoline]-2**,**2″**,**7(1*H*)-trione (3a).** Yield 95 mg (43%); *R_f_* = 0.21; white solid; mp: 280–282 °C. IR (KBr): ν 3414 (NH), 3065, 3055 (ArH), 2936, 2861 (Alk), 1700, 1653, 1644, 1611 (C=O, C=N) cm^−1^. ^1^H NMR (500 MHz, DMSO-*d_6_*): δ 2.45 (s, 3H, NCH_3_), 2.49 (s, 3H, 1′-NCH_3_), 2.59 (s, 3H, NCH_3_), 3.11 (d, *J* = 10.2 Hz, 1H, CH_2_), 3.14 (s, 3H, 1′′-NCH_3_), 3.40 (d, *J* = 10.2 Hz, 1H, CH_2_), 3.48 (d, *J* = 9.9 Hz, 1H, CH_2_), 3.74 (d, *J* = 10.5 Hz, 1H, CH_2_), 4.56–4.60 (m, 2H, 3a-H, 9a-H), 6.75 (d, *J* = 1.8 Hz, 1H, 9-NH), 6.98–7.03 (m, 2H, 5′′-H, 7′′-H), 7.09 (d, *J* = 7.2 Hz, 1H, 4′′-H), 7.34 (t, *J* = 6.9 Hz, 1H, 6′′-H). ^13^C NMR (75 MHz, DMSO-*d_6_*): δ 26.07, 26.64, 27.54 (1,1′′,3-NCH_3_), 42.03 (1′-NCH_3_), 60.04, 60.20 (C-3′, C-3′′), 61.89, 63.96 (C-2′, C-5′), 64.73, 65.54 (C-3a, C-9a), 109.12 (C-7′′), 122.54, 123.14, 123.88, 129.54 (C-3a′′, C-4′′, C-5′′, C-6′′), 144.41 (C-7a′′), 148.60 (4a-C=N), 158.26 (2-C=O), 168.50 (7-C=O), 176.12 (2′′-C=O). HRMS (ESI): Calculated for C_20_H_23_N_7_O_3_S [M + H]^+^: 442.1647, Found: 442.1656.

(3aR*,4′R*,6S*,9aS*)-1,**3-Diethyl-1′**,**1″-dimethyl-3**,**3a**,**9**,**9a-tetrahydro-7*H*-dispiro[imidazo[4**,**5-*e*]thiazolo[3**,**2-*b*][1,2,4]triazine-6**,**3′-pyrrolidine-4′**,**3″-indoline]-2**,**2″**,**7(1*H*)-trione (3b).** Yield 73 mg (31%); *R_f_* = 0.24; white solid; mp: 252–254 °C. IR (KBr): ν 3245, 3163 (NH), 3063, 3015 (ArH), 2967, 2873, 2876 (Alk), 1756, 1676, 1682, 1635 (C=O, C=N) cm^−1^. ^1^H NMR (500 MHz, DMSO-*d_6_*): δ 0.88 (t, *J* = 6.9 Hz, 3H, CH_3_), 1.01 (t, *J* = 7.2 Hz, 3H, CH_3_), 2.48 (s, 3H, 1′-NCH_3_), 2.85–2.99 (m, 3H, NCH_2_), 3.10 (d, *J* = 10.2 Hz, CH_2_), 3.13 (s, 3H, 1′′-NCH_3_), 3.16–3.23 (m, 1H, NCH_2_), 3.41 (d, *J* = 10.5 Hz, 1H, CH_2_), 3.50 (d, *J* = 10.1 Hz, 1H, CH_2_), 3.73 (d, *J* = 10.4 Hz, 1H, CH_2_), 4.68 (d, *J* = 5.8 Hz, 1H, 3a-H), 4.72 (d, *J* = 5.6 Hz, 1H, 9a-H), 6.73 (s, 1H, 9-NH), 6.96–7.02 (m, 2H, 5′′-H, 7′′-H), 7.13 (d, *J* = 7.4 Hz, 1H, 4′′-H), 7.33 (t, *J* = 7.7 Hz, 1H, 6′′-H). ^13^C NMR (150 MHz, DMSO-*d_6_*): δ 13.43, 14.45 (2CH_3_), 27.28 (1′′-NCH_3_), 35.00, 35.85 (1,3-NCH_2_), 43.24 (1′-NCH_3_), 60.98, 61.56 (C-3′, C-3′′), 63.37 (C-2′, C-5′), 64.39, 65.88 (C-3a, C-9a), 110.28 (C-7′′), 123.89, 124.58, 125.58, 130.69 (C-3a′′, C-4′′, C-5′′, C-6′′), 145.62 (4a-C=N), 149.93 (C-7a′′), 158.58 (2-C=O), 169.61 (7-C=O), 177.43 (2′′-C=O). HRMS (ESI): Calculated for C_22_H_27_N_7_O_3_S [M + H]^+^: 470.1974, Found: 470.1969.

(3aR*,4′S*,6R*,9aR*)-1,1′,1″,3-Tetramethyl-2-thioxo-1,2,3,3a,9,9a-hexahydro-7*H*-dispro[imidazo[4,5-*e*]thiazolo[3,2-*b*][1,2,4]triazine-6,3′-pyrrolidine-4′,3″-indoline]-2″,7-dione (3c). Yield 80 mg (35%); *R_f_* = 0.20; white solid; mp: 228–230 °C. IR (KBr): ν 3513 (NH), 3024 (ArH), 2966, 2927, 2849 (Alk), 1733, 1708, 1633, 1615 (C=O, C=N) cm^−1^. ^1^H NMR (300 MHz, DMSO-*d_6_*): δ 2.47 (s, 3H, 1′-NCH_3_), 2.89 (s, 3H, NCH_3_), 2.99 (s, 3H, NCH_3_), 3.10–3.14 (m, 4H, 1′′-NCH_3_, CH_2_), 3.37 (d, *J* = 10.8 Hz, 1H, CH_2_), 3.46 (d, *J* = 9.9 Hz, 1H, CH_2_), 3.70 (d, *J* = 10.5 Hz, 1H, CH_2_), 4.82–4.89 (m, 2H, 3a-H, 9a-H), 7.03–7.07 (m, 3H, 5′′-H, 7′′-H, 9-NH), 7.17 (d, *J* = 7.5 Hz, 1H, 4′′-H), 7.37 (t, *J* = 7.5 Hz, 1H, 6′′-H). ^13^C NMR (75 MHz, DMSO-*d_6_*): δ 26.07 (1′′-NCH_3_), 30.80, 31.05 (1,3-NCH_3_), 41.92 (1′-NCH_3_), 60.23, 60.43 (C-3′, C-3′′), 61.60, 63.02 (C-2′, C-5′), 66.79, 67.84 (C-3a, C-9a), 108.96 (C-7′′), 122.98, 123.11, 124.27, 129.65 (C-3a′′, C-4′′, C-5′′, C-6′′), 144.53 (C-7a′′), 149.30 (4a-C=N), 167.82 (7-C=O), 175.96 (2′′-C=O), 182.69 (2-C=S). HRMS (ESI): Calculated for C_20_H_23_N_7_O_2_S_2_ [M + H]^+^: 458.1427, Found: 458.1427.

(3aR*,4′S*,6R*,9aR*)-1,3-Diethyl-1′,1″-dimethyl-2-thioxo-1,2,3,3a,9,9a-hexahydro-7*H*-dispiro[imidazo[4,5-*e*]thiazolo[3,2-*b*][1,2,4]triazine-6,3′-pyrrolidine-4′,3″-indoline]-2″,7-dione (3d). Yield 80 mg (33%); *R_f_* = 0.25; white solid; mp: 155–157 °C. IR (KBr): ν 3433 (NH), 3233 (ArH), 2973, 2939, 2876 (Alk), 1707, 1641, 1613 (C=O, C=N) cm^−1^. ^1^H NMR (300 MHz, DMSO-*d_6_*) δ 0.92 (t, *J* = 7.0 Hz, 3H, CH_3_), 1.07 (t, *J* = 7.0 Hz, 3H, CH_3_), 2.49 (s, 3H, 1′-NCH_3_), 3.10–3.32 (m, 6H, 1′′-NCH_3_, CH_2_), 3.41–3.51 (m, 3H, CH_2_), 3.71–3.81 (m, 2H, CH_2_), 4.88 (d, *J* = 6.7 Hz, 1H, 3a-H), 5.03 (dd, *J* = 6.7, 2.4 Hz, 1H, 9a-H), 6.85 (d, *J* = 2.3 Hz, 1H, 9-NH), 6.95–7.02 (m, 2H, 5′′-H, 7′′-H), 7.12 (d, *J* = 7.5 Hz, 1H, 4′′-H), 7.33 (t, *J* = 7.6 Hz, 1H, 6′′-H). ^13^C NMR (75 MHz, DMSO-*d_6_*): δ 11.63, 12.84 (2CH_3_), 26.07 (1′′-NCH_3_), 37.63, 37.88 (1,3-NCH_2_), 42.02 (1′-NCH_3_), 59.70, 60.33 (C-3′, C-3′′), 62.06, 63.82, 64.56, 65.84 (2CH_2_, C-3a, C-9a), 109.09 (C-7′′), 122.74, 123.20, 124.28, 129.51 (C-3a′′, C-4′′, C-5′′, C-6′′), 144.34 (4a-C=N), 149.99 (C-7a′′), 168.24 (7-C=O), 176.16 (2′′-C=O), 180.72 (2-C=S). HRMS (ESI): Calculated for C_22_H_27_N_7_O_2_S_2_ [M + H]^+^: 486.1729, Found: 486.1740.

(3aR*,4′R*,6S*,9aS*)-1′-Cyclohexyl-9-((cyclohexyl(methyl)amino)methyl)-1,1″,3-trimethyl-3,3a,9,9a-tetrahydro-7*H*-dispiro[imidazo[4,5-*e*]thiazolo[3,2-*b*][1,2,4]triazine-6,3′-pyrrolidine-4′,3″-indoline]-2,2″,7(1*H*)-trione (3e). Yield 110 mg (35%); *R_f_* = 0.39; white solid; mp: 179–181 °C. IR (KBr): ν 2930, 2854 (Alk), 1709, 1641, 1610 (C=O, C=N) cm^−1^. ^1^H NMR (300 MHz, DMSO-*d_6_*): δ 1.14–1.28 (m, 10H, Cy), 1.52–1.83 (m, 10H, Cy), 2.36–2.41 (m, 4H, NCH_3_, Cy), 2.47 (s, 3H, NCH_3_), 2.60–2.63 (m, 4H, NCH_3_, Cy), 3.15 (s, 3H, 1′′-NCH_3_), 3.20 (d, *J* = 9.9 Hz, 1H, CH_2_), 3.47–3.57 (m, 2H, CH_2_), 3.72 (d, *J* = 12.7 Hz, 1H, 9-NCH_2_), 3.79 (d, *J* = 10.0 Hz, 1H, CH_2_), 4.05 (d, *J* = 12.7 Hz, 1H, 9-NCH_2_), 4.70 (d, *J* = 5.8 Hz, 1H, 9a-H), 4.76 (d, *J* = 5.7 Hz, 1H, 3a-H), 6.99–7.05 (m, 2H, 5′′-H, 7′′-H), 7.11 (d, *J* = 7.4 Hz, 1H, 4′′-H), 7.35 (t, *J* = 7.6 Hz, 1H, 6′′-H). ^13^C NMR (75 MHz, DMSO-*d_6_*): δ 23.78, 23.93, 25.49, 25.59, 26.01, 26.61, 27.41, 28.26, 28.92, 30.88, 31.31, 36.23 (4NCH_3_, Cy), 57.40, 58.80, 59.29, 59.74, 60.17, 62.06, 63.46, 68.70, 72.42 (C-2′, C-3′, C-3′′, C-5′, C-3a, C-9a, 9-NCH_2_, 2Cy), 109.00 (C-7′′), 122.43, 123.33, 124.09, 129.47 (C-3a′′, C-4′′, C-5′′, C-6′′), 144.41 (C-7a′′), 147.85 (4a-C=N), 157.80 (2-C=O), 167.59 (7-C=O), 175.97 (2′′-C=O). HRMS (ESI): Exact mass calcd for C_33_H_46_N_8_O_3_S [M + H]^+^: 635.3486, Found: 635.3497.

(3aR*,4′S*,6R*,9aS*)-1,1′,1″,3-Tetramethyl-3,3a,9,9a-tetrahydro-7*H*-dispiro[imidazo[4,5-*e*]thiazolo[3,2-*b*][1,2,4]triazine-6,3′-pyrrolidine-4′,3″-indoline]-2,2″,7(1*H*)-trione (4a). Yield 92 mg (42%); *R_f_* = 0.53; white solid; mp: 164–166 °C. IR (KBr): ν 3468, 3188 (NH), 3053, 3026 (ArH), 2934, 2854 (Alk), 1716, 1700, 1644 (C=O, C=N) cm^−1^. ^1^H NMR (500 MHz, DMSO-*d_6_*): δ 2.47 (s, 3H, 1′-NCH_3_), 2.57 (s, 3H, NCH_3_), 2.64 (s, 3H, NCH_3_), 3.11 (d, *J* = 10.2 Hz, 1H, CH_2_), 3.14 (s, 3H, 1′′-NCH_3_), 3.40 (d, *J* = 10.7 Hz, 1H, CH_2_), 3.47 (d, *J* = 10.2 Hz, 1H, CH_2_), 3.71 (d, *J* = 10.7 Hz, 1H, CH_2_), 4.52 (dd, *J* = 5.7, 2.5 Hz, 1H, 9a-H), 4.60 (d, *J* = 5.8 Hz, 1H, 3a-H), 6.83 (d, *J* = 2.1 Hz, 1H, 9-NH), 7.05–7.06 (m, 2H, 5′′-H, 7′′-H), 7.17 (d, *J* = 7.4 Hz, 1H, 4′′-H), 7.37 (t, *J* = 7.6 Hz, 1H, 6′′-H). ^13^C NMR (75 MHz, DMSO-*d_6_*): δ 26.07, 26.71, 27.69 (1,1′′,3-NCH_3_), 41.96 (1′-NCH_3_), 60.42, 61.67, 63.20 (C-2′, C-3′, C-3′′, C-5′), 65.03, 65.64 (C-3a, C-9a), 108.96 (C-7′′), 123.06, 123.10, 124.29, 129.66 (C-3a′′, C-4′′, C-5′′, C-6′′), 144.58 (C-7a′′), 148.24 (4a-C=N), 158.62 (2-C=O), 167.97 (7-C=O), 176.05 (2′′-C=O). HRMS (ESI): Calculated for C_20_H_23_N_7_O_3_S [M + H]^+^: 442.1665, Found: 442.1656.

(3aR*,4′S*,6R*,9aS*)-1,3-Diethyl-1′,1″-dimethyl-3,3a,9,9a-tetrahydro-7*H*-dispiro[imidazo[4,5-*e*]thiazolo[3,2-*b*][1,2,4]triazine-6,3′-pyrrolidine-4′,3″-indoline]-2,2″,7(1*H*)-trione (4b). Yield 70 mg (30%); *R_f_* = 0.60; white solid; mp: 217–219 °C. IR (KBr): ν 3495, 3175 (NH), 3057 (ArH), 2980, 2959, 2935, 2898, 2874 (Alk), 1738, 1709, 1693, 1634, 161 (C=O, C=N) cm^−1^. ^1^H NMR (500 MHz, DMSO-*d_6_*): δ 0.95 (t, *J* = 7.1 Hz, 3H, CH_3_), 1.04 (t, *J* = 7.2 Hz, 3H, CH_3_), 2.47 (s, 3H, 1′-NCH_3_), 2.93–3.07 (m, 2H, NCH_2_), 3.10–3.17 (m, 5H, 1′′-NCH_3_, CH_2_, NCH_2_), 3.21–3.30 (m, 2H, CH_2_, NCH_2_), 3.49 (d, *J* = 10.2 Hz, 1H, CH_2_), 3.73 (d, *J* = 10.6 Hz, 1H, CH_2_), 4.63–4.68 (m, 2H, 3a-H, 9a-H), 6.85 (d, *J* = 2.2 Hz, 1H, 9-NH), 7.04–7.07 (m, 2H, 5′′-H, 7′′-H), 7.18 (d, *J* = 7.3 Hz, 1H, 4′′-H), 7.37 (t, *J* = 7.7 Hz, 1H, 6′′-H). ^13^C NMR (125 MHz, DMSO-*d_6_*): δ 13.22, 13.28 (2CH_3_), 26.15 (1′′-NCH_3_), 34.25, 35.29 (1,3-NCH_2_), 42.07 (1′-NCH_3_), 60.36, 60.57 (C-3′, C-3′′), 61.76, 63.00, 63.53, 64.35 (C-2′, C-3a, C-5′, C-9a), 109.05 (C-7′′), 123.11, 123.20, 124.45, 129.75 (C-3a′′, C-4′′, C-5′′, C-6′′), 144.69 (4a-C=N), 148.17 (C-7a′′), 157.84 (2-C=O), 168.00 (7-C=O), 176.14 (2′′-C=O). HRMS (ESI): Calculated for C_22_H_27_N_7_O_3_S [M + H]^+^: 470.1965, Found: 470.1969.

(3aR*,4′R*,6S*,9aR*)-1,1′,1″,3-Tetramethyl-2-thioxo-1,2,3,3a,9,9a-hexahydro-7*H*-dispiro[imidazo[4,5-*e*]thiazolo[3,2-*b*][1,2,4]triazine-6,3′-pyrrolidine-4′,3″-indoline]-2″,7-dione (4c). Yield 87 m (38%); *R_f_* = 0.43; white solid; mp: 273–275 °C. IR (KBr): ν 3399 (NH), 3125 (ArH), 2936, 2921, 2855 (Alk), 1726, 1707, 1603, 1609 (C=O, C=N) cm^−1^. ^1^H NMR (300 MHz, DMSO-*d_6_*): δ 2.51 (s, 3H, 1′-NCH_3_), 2.79 (s, 3H, NCH_3_), 2.94 (s, 3H, NCH_3_), 3.12–3.16 (m, 4H, CH_2_, 1′′-NCH_3_), 3.42 (d, *J* = 10.5 Hz, 1H, CH_2_), 3.49 (d, *J* = 10.2 Hz, 1H, CH_2_), 3.75 (d, *J* = 10.5 Hz, 1H, CH_2_), 4.85 (d, *J* = 6.3 Hz, 1H, 3a-H), 4.92 (d, *J* = 6.3 Hz, 1H, 9a-H), 6.95 (s, 1H, 9-NH), 7.02–7.11 (m, 3H, 4′′-H, 5′′-H, 7′′-H), 7.35 (t, *J* = 7.5 Hz, 1H, 6′′-H). ^13^C NMR (75 MHz, DMSO-*d_6_*): δ 26.06 (1′′-NCH_3_), 30.62, 30.95 (1,3-NCH_3_), 41.98 (1′-NCH_3_), 59.98, 60.15 (C-3′, C-3′′), 61.80, 63.88 (C-2′, C-5′), 66.41, 67.88 (C-3a, C-9a), 109.11 (C-7′′), 122.62, 123.01, 123.72, 129.54 (C-3a′′, C-4′′, C-5′′, C-6′′), 144.34 (C-7a′′), 149.80 (4a-C=N), 168.35 (7-C=O), 176.05 (2′′-C=O), 182.36 (2-C=S). HRMS (ESI): Calculated for C_20_H_23_N_7_O_2_S_2_ [M + H]^+^: 458.1426, Found: 458.1427.

(3aR*,4′R*,6S*,9aR*)-1,3-Diethyl-1′,1″-dimethyl-2-thioxo-1,2,3,3a,9,9a-hexahydro-7*H*-dispiro[imidazo[4,5-*e*]thiazolo[3,2-*b*][1,2,4]triazine-6,3′-pyrrolidine-4′,3″-indoline]-2″,7-dione (4d). Yield 73 mg (30%); *R_f_* = 0.54; white solid; mp: 224–226 °C. IR (KBr): ν 3433 (NH), 3261, 3210 (ArH), 2969, 2932, 2858 (Alk), 1728, 1701, 1641, 1612 (C=O, C=N) cm^−1^. ^1^H NMR (300 MHz, DMSO-*d_6_*): δ 0.99 (t, *J* = 6.9 Hz, 3H, CH_3_), 1.11 (t, *J* = 7.1 Hz, 3H, CH_3_), 2.46 (s, 3H, 1′-NCH_3_), 3.10–3.14 (m, 4H, 1′′-NCH_3_, CH_2_), 3.19–3.34 (m, 2H, NCH_2_, CH_2_), 3.41–3.56 (m, 3H, NCH_2_, CH_2_), 3.71–3.83 (m, 2H, NCH_2_, CH_2_), 4.88 (d, *J* = 6.6 Hz, 1H, 3a-H), 4.96 (dd, *J* = 6.7, 2.7 Hz, 1H, 9a-H), 7.03–7.07 (m, 3H, 5′′-H, 7′′-H, 9-NH), 7.18 (d, *J* = 7.6 Hz, 1H, 4′′-H), 7.37 (t, *J* = 7.7 Hz, 1H, 6′′-H). ^13^C NMR (75 MHz, DMSO-*d_6_*): δ 12.30, 12.92 (2CH_3_), 26.14 (1′′-NCH_3_), 38.24, 38.34 (1,3-NCH_2_), 42.04 (1′-NCH_3_), 60.36 (C-3′, C-3′′), 61.67, 63.30 (C-2′, C-5′), 64.73, 66.71 (C-3a, C-9a), 109.06 (C-7′′), 123.00, 123.19, 124.43, 129.79 (C-3a′′, C-4′′, C-5′′, C-6′′), 144.62 (4a-C=N), 149.24 (C-7a′′), 167.87 (7-C=O), 176.06 (2′′-C=O), 181.23 (2-C=S). HRMS (ESI): Calculated for C_22_H_27_N_7_O_2_S_2_ [M + H]^+^: 486.1749, Found: 486.1740.

(3aR*,4′S*,6R*,9aS*)-1′-Cyclohexyl-1,1″,3-trimethyl-3,3a,9,9a-tetrahydro-7*H*-dispiro[imidazo[4,5-*e*]thiazolo[3,2-*b*][1,2,4]triazine-6,3′-pyrrolidine-4′,3″-indoline]-2,2″,7(1*H*)-trione (4e). Yield 49 mg (24%); *R_f_* = 0.56; white solid; mp: 217–219 °C. IR (KBr): ν 3434, 3175 (NH), 2931, 2854 (Alk), 1706, 1638, 1612 (C=O, C=N) cm^−1^. ^1^H NMR (300 MHz, DMSO-*d_6_*): δ 1.12–1.24 (m, 5H, Cy), 1.48–1.52 (m, 1H, Cy), 1.61–1.70 (m, 2H, Cy), 1.76–1.81 (m, 2H, Cy), 2.56–2.59 (m, 4H, NCH_3_, Cy), 2.65 (s, 3H, NCH_3_), 3.14 (s, 3H, 1′′-NCH_3_), 3.19 (d, *J* = 10.0 Hz, 1H, CH_2_), 3.42–3.50 (m, 2H, CH_2_), 3.72 (d, *J* = 10.3 Hz, 1H, CH_2_), 4.52 (dd, *J* = 5.9, 2.5 Hz, 1H, 9a-H), 4.60 (d, *J* = 5.8 Hz, 1H, 3a-H), 6.80 (d, *J* = 2.5 Hz, 1H, 9-NH), 7.02–7.06 (m, 2H, 5′′-H, 7′′-H), 7.17 (d, *J* = 7.2 Hz, 1H, 4′′-H), 7.36 (t, *J* = 7.4 Hz, 1H, 6′′-H). ^13^C NMR (75 MHz, DMSO-*d_6_*): δ 24.45, 24.59, 26.15, 26.57, 27.21, 28.26, 31.45, 31.89 (3NCH_3_, Cy), 57.77, 59.70, 59.88, 60.10, 61.09, 65.46, 66.14 (C-2′, C-3′, C-3′′, C-5′, C-3a, C-9a, Cy), 109.41 (C-7′′), 123.50, 123.86, 124.84, 130.11 (C-3a′′, C-4′′, C-5′′, C-6′′), 145.11 (C-7a′′), 148.85 (4a-C=N), 159.13 (2-C=O), 168.39 (7-C=O), 176.38 (2′′-C=O). HRMS (ESI): Calculated for C_25_H_31_N_7_O_3_S [M + H]^+^: 510.2282, Found: 510.2274.

### 3.3. General Procedure for the Preparation of Dispiro[imidazo[4,5-e]thiazolo[2,3-c][1,2,4]triazine-7,3′-pyrrolidine-4′,3″-indole] ***6a**–**e*** and ***7a**–**e***

*Method A*. Dipolarophile **5a**–**d** (0.5 mmol), paraformaldehyde (0.060 g, 2.0 mmol) and N-alkylglycine **1a**,**b** (2.0 mmol) were charged in a flask and acetonitrile (40 mL) was added. The resulting suspension was refluxed with stirring for 10–16 h until the red color disappeared. The solvent was then removed under reduced pressure to afford a solid product. This solid was dissolved in isopropyl alcohol (10 mL) and separated via column chromatography using isopropyl alcohol as eluent. *Anti*-diastereomers **6a**–**e** have a retention factor *R_f_* = 0.35–0.54, *syn*-diastereomers **7a**–**e** have a retention factor *R_f_* = 0.58–0.73.

*Method B.* To a stirred suspension of compound **3a** (0.442 g, 0.1 mmol) or **4a** (0.442 g, 0.1 mmol) in refluxing methanol (2 mL), 0.010 mL of 40% aqueous KOH (0.1 mmol) was added. The resulting mixture was refluxed with stirring for 1 h. After cooling, the precipitate of compounds **7a** or **6a** was filtered off, washed with methanol and dried.

(3aR*,4′R*,7S*,9aS*)-1,1′,1″,3-Tetramethyl-1,3a,4,9a-tetrahydro-8*H*-dispiro[imidazo[4,5-*e*]thiazolo[2,3-*c*][1,2,4]triazine-7,3′-pyrrolidine-4′,3″-indoline]-2,2″,8(3*H*)-trione (6a). Method A: yield 97 mg (44%); method B: yield 41 mg (92%); *R_f_* = 0.35; white solid; mp: 218–220 °C. IR (KBr): ν 3414 (NH), 3429, 3310 (ArH), 2967, 2941, 2853 (Alk), 1727, 1684, 1651 (C=O, C=N) cm^−1^. ^1^H NMR (500 MHz, DMSO-*d_6_*): δ 2.46 (s, 3H, NCH_3_), 2.47 (s, 3H, 1′-NCH_3_), 2.90 (s, 3H, NCH_3_), 3.10 (d, *J* = 10.2 Hz, 1H, CH_2_), 3.13 (s, 3H, 1′′-NCH_3_), 3.44–3.47 (m, 2H, CH_2_), 3.65 (d, *J* = 10.6 Hz, 1H, CH_2_), 4.54 (dd, *J* = 5.6, 2.0 Hz, 1H, 3a-H), 5.36 (d, *J* = 5.7 Hz, 1H, 9a-H), 6.98 (t, *J* = 7.5 Hz, 1H, 5′′-H), 7.03 (d, *J* = 7.8 Hz, 1H, 7′′-H), 7.19 (d, *J* = 7.5 Hz, 1H, 4′′-H), 7.33 (t, *J* = 7.7 Hz, 1H, 6′′-H), 7.47 (d, *J* = 1.9 Hz, 1H, 4-NH). ^13^C NMR (75 MHz, DMSO-*d_6_*): δ 26.02, 27.87, 31.62 (1,1′′,3-NCH_3_), 41.88 (1′-NCH_3_), 60.29, 61.67, 62.54, 63.77, 64.77, 66.24 (C-2′, C-3a, C-3′, C-3′′, C-5′, C-9a), 108.88 (C-7′′), 122.43, 123.52, 124.66, 129.50 (C-3a′′, C-4′′, C-5′′, C-6′′), 134.15 (5a-C=N), 144.42 (C-7a′′), 159.25 (2-C=O), 171.97 (8-C=O), 176.25 (2′′-C=O). HRMS (ESI): Calculated for C_20_H_23_N_7_O_3_S [M + H]^+^: 442.1655, Found: 442.1656.

(3aR*,4′R*,7S*,9aS*)-1,3-Diethyl-1′,1″-dimethyl-1,3a,4,9a-tetrahydro-8*H*-dispiro[imidazo[4,5-*e*]thiazolo[2,3-*c*][1,2,4]triazine-7,3′-pyrrolidine-4′,3″-indoline]-2,2″,8(3*H*)-trione (6b). Yield 101 mg (43%); *R_f_* = 0.38; white solid; mp: 205–207 °C. IR (KBr): ν 3287 (NH), 3070 (ArH), 2972, 2939, 2876 (Alk), 1717, 1655, 1613 (C=O, C=N) cm^−1^. ^1^H NMR (500 MHz, DMSO-*d_6_*): δ 0.89 (t, *J* = 7.1 Hz, 3H, CH_3_), 1.09 (t, *J* = 7.0 Hz, 3H, CH_3_), 2.46 (s, 3H, 1′-NCH_3_), 2.85–2.90 (m, 1H, NCH_2_), 3.07–3.14 (m, 6H, 1′′-NCH_3_, CH_2_, NCH_2_), 3.43–3.50 (m, 3H, CH_2_, NCH_2_), 3.63 (d, *J* = 10.5 Hz, 1H, CH_2_), 4.60 (dd, *J* = 5.5, 1.9 Hz, 1H, 3a-H), 5.44 (d, *J* = 5.5 Hz, 1H, 9a-H), 6.98–7.04 (m, 2H, 5′′-H, 7′′-H), 7.18 (d, *J* = 7.5 Hz, 1H, 4′′-H), 7.33 (t, *J* = 7.7 Hz, 1H, 6′′-H), 7.43 (d, *J* = 1.9 Hz, 1H, 4-NH). ^13^C NMR (150 MHz, DMSO-*d_6_*): δ 13.69, 13.87 (2CH_3_), 27.27 (1′′-NCH_3_), 35.83, 38.61 (1,3-NCH_2_), 43.07 (1′-NCH_3_), 61.56, 62.41, 63.01, 63.88, 64.67, 66.15 (C-2′, C-3a, C-3′, C-3′′, C-5′, C-9a), 110.05 (C-7′′), 123.80, 124.91, 125.92, 130.68 (C-3a′′, C-4′′, C-5′′, C-6′′), 135.19 (5a-C=N), 145.64 (C-7a′′), 159.11 (2-C=O), 173.22 (8-C=O), 177.52 (2′′-C=O). HRMS (ESI): Calculated for C_22_H_27_N_7_O_3_S [M + H]^+^: 470.1967, Found: 470.1969.

(3aR*,4′R*,7S*,9aR*)-1,1′,1″,3-Tetramethyl-2-thioxo-1,2,3,3a,4,9a-hexahydro-8*H*-dispiro[imidazo[4,5-*e*]thiazolo[2,3-*c*][1,2,4]triazine-7,3′-pyrrolidine-4′,3″-indoline]-2″,8-dione (6c). Yield 84 mg (37%); *R_f_* = 0.41; white solid; mp: 228–231 °C. IR (KBr): ν 3436 (NH), 3027 (ArH), 2969, 2945, 2916, 2798 (Alk), 1727, 1703, 1653, 1612 (C=O, C=N) cm^−1^. ^1^H NMR (300 MHz, DMSO-*d_6_*): δ 2.47 (s, 3H, CH_3_), 2.78 (s, 3H, CH_3_), 3.10–3.17 (m, 7H, 2CH_3_, CH_2_), 3.45–3.52 (m, 2H, CH_2_), 3.66 (d, *J* = 10.4 Hz, 1H, CH_2_), 4.85 (d, *J* = 5.9 Hz, 1H, 3a-H), 5.57 (d, *J* = 5.6 Hz, 1H, 9a-H), 7.02–7.08 (m, 2H, 5′′-H, 7′′-H), 7.19 (d, *J* = 7.2 Hz, 1H, 4′′-H), 7.33 (t, *J* = 7.5 Hz, 1H, 6′′-H), 7.66 (s, 1H, 4-NH). ^13^C NMR (75 MHz, DMSO-*d_6_*): δ 26.07 (1′′-NCH_3_), 31.46, 35.53 (1,3-NCH_3_), 41.90 (1′-NCH_3_), 60.19, 61.76, 62.57, 65.01 (C-2′, C-3′,C-3′′, C-5′), 66.71, 67.53 (C-3a), C-9a), 108.95 (C-7′′), 122.93, 123.55, 124.69, 129.47 (C-3a′′, C-4′′, C-5′′, C-6′′), 134.68 (5a-C=N), 144.34 (C-7a′′), 171.65 (8-C=O), 176.20 (2′′-C=O), 184.33 (2-C=S). HRMS (ESI): Calculated for C_20_H_23_N_7_O_2_S_2_ [M + H]^+^: 458.1427, Found: 458.1427.

(3aS*,3′R*,3′′S*,9aS*)-1,3-Diethyl-1′,1′′-dimethyl-2-thioxo-1,3,3a,4,9,9a-hexahydrodispiro[imidazo[4,5-*e*]thiazolo[2,3-*c*][1,2,4]triazine-7,3′-pyrrolidine-4′,3′′-indole]-2′′,8(1′′*H*)-dione (6d). Yield 102 mg (42%); *R_f_* = 0.44; white solid; mp: 225–227 °C. IR (KBr): ν 3327 (NH), 3069, 3055 (ArH), 2967, 2936, 2884, 2829, 2795 (Alk), 1707, 1640, 1612 (C=O, C=N) cm^−1^. ^1^H NMR (300 MHz, DMSO-*d_6_*): δ 0.96 (t, *J* = 7.0 Hz, 3H, CH_3_), 1.13 (t, *J* = 7.0 Hz, 3H, CH_3_), 2.46 (s, 3H, 1′-NCH_3_), 3.08–3.26 (m, 6H, 1′′-NCH_3_, NCH_2_, CH_2_), 3.42–3.51 (m, 2H, CH_2_), 3.61–3.66 (m, 2H, CH_2_, NCH_2_), 3.93–3.99 (m, 1H, NCH_2_), 4.85 (d, *J* = 5.6 Hz, 1H, 3a-H), 5.61 (d, *J* = 5.4 Hz, 1H, 9a-H), 7.02–7.10 (m, 2H, 5′′-H, 7′′-H), 7.19 (d, *J* = 6.9 Hz, 1H, 4′′-H), 7.33 (t, *J* = 6.3 Hz, 1H, 6′′-H), 7.60 (d, J = 1.9 Hz, 1H, 4-NH). ^13^C NMR (75 MHz, DMSO-*d_6_*): δ 11.84, 11.91 (2CH_3_), 26.07 (1′′-NCH_3_), 38.13, 40.13 (1,3-NCH_2_), 41.77 (1′-NCH_3_), 60.26, 61.99, 62.75, 63.76, 64.97, 65.33 (C-2′, C-3′, C-3′′, C-3a, C-5′, C-9a), 108.88 (C-7′′), 122.95, 123.85, 124.74, 129.41 (C-3a′′, C-4′′, C-5′′, C-6′′), 134.28 (5a-C=N), 144.32 (C-7a′′), 171.68 (8-C=O), 176.24 (2′′-C=O), 182.04 (2-C=S). HRMS (ESI): Calculated for C_22_H_27_N_7_O_2_S_2_ [M + H]^+^: 486.1733, Found: 486.1740.

(3aR*,4′R*,7S*,9aS*)-1′-Cyclohexyl-1,1″,3-trimethyl-1,3a,4,9a-tetrahydro-8*H*-dispiro[imidazo[4,5-*e*]thiazolo[2,3-*c*][1,2,4]triazine-7,3′-pyrrolidine-4′,3″-indoline]-2,2″,8(3*H*)-trione (6e). Yield 117 mg (46%); *R_f_* = 0.54; white solid; mp: 244–245 °C. IR (KBr): ν 3435, 3306 (NH), 2931, 2854 (Alk), 1711, 1651, 1611, 1577 (C=O, C=N) cm^−1^. ^1^H NMR (300 MHz, DMSO-*d_6_*): δ 1.18–1.27 (m, 5H, Cy), 1.49–1.53 (m, 1H, Cy), 1.67–1.71 (m, 2H, Cy), 1.80–1.84 (m, 2H, Cy), 2.49 (s, 3H, NCH_3_), 2.57–2.60 (m, 1H, Cy), 2.92 (s, 3H, NCH_3_), 3.15 (s, 3H, 1′′-NCH_3_), 3.19 (d, *J* = 9.9 Hz, 1H, CH_2_), 3.50 (d, *J* = 10.0 Hz, 1H, CH_2_), 3.59 (d, *J* = 10.4 Hz, 1H, CH_2_), 3.68 (d, *J* = 10.3 Hz, 1H, CH_2_), 4.55 (d, *J* = 4.4 Hz, 1H, 3a-H), 5.38 (d, *J* = 5.7 Hz, 1H, 9a-H), 6.97–7.05 (m, 2H, 5′′-H, 7′′-H), 7.23 (d, *J* = 7.5 Hz, 1H, 4′′-H), 7.34 (t, *J* = 7.8 Hz, 1H, 6′′-H). 7.47 (s, 1H, 4-NH). ^13^C NMR (125 MHz, DMSO-*d_6_*): δ 23.99, 24.11, 25.74, 26.12, 27.96, 31.05, 31.52, 31.74 (3NCH_3_, Cy), 57.46, 59.21, 60.60, 60.65, 61.93, 63.88, 66.38 (C-2′, C-3′, C-3′′, C-5′, C-3a, C-9a, Cy), 108.95 (C-7′′), 122.52, 123.97, 124.84, 129.58 (C-3a′′, C-4′′, C-5′′, C-6′′), 134.42 (5a-C=N), 144.56 (C-7a′′), 159.38 (2-C=O), 171.88 (8-C=O), 176.29 (2′′-C=O). HRMS (ESI): Calculated for C_25_H_31_N_7_O_3_S [M + H]^+^: 510.2282, Found: 510.2277.

(3aR*,4′S*,7R*,9aS*)-1,1′,1″,3-Tetramethyl-1,3a,4,9a-tetrahydro-8*H*-dispiro[imidazo[4,5-*e*]thiazolo[2,3-*c*][1,2,4]triazine-7,3′-pyrrolidine-4′,3″-indoline]-2,2″,8(3*H*)-trione (7a). Method A: yield 79 mg (36%); method B: yield 35 mg (80%); *R_f_* = 0.58; white solid; mp: 264–266 °C. IR (KBr): ν 3357 (NH), 3065, 3025 (ArH), 2966, 2934, 2894, 2851 (Alk), 1736, 1717, 1704, 1646 (C=O, C=N) cm^−1^. ^1^H NMR (500 MHz, DMSO-*d_6_*): δ 2.49 (s, 3H, NCH_3_), 2.50 (s, 3H, NCH_3_), 2.91 (s, 3H, NCH_3_), 3.10 (d, *J* = 10.2 Hz, 1H, CH_2_), 3.14 (s, 3H, 1′′-NCH_3_), 3.41 (d, *J* = 10.7 Hz, 1H, CH_2_), 3.45 (d, *J* = 10.3 Hz, 1H, CH_2_), 3.66 (d, *J* = 10.7 Hz, 1H, CH_2_), 4.59 (d, *J* = 5.6 Hz, 1H, 3a-H), 5.34 (d, *J* = 5.6 Hz, 1H, 9a-H), 7.03–7.06 (m, 2H, 5′′-H, 7′′-H), 7.16 (d, *J* = 7.4 Hz, 1H, 4′′-H), 7.36 (t, *J* = 7.7 Hz, 1H, 6′′-H), 7.45 (s, 1H, 4-NH). ^13^C NMR (125 MHz, DMSO-*d_6_*): δ 26.16, 27.68, 31.35 (1,1′′,3-NCH_3_), 42.13 (1′-NCH_3_), 60.44, 61.48, 63.19, 63.67, 65.24, 66.39 (C-2′, C-3a, C-3′, C-3′′, C-5′, C-9a), 109.09 (C-7′′), 122.85, 123.32, 123.93, 129.76 (C-3a′′, C-4′′, C-5′′, C-6′′), 135.13 (5a-C=N), 144.74 (C-7a′′), 158.86 (2-C=O), 172.09 (8-C=O), 176.36 (2′′-C=O). HRMS (ESI): Calculated for C_20_H_23_N_7_O_3_S [M + H]^+^: 442.1643, Found: 442.1656.

(3aR*,4′S*,7R*,9aS*)-1,3-Diethyl-1′,1″-dimethyl-1,3a,4,9a-tetrahydro-8*H*-dispiro[imidazo[4,5-*e*]thiazolo[2,3-*c*][1,2,4]triazine-7,3′-pyrrolidine-4′,3″-indoline]-2,2″,8(3*H*)-trione (7b). Yield 96 mg (41%); *R_f_* = 0.60; white solid; mp: 252–254 °C. IR (KBr): ν 3325 (NH), 3093, 3053 (ArH), 2988, 2974, 2966, 2936, 2899, 2865, 2837 (Alk), 1707, 1641, 1611 (C=O, C=N) cm^−1^. ^1^H NMR (500 MHz, DMSO-*d_6_*): δ 0.91 (t, *J* = 7.1 Hz, 3H, CH_3_), 1.13 (t, *J* = 7.1 Hz, 3H, CH_3_), 2.49 (s, 3H, 1′-NCH_3_), 2.91*–*2.95 (m, 1H, NCH_2_), 3.09–3.15 (m, 5H, 1′′-NCH_3_, CH_2_, NCH_2_), 3.21–3.27 (m, 1H, NCH_2_), 3.38 (d, *J* = 10.6 Hz, 1H, CH_2_), 3.45–3.51 (m, 2H, CH_2_, NCH_2_), 3.67 (d, *J* = 10.6 Hz, 1H, CH_2_), 4.68 (d, *J* = 6.6 Hz, 1H, 3a-H), 5.60 (d, *J* = 6.6 Hz, 1H, 9a-H), 7.04–7.07 (m, 2H, 5′′-H, 7′′-H), 7.17 (d, *J* = 7.5 Hz, 1H, 4′′-H), 7.35 (t, *J* = 7.5 Hz, 1H, 6′′-H), 7.43 (s, 1H, 4-NH). ^13^C NMR (75 MHz, DMSO-*d_6_*): δ 12.26, 13.61 (2CH_3_), 26.02 (1′′-NCH_3_), 34.46, 37.97 (1,3-NCH_2_), 41.96 (1′-NCH_3_), 60.30. 61.33, 62.80, 63.13, 63.33, 64.27 (C-2′, C-3a, C-3′, C-3′′, C-5′, C-9a), 108.91 (C-7′′), 122.72, 123.20, 123.85, 129.60 (C-3a′′, C-4′′, C-5′′, C-6′′), 134.71 (5a-C=N), 144.63 (C-7a′′), 157.75 (2-C=O), 171.73 (8-C=O), 176.25 (2′′-C=O). HRMS (ESI): Calculated for C_22_H_27_N_7_O_3_S [M + H]^+^: 470.1960, Found: 470.1969.

(3aR*,4′S*,7R*,9aR*)-1,1′,1″,3-Tetramethyl-2-thioxo-1,2,3,3a,4,9a-hexahydro-8*H*-dispiro[imidazo[4,5-*e*]thiazolo[2,3-*c*][1,2,4]triazine-7,3′-pyrrolidine-4′,3″-indoline]-2″,8-dione (7c). Yield 101 mg (42%); *R_f_* = 0.66; white solid; mp: 224–226 °C. IR (KBr): ν 3398 (NH), 3112, 3056 (ArH), 2969, 2930, 2838, 2785 (Alk), 1711, 1698, 1636, 1609 (C=O, C=N) cm^−1^. ^1^H NMR (300 MHz, DMSO-*d_6_*): δ 2.50 (s, 3H, 1′-NCH_3_), 2.81 (s, 3H, NCH_3_), 3.10–3.15 (m, 4H, CH_2_, NCH_3_), 3.22 (s, 3H, NCH_3_), 3.38–3.47 (m, 2H, CH_2_), 3.68 (d, *J* = 10.7 Hz, 1H, CH_2_), 4.98 (d, *J* = 6.7 Hz, 1H, 3a-H), 5.82 (d, *J* = 6.9 Hz, 1H, 9a-H), 7.03–7.08 (m, 2H, 5′′-H, 7′′-H), 7.17 (d, *J* = 7.7 Hz, 1H, 4′′-H), 7.37 (t, *J* = 7.7 Hz, 1H, 6′′-H), 7.66 (s, 1H, 4-NH). ^13^C NMR (150 MHz, DMSO-*d_6_*): δ 27.29 (1′′-NCH_3_), 32.17, 36.39 (1,3-NCH_3_), 43.21 (1′-NCH_3_), 61.54, 62.53, 64.38, 64.78, 68.95, 69.58 (C-2′, C-3′, C-3′′, C-3a, C-9a), 110.22 (C-7′′), 124.00, 124.34, 125.04, 130.92 (C-3a′′, C-4′′, C-5′′, C=6′′), 137.15 (5a-C=N), 145.86 (C-7a′′), 172.81 (8-C=O), 177.41 (2′′-C=O), 184.61 (2-C=S). HRMS (ESI): Calculated for C_20_H_23_N_7_O_2_S_2_ [M + H]^+^:458.1421, Found: 458.1427.

(3aR*,4′S*,7R*,9aR*)-1,3-Diethyl-1′,1″-dimethyl-2-thioxo-1,2,3,3a,4,9a-hexahydro-8*H*-dispiro[imidazo[4,5-*e*]thiazolo[2,3-*c*][1,2,4]triazine-7,3′-pyrrolidine-4′,3″-indoline]-2″,8-dione (7d). Yield 107 mg (44%); *R_f_* = 0.67; white solid; mp: 148–150 °C. IR (KBr): ν 3311 (NH), 3056 (ArH), 2971, 2935, 2867, 2849, 2796 (Alk), 1709, 1648, 1612 (C=O, C=N) cm^−1^. ^1^H NMR (300 MHz, DMSO-*d_6_*): δ 0.97 (t, *J* = 6.6 Hz, 3H, CH_3_), 1.19 (t, *J* = 6.9 Hz, 3H, CH_3_), 2.50 (s, 3H, 1′-NCH_3_), 3.09–3.15 (m, 4H, 1′′-NCH_3_, CH_2_), 3.19–3.29 (m, 1H, CH_2_), 3.37–3.61 (m, 4H, CH_2_, NCH_2_), 3.67 (d, *J* = 10.5 Hz, 1H, CH_2_), 3.97–4.04 (m, 1H, NCH_2_), 5.01 (d, *J* = 6.9 Hz, 1H, 3a-H), 5.85 (d, *J* = 6.9 Hz, 1H, 9a-H), 7.04–7.08 (m, 2H, 5′′-H, 7′′-H), 7.17 (d, *J* = 7.5 Hz, 1H, 4′′-H), 7.37 (t, *J* = 7.5 Hz, 1H, 6′′-H), 7.64 (d, *J* = 1.9 Hz, 1H, 4-NH). ^13^C NMR (75 MHz, DMSO-*d_6_*): δ 11.49, 13.08 (2CH_3_), 26.06 (1′′-NCH_3_), 37.80, 41.24, 41.97 (1,3-NCH_2_, 1′-NCH_3_), 60.30, 61.25, 63.21, 65.57, 66.34 (C-2′, C-3′, C-3′′, C-3a, C-5′, C-9a), 108.97 (C-7′′), 122.81, 123.08, 123.83, 129.67 (C-3a′′, C-4′′, C-5′′, C-6′′), 135.47 (5a-C=N), 144.63 (C-7a′′), 171.35 (8-C=O), 176.18 (2′′-C=O), 181.83 (2-C=S). HRMS (ESI): Calculated for C_22_H_27_N_7_O_2_S_2_ [M + H]^+^: 486.1737, Found: 486.1740.

(3aR*,4′S*,7R*,9aS*)-1′-Cyclohexyl-1,1″,3-trimethyl-1,3a,4,9a-tetrahydro-8*H*-dispiro[imidazo[4,5-*e*]thiazolo[2,3-*c*][1,2,4]triazine-7,3′-pyrrolidine-4′,3″-indoline]-2,2″,8(3*H*)-trione (7e). Yield 84 mg (33%); *R_f_* = 0.73; white solid; mp: 155–156 °C. IR (KBr): ν 3371 (NH), 2933, 2852 (Alk), 1729, 1699, 1639, 1612 (C=O, C=N) cm^−1^. ^1^H NMR (300 MHz, DMSO-*d_6_*): δ 1.19–1.27 (m, 5H, Cy), 1.50–1.54 (m, 1H, Cy), 1.67–1.71 (m, 2H, Cy), 1.79–1.83 (m, 2H, Cy), 2.52 (s, 3H, NCH_3_), 2.59–2.63 (m, 1H, Cy), 2.92 (s, 3H, NCH_3_), 3.16 (s, 3H, 1′′-NCH_3_), 3.20 (d, *J* = 10.5 Hz, 1H, CH_2_), 3.45–3.52 (m, 2H, CH_2_), 3.69 (d, *J* = 10.5 Hz, 1H, CH_2_), 3.68 (d, *J* = 10.3 Hz, 1H, CH_2_), 4.61 (d, *J* = 6.4 Hz, 1H, 3a-H), 5.55 (d, *J* = 6.4 Hz, 1H, 9a-H), 7.03–7.08 (m, 2H, 5′′-H, 7′′-H), 7.19 (d, *J* = 7.5 Hz, 1H, 4′′-H), 7.37 (t, *J* = 7.6 Hz, 1H, 6′′-H), 7.46 (s, 1H, 4-NH). ^13^C NMR (75 MHz, DMSO-*d_6_*): δ 23.90, 23.99, 25.65, 26.04, 27.57, 30.88, 31.24, 31.34 (3NCH_3_, Cy), 56.92, 59.26, 59.56, 60.26, 62.28, 65.13, 66.24 (C-2′, C-3′, C-3′′, C-5′, C-3a, C-9a, Cy), 108.91 (C-7′′), 122.68, 123.60, 123.97, 129.59 (C-3a′′, C-4′′, C-5′′, C-6′′), 135.15 (5a-C=N), 144.67 (C-7a′′), 158.76 (2-C=O), 171.81 (8-C=O), 176.13 (2′′-C=O). HRMS (ESI): Calculated for C_25_H_31_N_7_O_3_S [M + H]^+^: 510.2282, Found: 510.2284.

## 4. Conclusions

In the present study, the synthesis of two types of isomeric dispirocompounds based on imidazothiazolotriazine and pyrrolidineoxindole, differing in the linear and angular structure of imidazothiazolotriazine fragment, was demonstrated. This article has shown that the 1,3-dipolar cycloaddition of azomethine ylides generated in situ from paraformaldehyde and N-alkylglycine derivatives to corresponding oxindolylidene derivatives of imidazothiazolotriazine allows the formation of the desired spirooxindoles of both typs in good yields but low diastereoselectivity. Meanwhile, all individual diastereomers were isolated chromatographically. Hitherto unavailable angular dispiro[imidazo[4,5-*e*]thiazolo[2,3-*c*][1,2,4]triazine-7,3′-pyrrolidine-4′,3″-indolines] can also be prepared via rearrangement of linear dispiro[imidazo[4,5-*e*]thiazolo- [3,2-*b*][1,2,4]triazine-6,3′-pyrrolidine-4′,3″-indolines] upon treatment with KOH with high yield and selectivity.

## Figures and Tables

**Table 1:** Selected bond lengths (Å) and angles (°) for racemates **3a**, **4e** and **7c**.

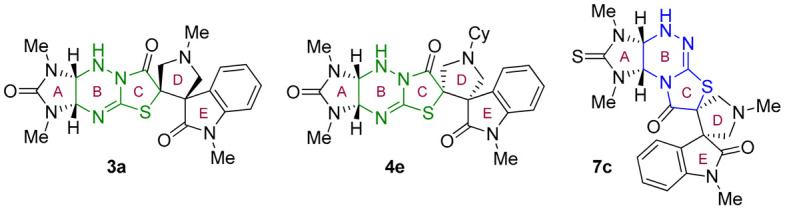			
Compound	3a	4e	7c
Bond lengths (Å)			
A			
C-N	1.3682(17)–1.4527(16)	1.356(4)–1.452(4)	1.3477(14)–1.4828(14)
C-C	1.5367(17)	1.520(5)	1.5313(14)
B			
N-N	1.4079(14)	1.420(4)	1.4103(13)
C-N	1.3905(16)–1.4664(16)	1.389(4)–1.475(5)	1.4046(13)–1.4832(12)
C=N	1.2601(16)	1.270(4)	1.2748(13)
C			
S-C	1.7557(12)–1.8187(12)	1.755(3)–1.827(3)	1.7637(10)–1.8254(10)
C-N	1.3641(15)	1.362(4)	1.3706(13)
C-C	1.5241(17)	1.525(5)	1.5297(14)
D			
C-N	1.4649(16)–1.4717(16)	1.452(5)–1.468(5)	1.4660(14)–1.4720(14)
C-C	1.5364(16)–1.5517(17)	1.545(5)–1.552(5)	1.5319(14)–1.5728(14)
E			
C-N	1.3637(16)–1.3993(17)	1.355(4)–1.410(4)	1.3632(13)–1.4097(13)
C-C	1.3994(17)–1.5446(17)	1.385(5)–1.547(5)	1.3999(14)–1.5505(14)
Dihedral angles between two spiro-jointed cycles (°)			
CD	89.1(1)	89.1(3)	89.0(1)
DE	89.5(1)	86.3(3)	88.3(1)
Torsion angles between two adjacent cycles (°)			
AB			
N-C-C-N	89.7(1), −150.9(1)	152.8(3), −87.3(3)	149.3(1), −92.8(1)
BC			
C-N-C-N	−173.6(1)	179.2(3)	157.9(1)
N-N-C-S (**3a**, **4e**) C-N-C-S (**7c**)	−176.4(1)	−179.5(2)	163.2(1)

## Data Availability

The data presented in this study are available in the article and in the Supplementary Materials.

## Supplementary Materials

The following supporting information can be downloaded at: https://www.mdpi.com/article/10.3390/ijms232213820/s1.
