# Supplementary material for: Synthesis, Structure and Stereochemistry of Dispirocompounds Based on Imidazothiazolotriazine and Pyrrolidineoxindole
Source: Int J Mol Sci. 2022 Nov 10;23(22):13820. doi: 10.3390/ijms232213820 (PMC9699425; doi:10.3390/ijms232213820)
Supplement: Supplementary file 1 [file ijms-23-13820-s001.zip › Supplementary Information.pdf]

Supplementary Information

Synthesis, Structure and Stereochemistry of Dispirocompounds Based on Imidazothiazolotriazine and Pyrrolidineoxindole

Alexei N. Izmest'ev [1], Valentina A. Karnoukhova [2], Alexander A. Larin [1], Angelina N. Kravchenko [1], Leonid L. Fershtat [1] and Galina A. Gazieva [1,*]

[1] N. D. Zelinsky Institute of Organic Chemistry, Russian Academy of Sciences, 47 Leninsky Prosp., Moscow 119991, Russia

[2] A. N. Nesmeyanov Institute of Organoelement Compounds, Russian Academy of Sciences, 28 Vavilova Street, Moscow 119991, Russia

* Correspondence: gaz@ioc.ac.ru

Table of Contents

1. Column chromatography S2
2. Crystallographic data for **3a**, **4e** and **7c** S3
3. Spectra ^{1}H and ^{13}C NMR of compounds **3a-e** and {^{1}H-^{1}H} NOESY NMR of **3a**,**b** S4
4. Spectra ^{1}H and ^{13}C NMR of compounds **4a-e** S16
5. Spectra ^{1}H and ^{13}C NMR of compounds **6a-e** and {^{1}H-^{1}H} NOESY NMR of **6a** S26
6. Spectra ^{1}H and ^{13}C NMR of compounds **7a-e** and {^{1}H-^{1}H} NOESY NMR of **7b** S37

Column chromatography

Column chromatography was carried out with silica gel (Acros Silica gel, for column chromatography, 60 Angstrom pore size, 0.040–0.063 mm) and to monitor the preparative separations, analytical thin layer chromatography (TLC) was performed at room temperature on pre-coated 0.25 mm thick silica gel 60 F_{254} aluminum plates 20 × 20 cm Merck, Darmstadt, Germany. Propanol-2 was used as eluent without preliminary purification.

Example of preparative separation of a mixture of diastereomers **3a** and **4a** using column chromatography. Chromatograms of the collected fractions of diastereomers **3a** (R_f = 0.21) and **4a** (R_f = 0.53).

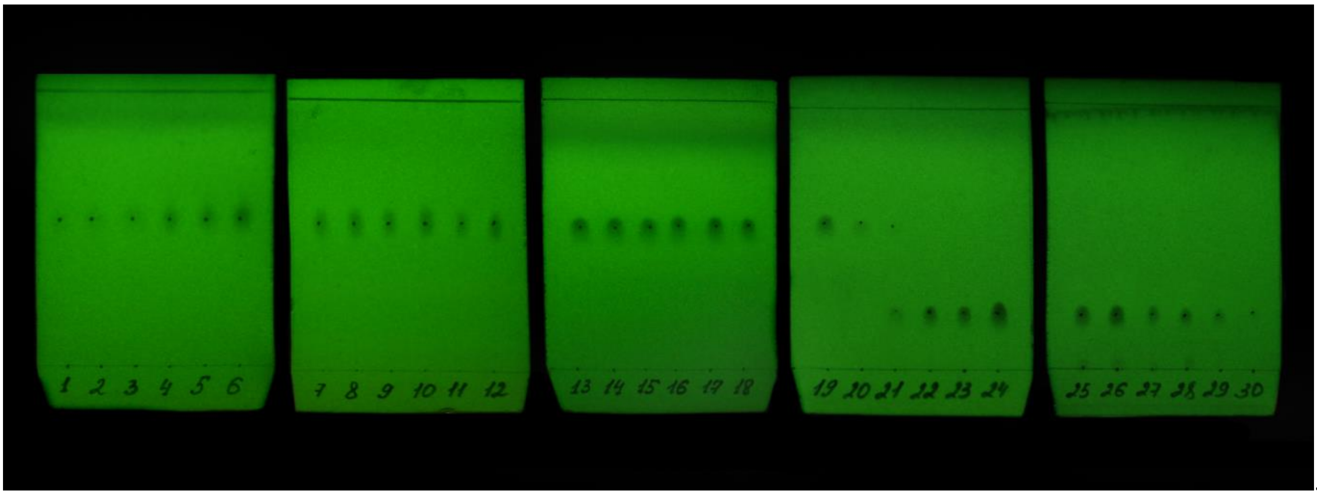

.

Table S1. Crystallographic data for **3a**, **4e** and **7c**

	**3a**	**4e**	**7c**
CCDC	2215817	2215818	2215819
Empirical formula	$C_{20}H_{23}N_7O_3S$	$C_{27}H_{39}N_7O_5S$	$C_{20}H_{23}N_7O_2S_2$
Formula weight	441.51	573.71	457.57
T (K)	120	295	120
Crystal system	Monoclinic	Orthorhombic	Triclinic
Space group	$P2_1/c$	$P2_12_12_1$	P-1
Z / Z'	4 / 1	4 / 1	2 / 1
a, Å	10.4431(7)	9.5788(2)	8.8678(4)
b, Å	15.6683(11)	16.7852(3)	9.6639(5)
c, Å	13.1324(9)	18.1337(3)	13.3198(5)
α, °	90	90	87.1340(10)
β, °	109.1969(14)	90	70.557(2)
γ, °	90	90	80.6250(10)
V, Å^3	2029.3(2)	2915.57(9)	1061.99(8)
d_{calc} (g cm^{-1})	1.445	1.307	1.431
μ, cm^{-1}	1.99	1.6	2.84
F(000)	928	1224	480
$2\theta_{max}$, °	61.4	61.1	61.2
Refls collected	27883	44223	14488
Independent refls [Rint]	6271 [0.0431]	8926 [0.0869]	6495 [0.0164]
Observed refls [$I > 2\sigma(I)$]	5148	4752	5733
Parameters	288	408	288
*R*1	0.0399	0.0562	0.0323
w*R*2	0.1075	0.1332	0.0860
GOF	1.035	1.015	1.022
Residual density, $\Delta\rho_{max}/\ \Delta\rho_{min}$ (e A^{-3})	0.525/-0.252	0.402/-0.211	0.466/-0.274

Table S2. Sum of CNN, CNC, NNH and CNH angles (°) for **3a**, **4e** and **7c**

	**3a**	**4e**	**7c**
N(1)	351.06	354.39	359.4
N(2)	354.39	351.93	358.31
N(3)	330.04	327.83	340.06
N(4)	334.77	333.51	337.69

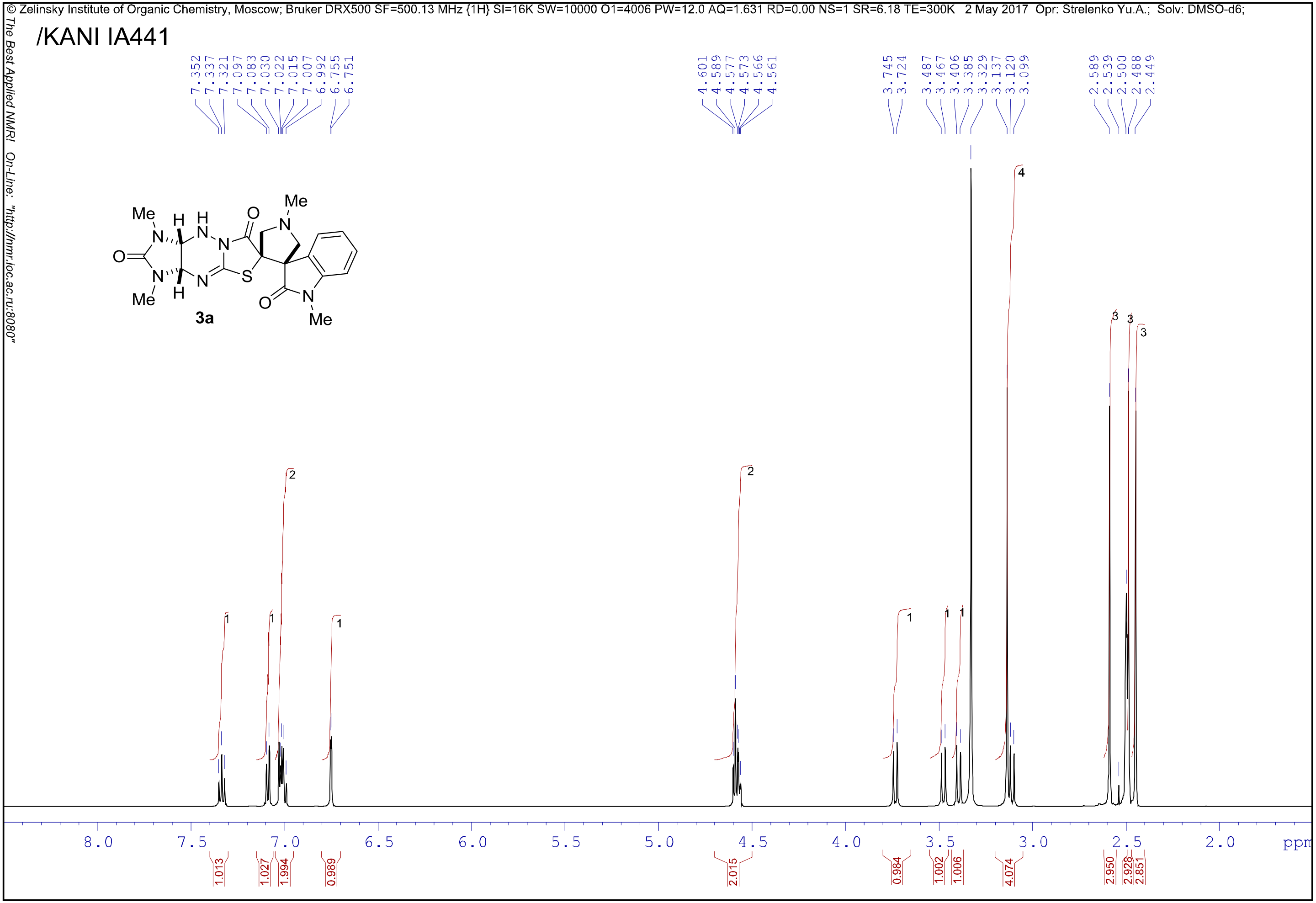
© Zelinsky Institute of Organic Chemistry, Moscow; Bruker DRX500 SF=500.13 MHz {1H} SI=16K SW=10000 O1=4006 PW=12.0 AQ=1.631 RD=0.00 NS=1 SR=6.18 TE=300K 2 May 2017 Opr: Strelenko Yu.A.; Solv: DMSO-d6;
/KANI IA441
The Best Applied NMR! On-Line: "http://nmr.ioc.ac.ru:8080"
7.352 7.337 7.321 7.097 7.083 7.030 7.022 7.015 7.007 6.992 6.755 6.751
4.601 4.589 4.577 4.573 4.566 4.561
3.745 3.724 3.487 3.467 3.406 3.385 3.329 3.137 3.120 3.099
2.589 2.539 2.500 2.488 2.449
Me
H
H
O
Me
N
N
N
N
O
N
S
N
N
Me
H
O
N
Me
3a
1.013 1.027 1.994 0.989 2.015 0.984 1.002 1.006 4.074 2.950 2.928 2.851
8.0 7.5 7.0 6.5 6.0 5.5 5.0 4.5 4.0 3.5 3.0 2.5 2.0 ppm

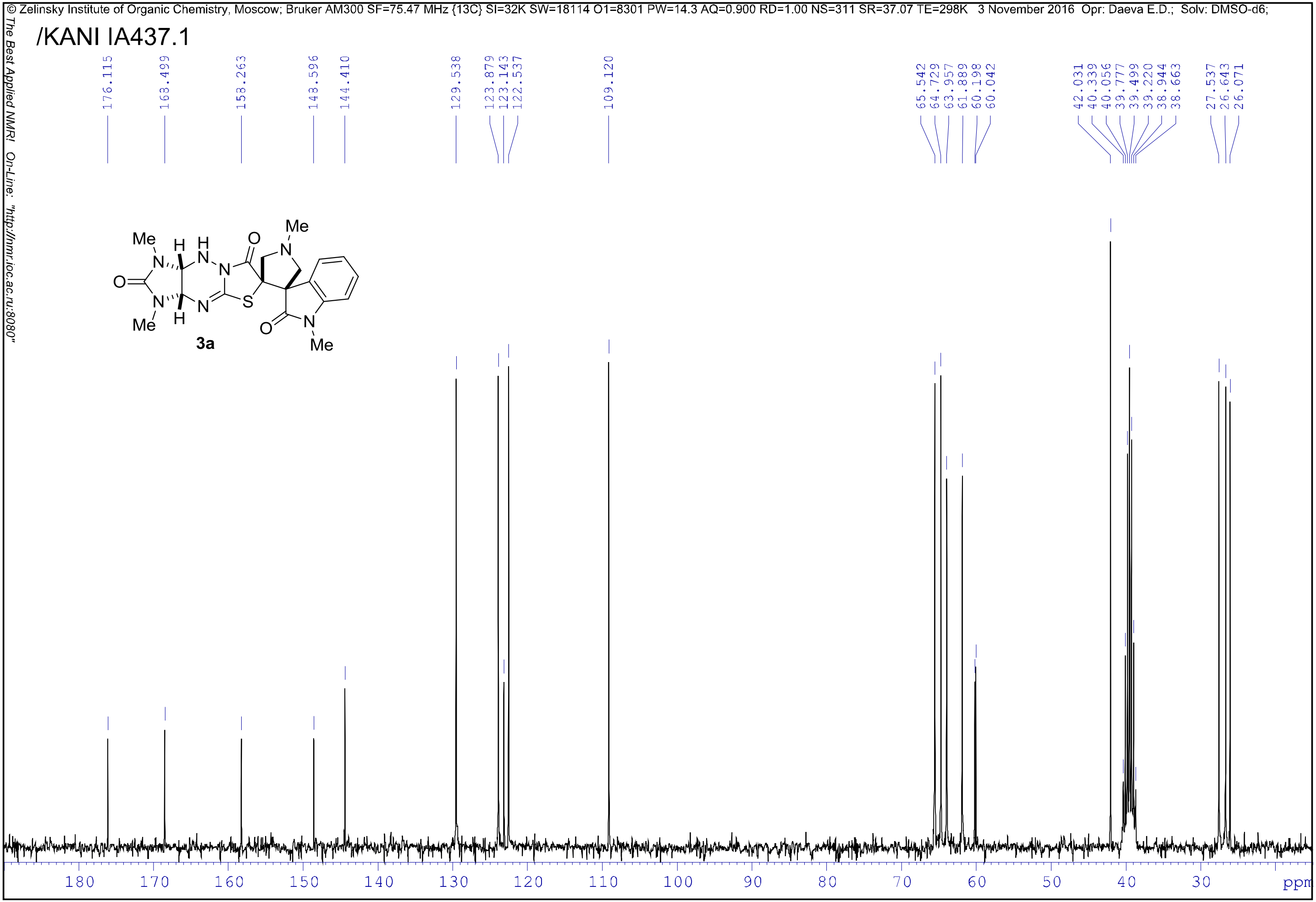
© Zelinsky Institute of Organic Chemistry, Moscow; Bruker AM300 SF=75.47 MHz {13C} SI=32K SW=18114 O1=8301 PW=14.3 AQ=0.900 RD=1.00 NS=311 SR=37.07 TE=298K 3 November 2016 Opr: Daeva E.D.; Solv: DMSO-d6;
/KANI IA437.1
The Best Applied NMR! On-Line: "http://nmr.ioc.ac.ru:8080"
176.115
163.499
158.263
148.596
144.410
129.538
123.879
123.143
122.537
109.120
65.542
64.729
63.957
61.889
60.198
60.042
42.031
40.339
40.056
39.777
39.499
39.220
38.944
38.663
27.537
26.643
26.071
3a
180 170 160 150 140 130 120 110 100 90 80 70 60 50 40 30 ppm

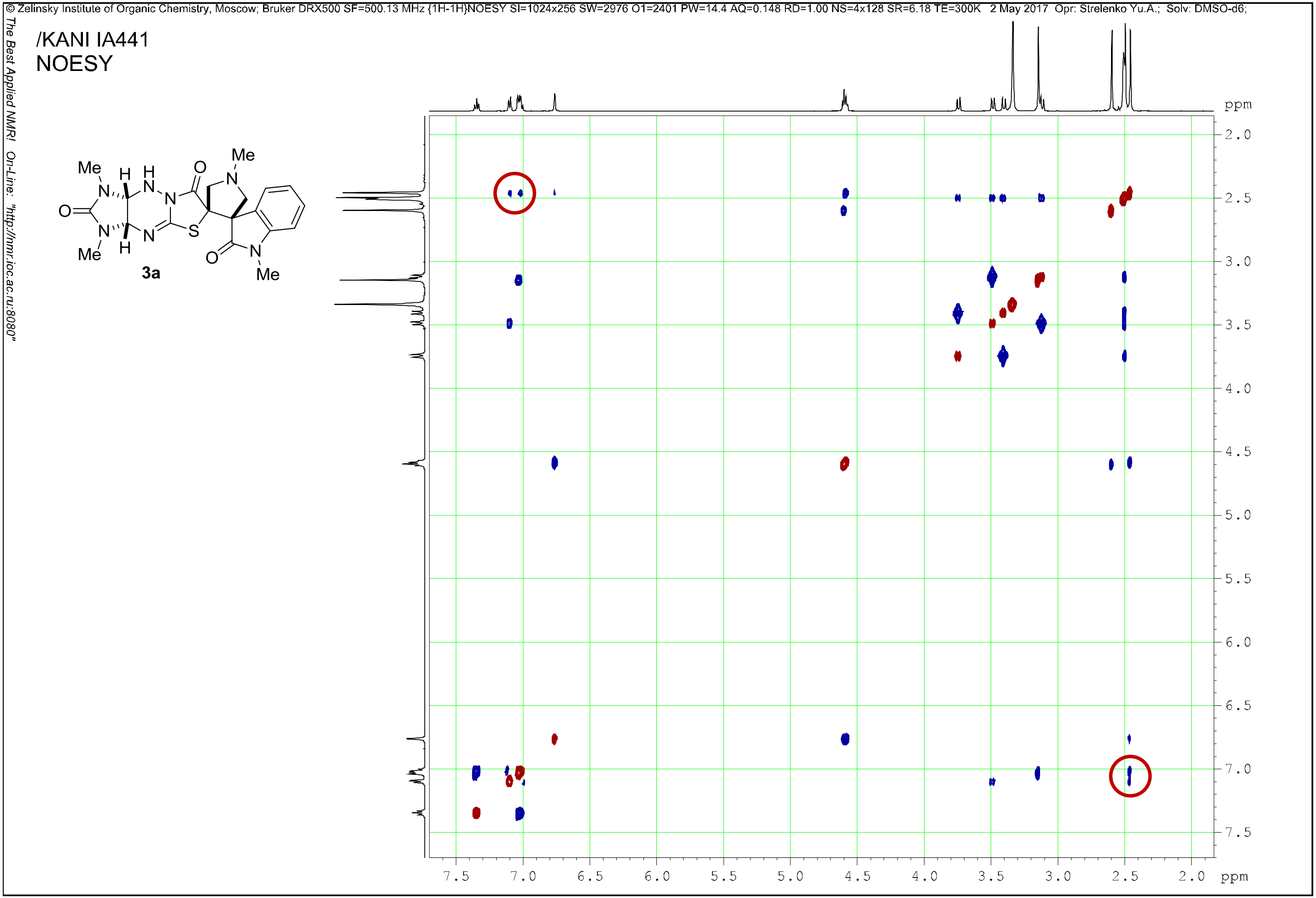
© Zelinsky Institute of Organic Chemistry, Moscow; Bruker DRX500 SF=500.13 MHz {1H-1H}NOESY SI=1024x256 SW=2976 O1=2401 PW=14.4 AQ=0.148 RD=1.00 NS=4x128 SR=6.18 TE=300K 2 May 2017 Opr: Strelenko Yu.A.; Solv: DMSO-d6;
The Best Applied NMR! On-Line: "http://nmr.ioc.ac.ru:8080"
/KANI IA441
NOESY
3a
ppm

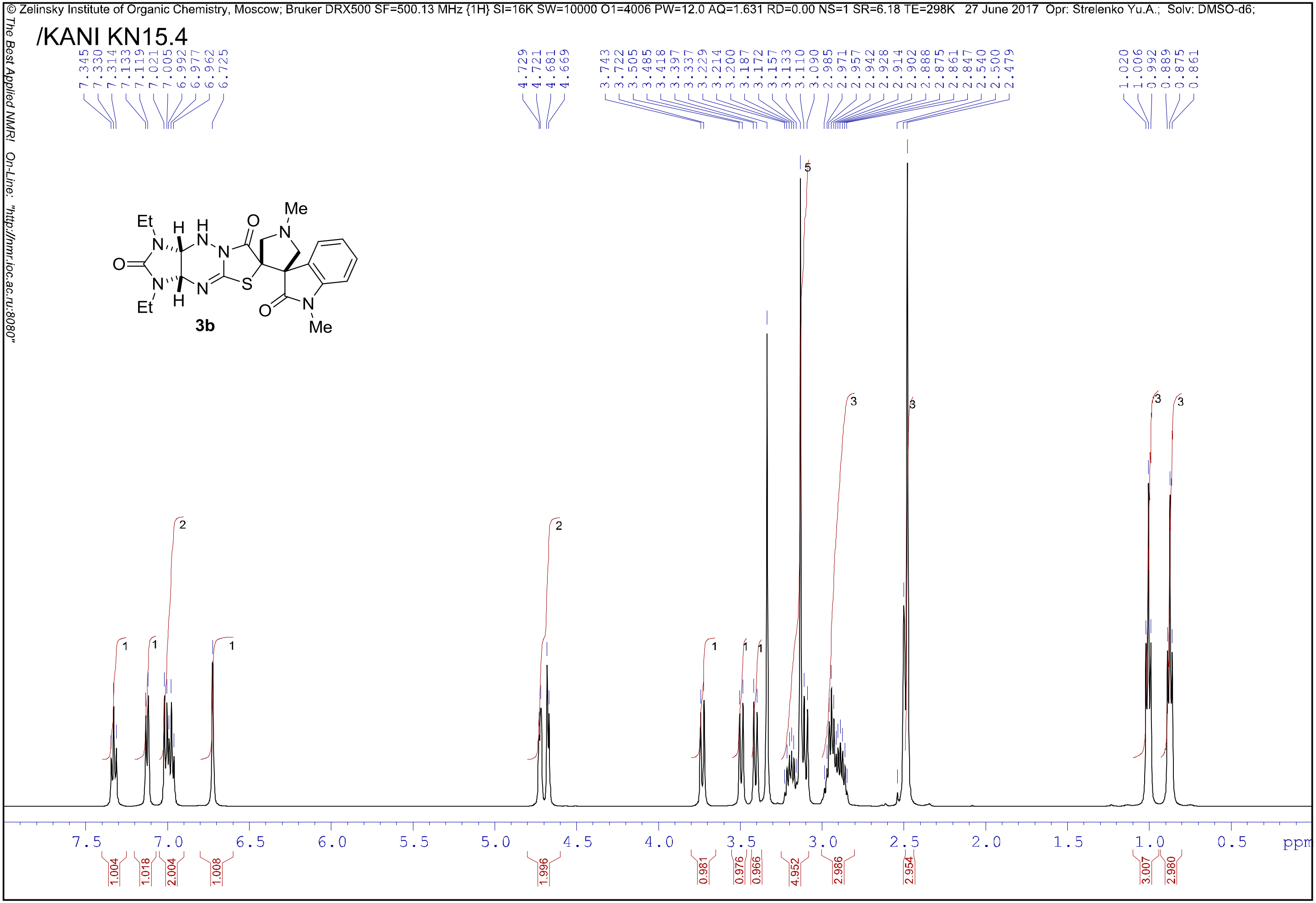
© Zelinsky Institute of Organic Chemistry, Moscow; Bruker DRX500 SF=500.13 MHz {1H} SI=16K SW=10000 O1=4006 PW=12.0 AQ=1.631 RD=0.00 NS=1 SR=6.18 TE=298K 27 June 2017 Opr: Strelenko Yu.A.; Solv: DMSO-d6;
The Best Applied NMR! On-Line: "http://nmr.ioc.ac.ru:8080"
/KANI KN15.4
7.345 7.330 7.314 7.133 7.119 7.021 7.005 6.992 6.977 6.962 6.725
4.729 4.721 4.681 4.669
3.743 3.722 3.505 3.485 3.418 3.397 3.337 3.229 3.214 3.200 3.187 3.172 3.157 3.133 3.110 3.090 2.985 2.971 2.957 2.942 2.928 2.914 2.902 2.888 2.875 2.861 2.847 2.540 2.500 2.479
1.020 1.006 0.992 0.889 0.875 0.861
3b
ppm
1.004 1.018 2.004 1.008 1.996 0.981 0.976 0.966 4.952 2.986 2.954 3.007 2.980

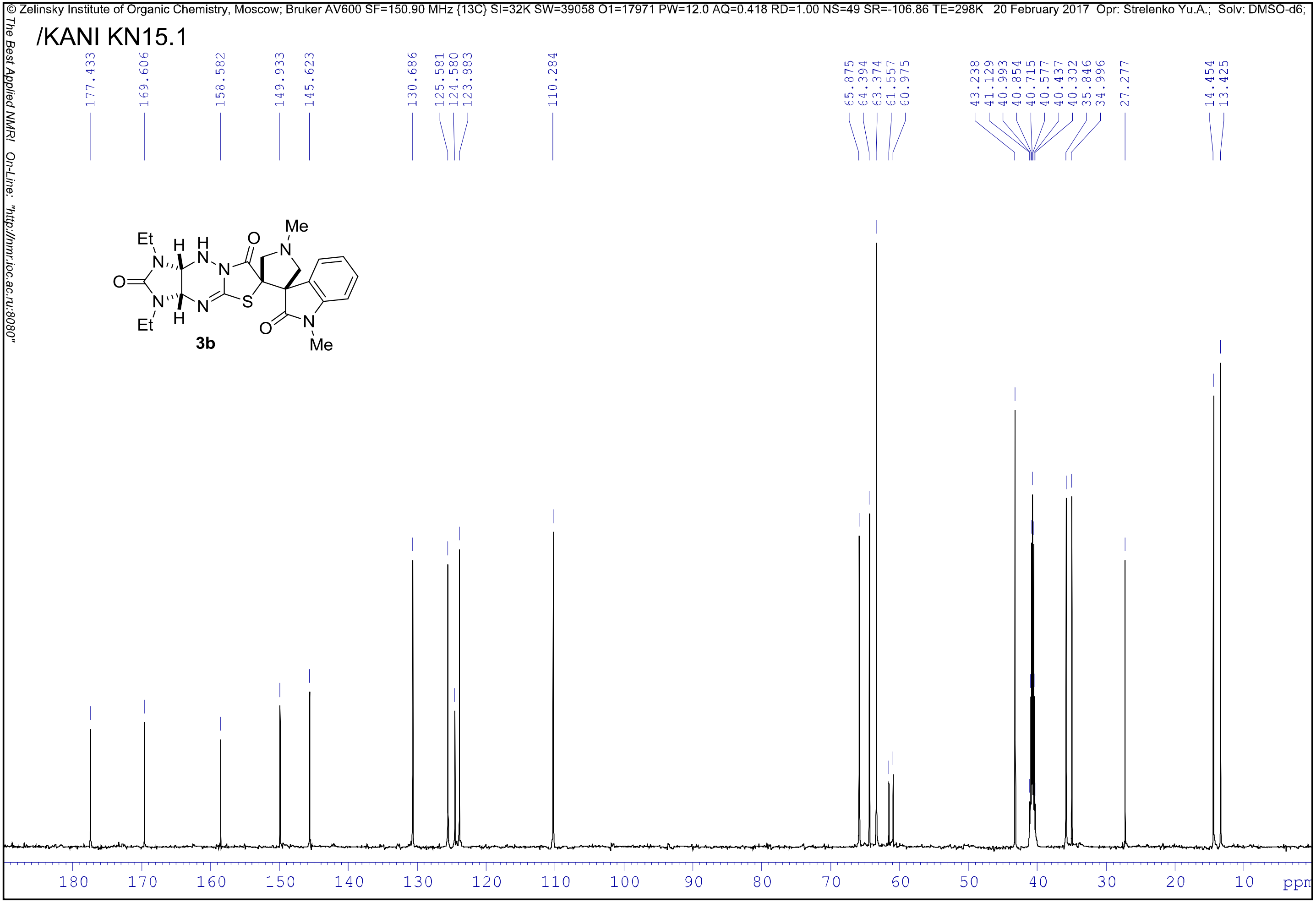
© Zelinsky Institute of Organic Chemistry, Moscow; Bruker AV600 SF=150.90 MHz {13C} SI=32K SW=39058 O1=17971 PW=12.0 AQ=0.418 RD=1.00 NS=49 SR=-106.86 TE=298K 20 February 2017 Opr: Strelenko Yu.A.; Solv: DMSO-d6;
The Best Applied NMR! On-Line: "http://nmr.ioc.ac.ru:8080"
/KANI KN15.1
177.433
169.606
158.582
149.933
145.623
130.686
125.581
124.580
123.883
110.284
65.875
64.394
63.374
61.557
60.975
43.238
41.129
40.993
40.854
40.715
40.577
40.437
40.302
35.846
34.996
27.277
14.454
13.425
Et
H
H
N
O
Me
N
N
N
O
N
N
S
N
H
Et
O
N
Me
3b
180
170
160
150
140
130
120
110
100
90
80
70
60
50
40
30
20
10
ppm

© Zelinsky Institute of Organic Chemistry, Moscow; Bruker DRX500 SF=500.13 MHz {1H-1H}NOESY SI=1024x512 SW=4223 O1=2055 PW=14.4 AQ=0.104 RD=1.00 NS=4x256 SR=6.18 TE=298K 27 June 2017 Opr: Strelenko Yu.A.; Solv: DMSO-d6;

The Best Applied NMR! On-Line: "http://nmr.ioc.ac.ru:8080"

/KANI KN15.4
NOESY

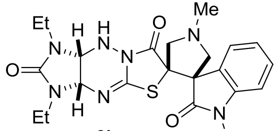

3b

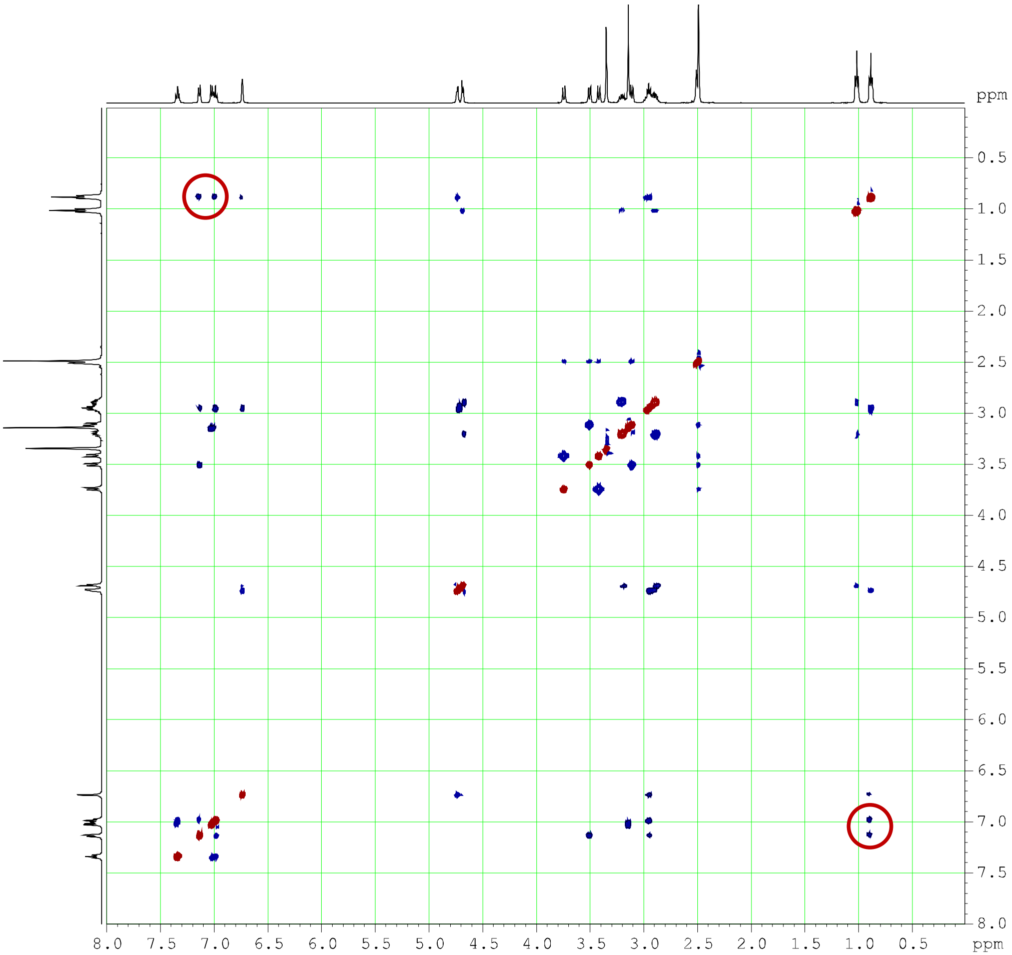

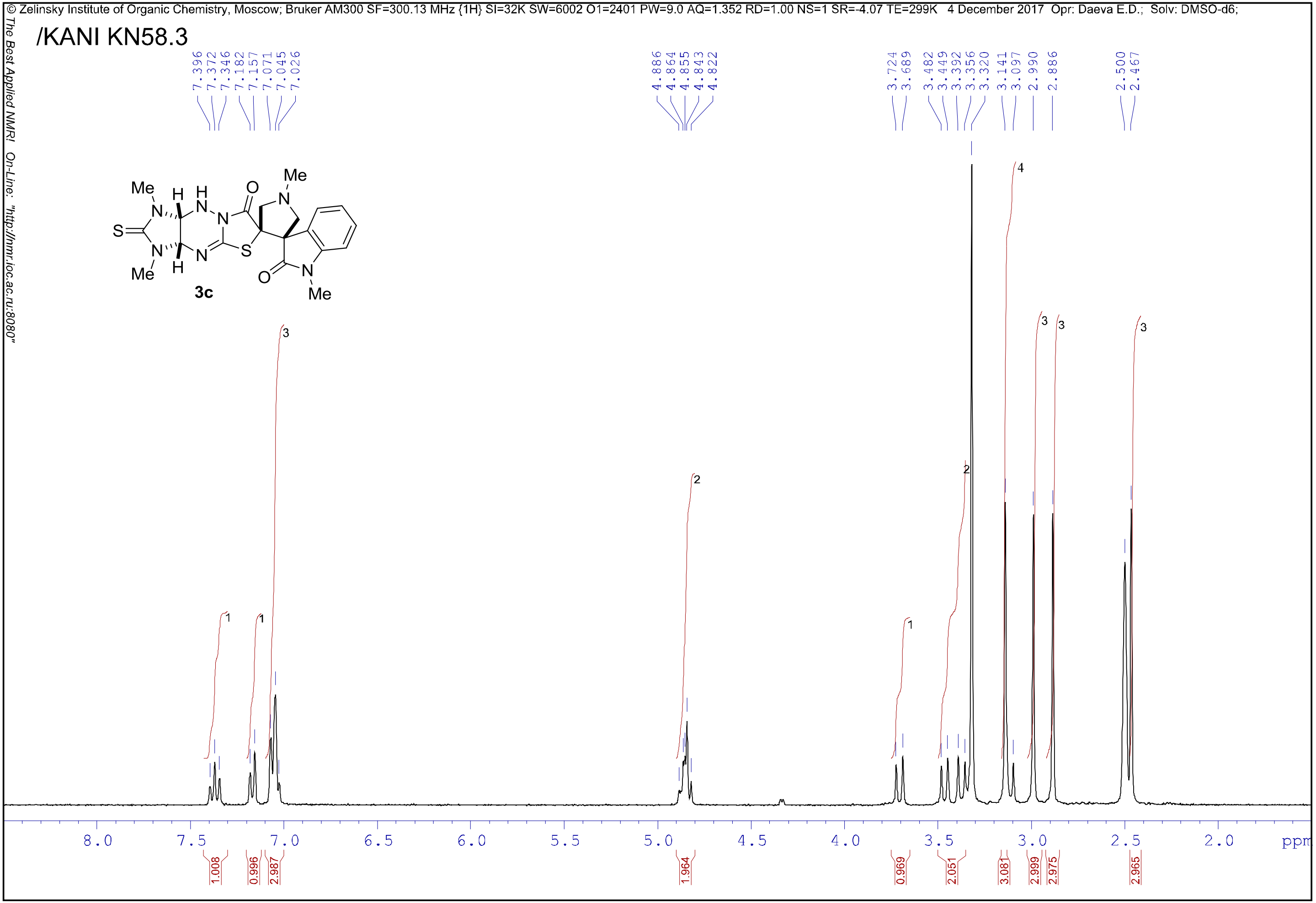

© Zelinsky Institute of Organic Chemistry, Moscow; Bruker AM300 SF=300.13 MHz {1H} SI=32K SW=6002 O1=2401 PW=9.0 AQ=1.352 RD=1.00 NS=1 SR=-4.07 TE=299K 4 December 2017 Opr: Daeva E.D.; Solv: DMSO-d6;
The Best Applied NMR! On-Line: "http://nmr.ioc.ac.ru:8080"
/KANI KN58.3
7.396 7.372 7.346 7.182 7.157 7.071 7.045 7.026
4.886 4.864 4.855 4.843 4.822
3.724 3.689 3.482 3.449 3.392 3.356 3.320 3.141 3.097 2.990 2.886
2.500 2.467
3c
8.0 7.5 7.0 6.5 6.0 5.5 5.0 4.5 4.0 3.5 3.0 2.5 2.0 ppm
1.008 0.996 2.987 1.964 0.969 2.051 3.081 2.999 2.975 2.965

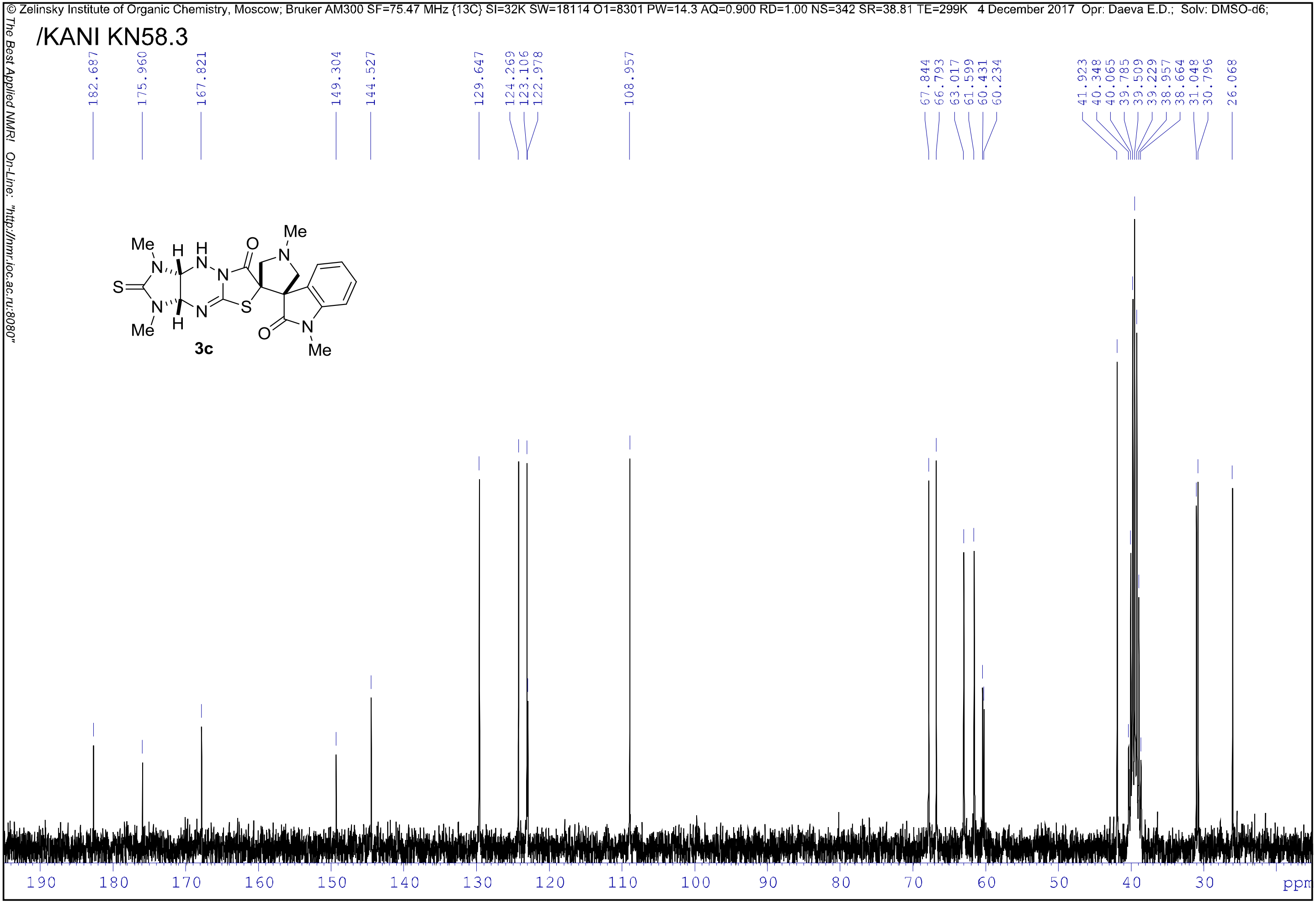
© Zelinsky Institute of Organic Chemistry, Moscow; Bruker AM300 SF=75.47 MHz {13C} SI=32K SW=18114 O1=8301 PW=14.3 AQ=0.900 RD=1.00 NS=342 SR=38.81 TE=299K 4 December 2017 Opr: Daeva E.D.; Solv: DMSO-d6;
/KANI KN58.3
The Best Applied NMR! On-Line: "http://nmr.ioc.ac.ru:8080"
182.687
175.960
167.821
149.304
144.527
129.647
124.269
123.106
122.978
108.957
67.844
66.793
63.017
61.599
60.431
60.234
41.923
40.348
40.065
39.785
39.509
39.229
38.957
38.664
31.048
30.796
26.068
Me
H
H
O
Me
N
N
N
N
S
N
N
S
N
O
N
Me
H
Me
3c
190 180 170 160 150 140 130 120 110 100 90 80 70 60 50 40 30 ppm

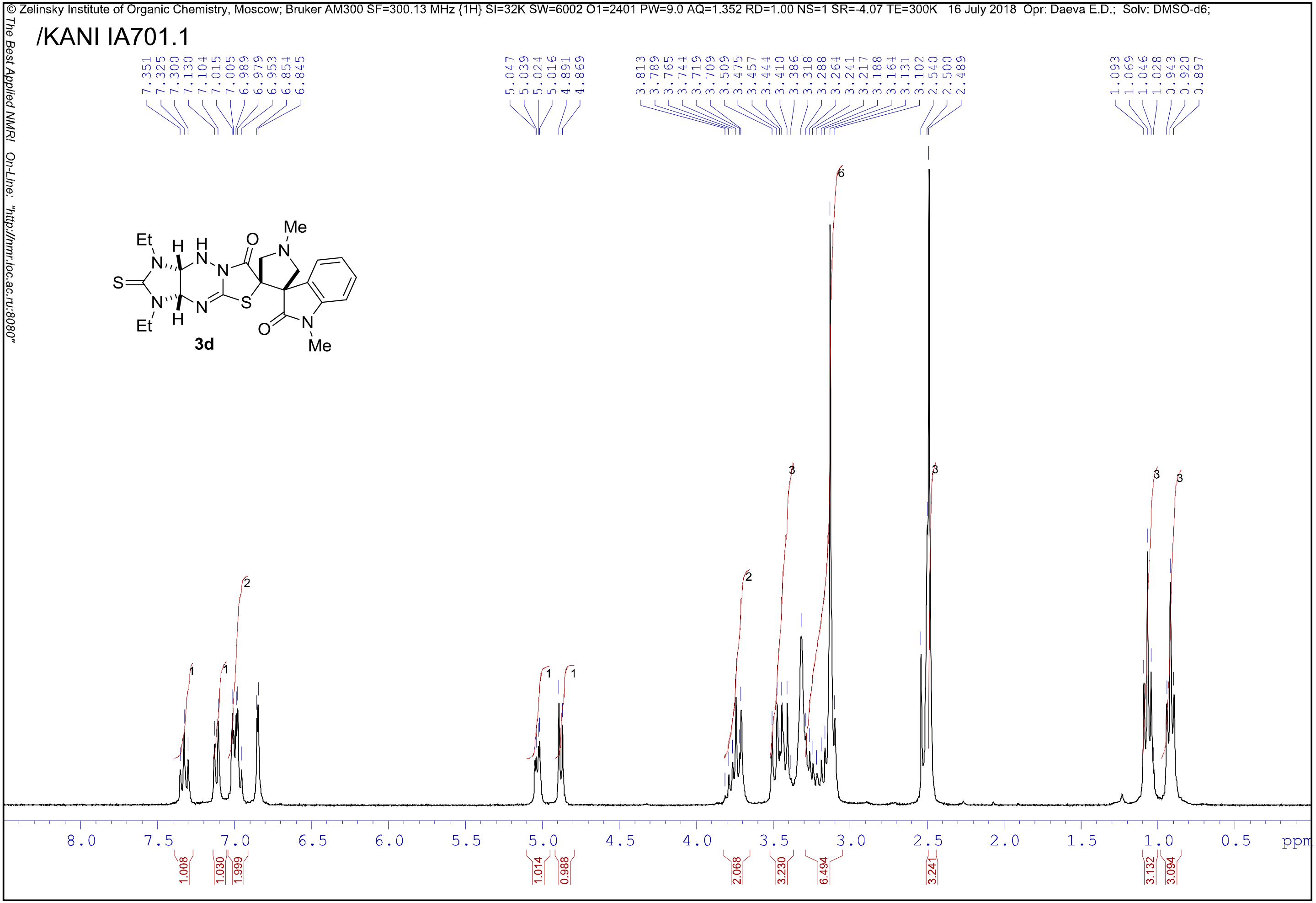
© Zelinsky Institute of Organic Chemistry, Moscow; Bruker AM300 SF=300.13 MHz {1H} SI=32K SW=6002 O1=2401 PW=9.0 AQ=1.352 RD=1.00 NS=1 SR=-4.07 TE=300K 16 July 2018 Opr: Daeva E.D.; Solv: DMSO-d6;
/KANI IA701.1
The Best Applied NMR! On-Line: "http://nmr.ioc.ac.ru:8080"
7.351 7.325 7.300 7.130 7.104 7.015 7.005 6.989 6.979 6.953 6.854 6.845
5.047 5.039 5.024 5.016 4.891 4.869
3.813 3.789 3.765 3.744 3.719 3.709 3.509 3.475 3.457 3.444 3.410 3.386 3.318 3.288 3.264 3.241 3.217 3.188 3.164 3.131 3.102 2.540 2.500 2.489
1.093 1.069 1.046 1.028 0.943 0.920 0.897
Et
H
H
N
O
Me
S
Et
3d
Me
8.0 7.5 7.0 6.5 6.0 5.5 5.0 4.5 4.0 3.5 3.0 2.5 2.0 1.5 1.0 0.5 ppm
1.008 1.030 1.999 1.014 0.988 2.068 3.230 6.494 3.241 3.132 3.094

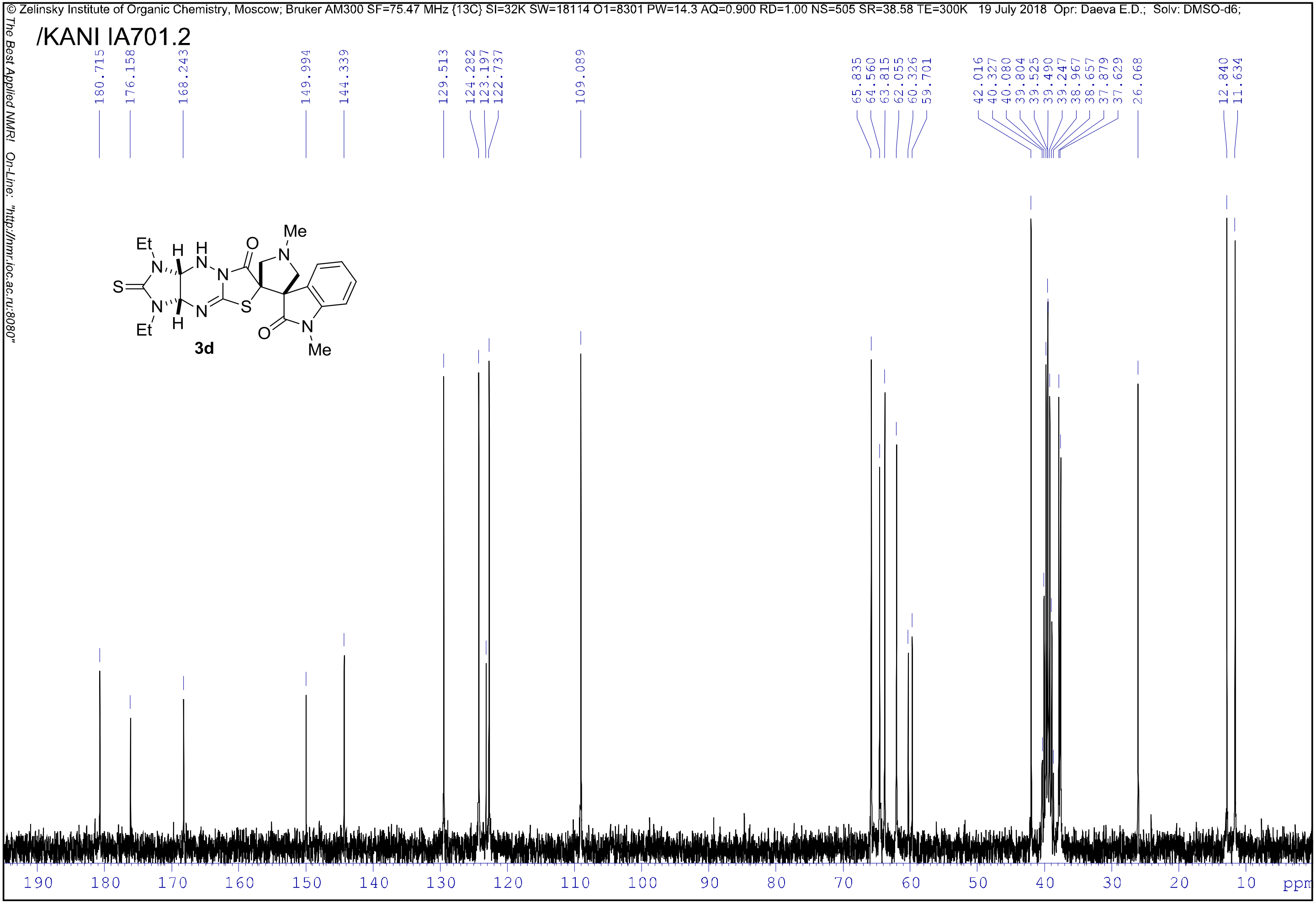
© Zelinsky Institute of Organic Chemistry, Moscow; Bruker AM300 SF=75.47 MHz {13C} SI=32K SW=18114 O1=8301 PW=14.3 AQ=0.900 RD=1.00 NS=505 SR=38.58 TE=300K 19 July 2018 Opr: Daeva E.D.; Solv: DMSO-d6;
The Best Applied NMR! On-Line: "http://nmr.ioc.ac.ru:8080"
/KANI IA701.2
180.715
176.158
168.243
149.994
144.339
129.513
124.282
123.197
122.737
109.089
65.835
64.560
63.815
62.055
60.326
59.701
42.016
40.327
40.080
39.804
39.525
39.490
39.247
38.967
38.657
37.879
37.629
26.068
12.840
11.634
3d
190 180 170 160 150 140 130 120 110 100 90 80 70 60 50 40 30 20 10 ppm

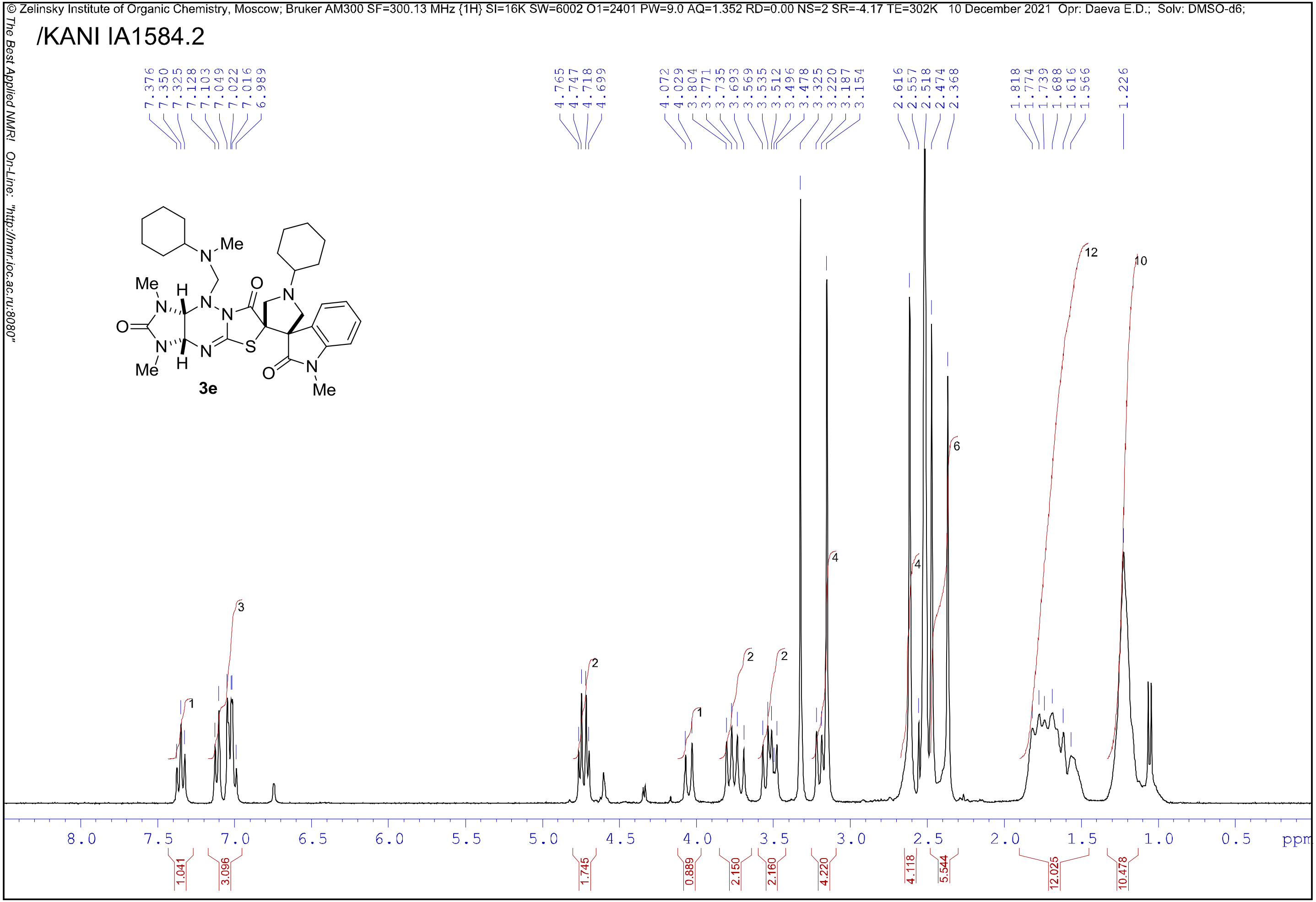
© Zelinsky Institute of Organic Chemistry, Moscow; Bruker AM300 SF=300.13 MHz {1H} SI=16K SW=6002 O1=2401 PW=9.0 AQ=1.352 RD=0.00 NS=2 SR=-4.17 TE=302K 10 December 2021 Opr: Daeva E.D.; Solv: DMSO-d6;
The Best Applied NMR! On-Line: "http://nmr.ioc.ac.ru:8080"
/KANI IA1584.2
7.376 7.350 7.325 7.128 7.103 7.049 7.022 7.016 6.989
4.765 4.747 4.718 4.699
4.072 4.029 3.804 3.771 3.735 3.693 3.569 3.535 3.512 3.496 3.478 3.325 3.220 3.187 3.154
2.616 2.557 2.518 2.474 2.368
1.818 1.774 1.739 1.688 1.616 1.566
1.226
Me
N
H
O
S
3e
1 3 2 1 2 2 4 4 6 12 10
8.0 7.5 7.0 6.5 6.0 5.5 5.0 4.5 4.0 3.5 3.0 2.5 2.0 1.5 1.0 0.5 ppm
1.041 3.096 1.745 0.889 2.150 2.160 4.220 4.118 5.544 12.025 10.478

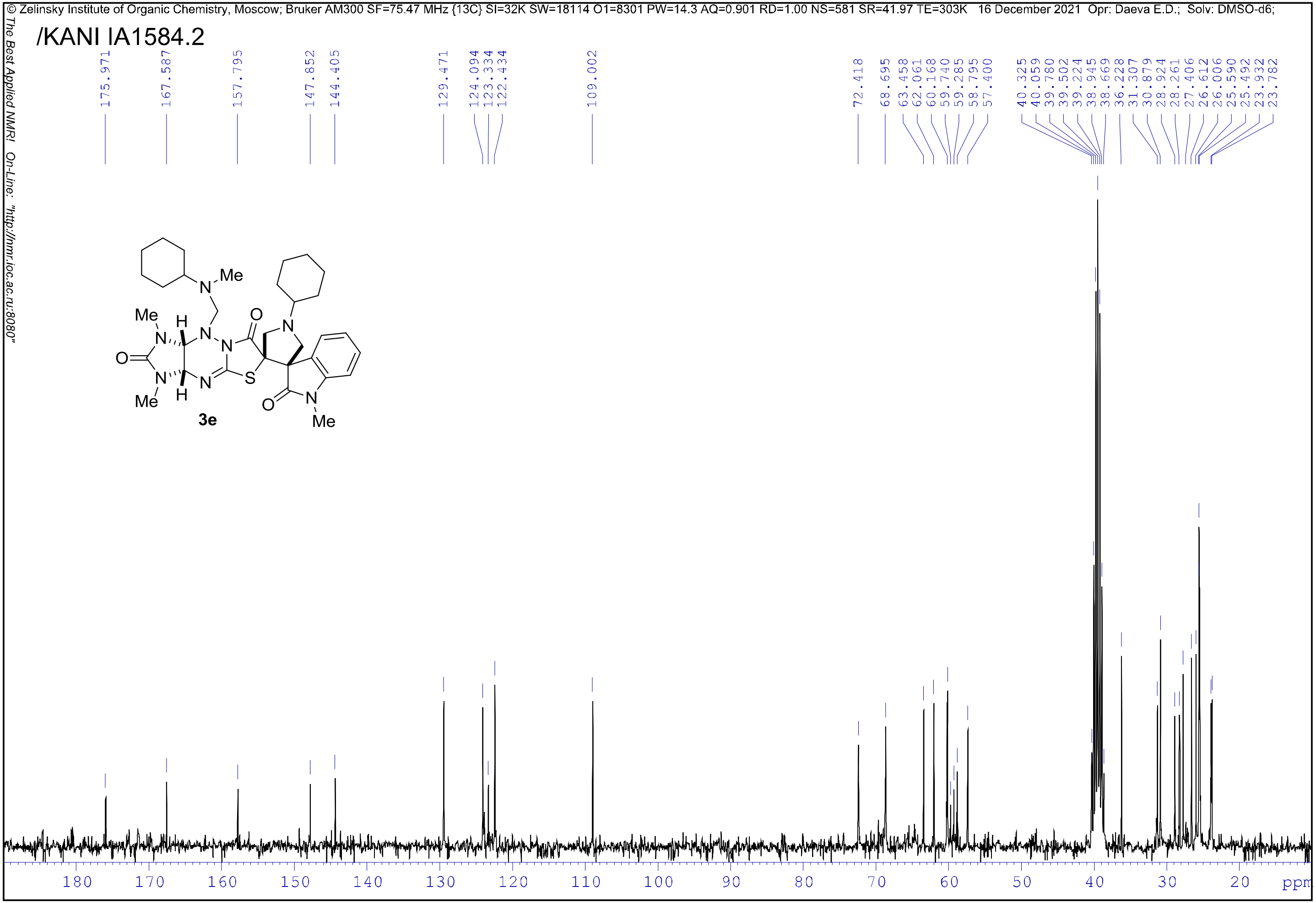
© Zelinsky Institute of Organic Chemistry, Moscow; Bruker AM300 SF=75.47 MHz {13C} SI=32K SW=18114 O1=8301 PW=14.3 AQ=0.901 RD=1.00 NS=581 SR=41.97 TE=303K 16 December 2021 Opr: Daeva E.D.; Solv: DMSO-d6;
/KANI IA1584.2
The Best Applied NMR! On-Line: "http://nmr.ioc.ac.ru:8080"
175.971
167.587
157.795
147.852
144.405
129.471
124.094
123.334
122.434
109.002
72.418
68.695
63.458
62.061
60.168
59.740
59.285
58.795
57.400
40.325
40.059
39.780
39.502
39.224
38.945
38.669
36.228
31.307
30.879
28.924
28.261
27.406
26.612
26.006
25.590
25.492
23.932
23.782
3e
180 170 160 150 140 130 120 110 100 90 80 70 60 50 40 30 20 ppm

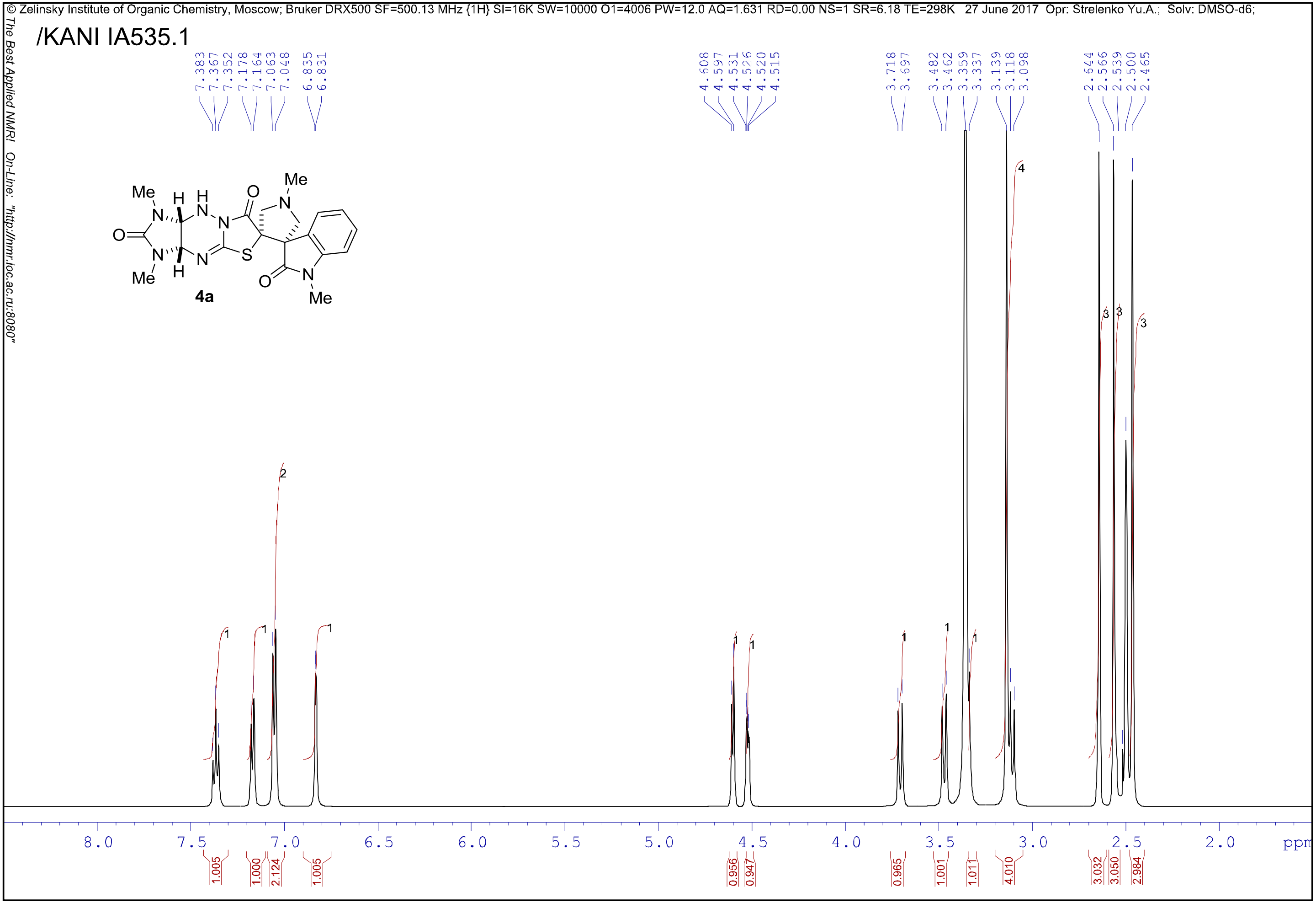
© Zelinsky Institute of Organic Chemistry, Moscow; Bruker DRX500 SF=500.13 MHz {1H} SI=16K SW=10000 O1=4006 PW=12.0 AQ=1.631 RD=0.00 NS=1 SR=6.18 TE=298K 27 June 2017 Opr: Strelenko Yu.A.; Solv: DMSO-d6;
The Best Applied NMR! On-Line: "http://nmr.ioc.ac.ru:8080"
/KANI IA535.1
7.383 7.367 7.352 7.178 7.164 7.063 7.048 6.835 6.831
4.608 4.597 4.531 4.526 4.520 4.515
3.718 3.697 3.482 3.462 3.359 3.337 3.139 3.118 3.098
2.644 2.566 2.539 2.500 2.465
4a
8.0 7.5 7.0 6.5 6.0 5.5 5.0 4.5 4.0 3.5 3.0 2.5 2.0 ppm
1.005 1.000 2.124 1.005 0.956 0.947 0.965 1.001 1.011 4.010 3.032 3.050 2.984

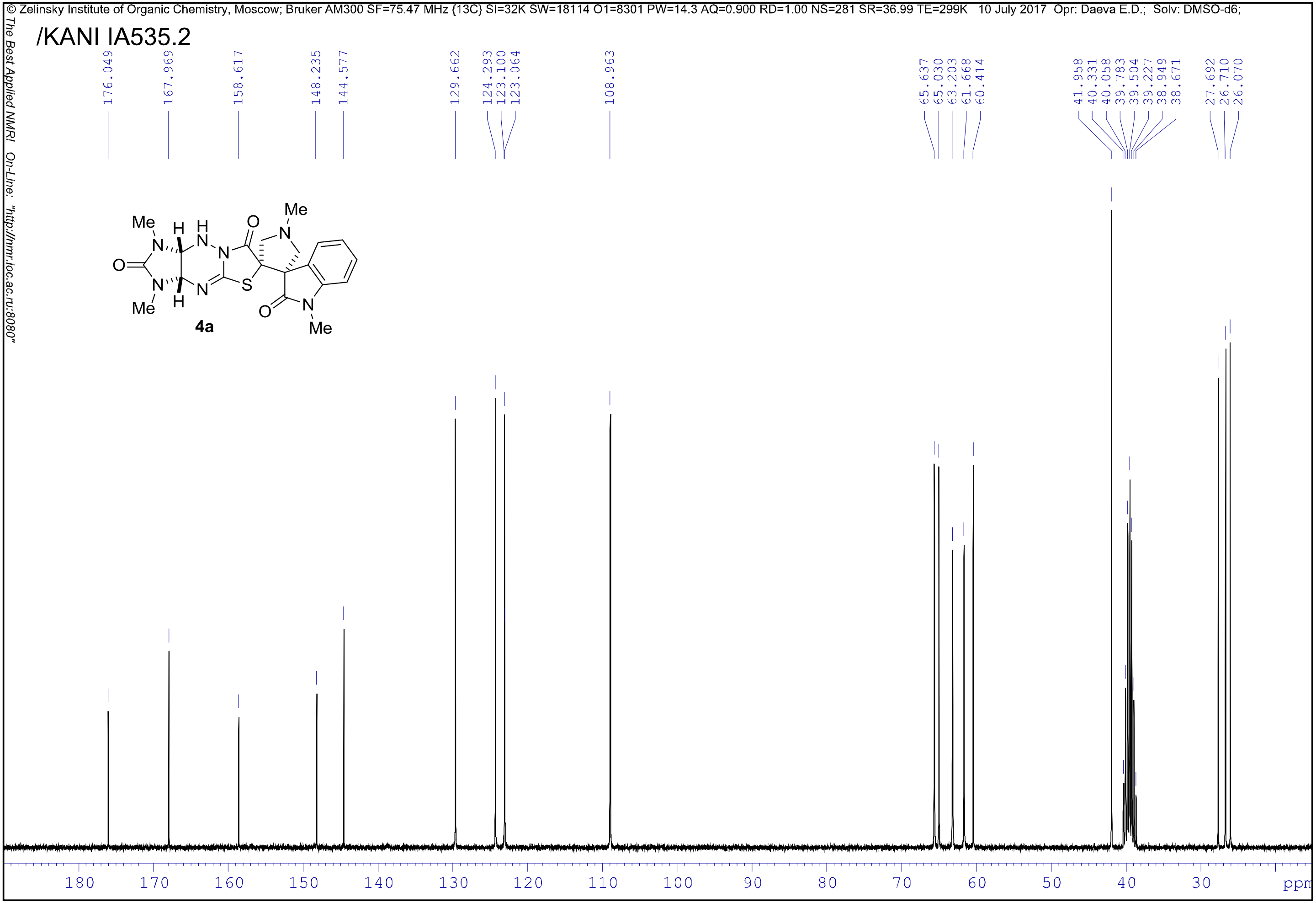
© Zelinsky Institute of Organic Chemistry, Moscow; Bruker AM300 SF=75.47 MHz {13C} SI=32K SW=18114 O1=8301 PW=14.3 AQ=0.900 RD=1.00 NS=281 SR=36.99 TE=299K 10 July 2017 Opr: Daeva E.D.; Solv: DMSO-d6;
The Best Applied NMR! On-Line: "http://nmr.ioc.ac.ru:8080"
/KANI IA535.2
176.049
167.969
158.617
148.235
144.577
129.662
124.293
123.100
123.064
108.963
65.637
65.030
63.203
61.668
60.414
41.958
40.331
40.058
39.783
39.504
39.227
38.949
38.671
27.692
26.710
26.070
4a
180 170 160 150 140 130 120 110 100 90 80 70 60 50 40 30 ppm

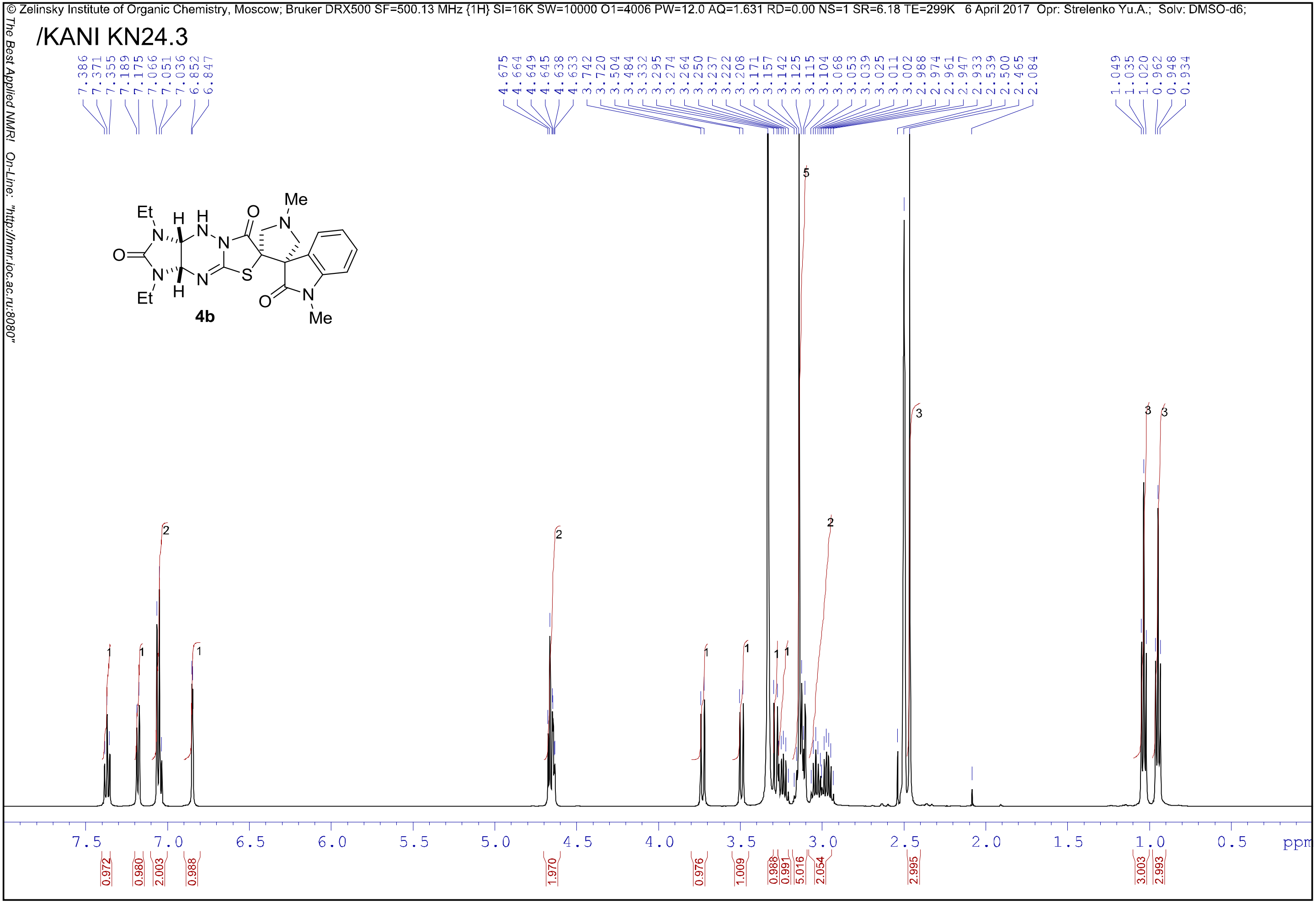
© Zelinsky Institute of Organic Chemistry, Moscow; Bruker DRX500 SF=500.13 MHz {1H} SI=16K SW=10000 O1=4006 PW=12.0 AQ=1.631 RD=0.00 NS=1 SR=6.18 TE=299K 6 April 2017 Opr: Strelenko Yu.A.; Solv: DMSO-d6;
The Best Applied NMR! On-Line: "http://nmr.ioc.ac.ru:8080"
/KANI KN24.3
7.386 7.371 7.355 7.189 7.175 7.066 7.051 7.036 6.852 6.847
4.675 4.664 4.649 4.645 4.638 4.633 3.742 3.720 3.504 3.484 3.332 3.295 3.274 3.264 3.250 3.237 3.222 3.208 3.171 3.157 3.142 3.125 3.115 3.104 3.068 3.053 3.039 3.025 3.011 3.002 2.988 2.974 2.961 2.947 2.933 2.539 2.500 2.465 2.084
1.049 1.035 1.020 0.962 0.948 0.934
Et
H
H
N
N
N
O
Me
N
O
N
N
S
Et
H
N
O
N
Me
4b
7.5 7.0 6.5 6.0 5.5 5.0 4.5 4.0 3.5 3.0 2.5 2.0 1.5 1.0 0.5 ppm
0.972 0.980 2.003 0.988 1.970 0.976 1.009 0.988 0.991 5.016 2.054 2.995 3.003 2.993

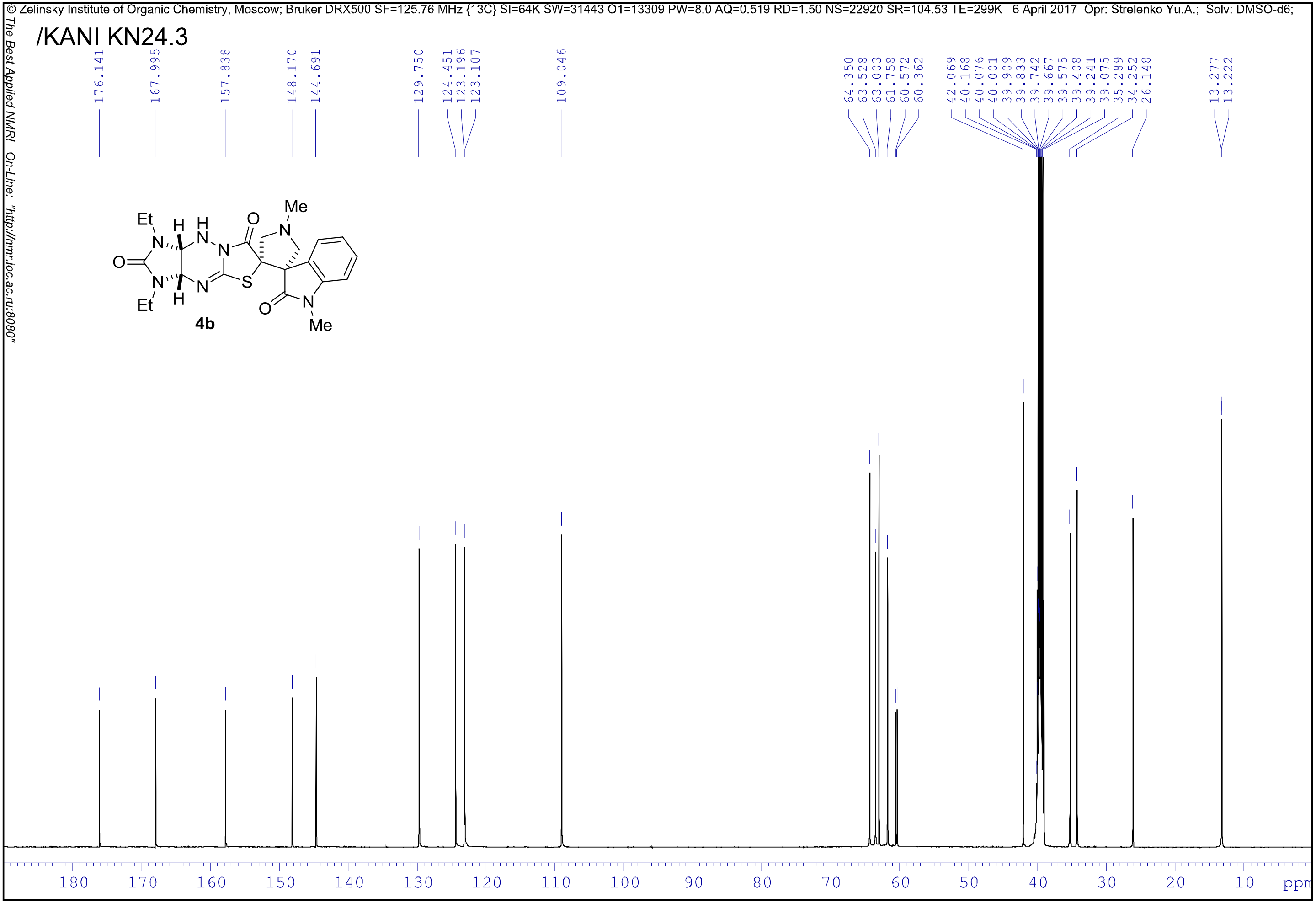
© Zelinsky Institute of Organic Chemistry, Moscow; Bruker DRX500 SF=125.76 MHz {13C} SI=64K SW=31443 O1=13309 PW=8.0 AQ=0.519 RD=1.50 NS=22920 SR=104.53 TE=299K 6 April 2017 Opr: Strelenko Yu.A.; Solv: DMSO-d6;
The Best Applied NMR! On-Line: "http://nmr.ioc.ac.ru:8080"
/KANI KN24.3
176.141
167.995
157.838
148.170
144.691
129.750
124.451
123.196
123.107
109.046
64.350
63.528
63.003
61.758
60.572
60.362
42.069
40.168
40.076
40.001
39.909
39.833
39.742
39.667
39.575
39.408
39.241
39.075
35.289
34.252
26.148
13.277
13.222
4b
180 170 160 150 140 130 120 110 100 90 80 70 60 50 40 30 20 10 ppm

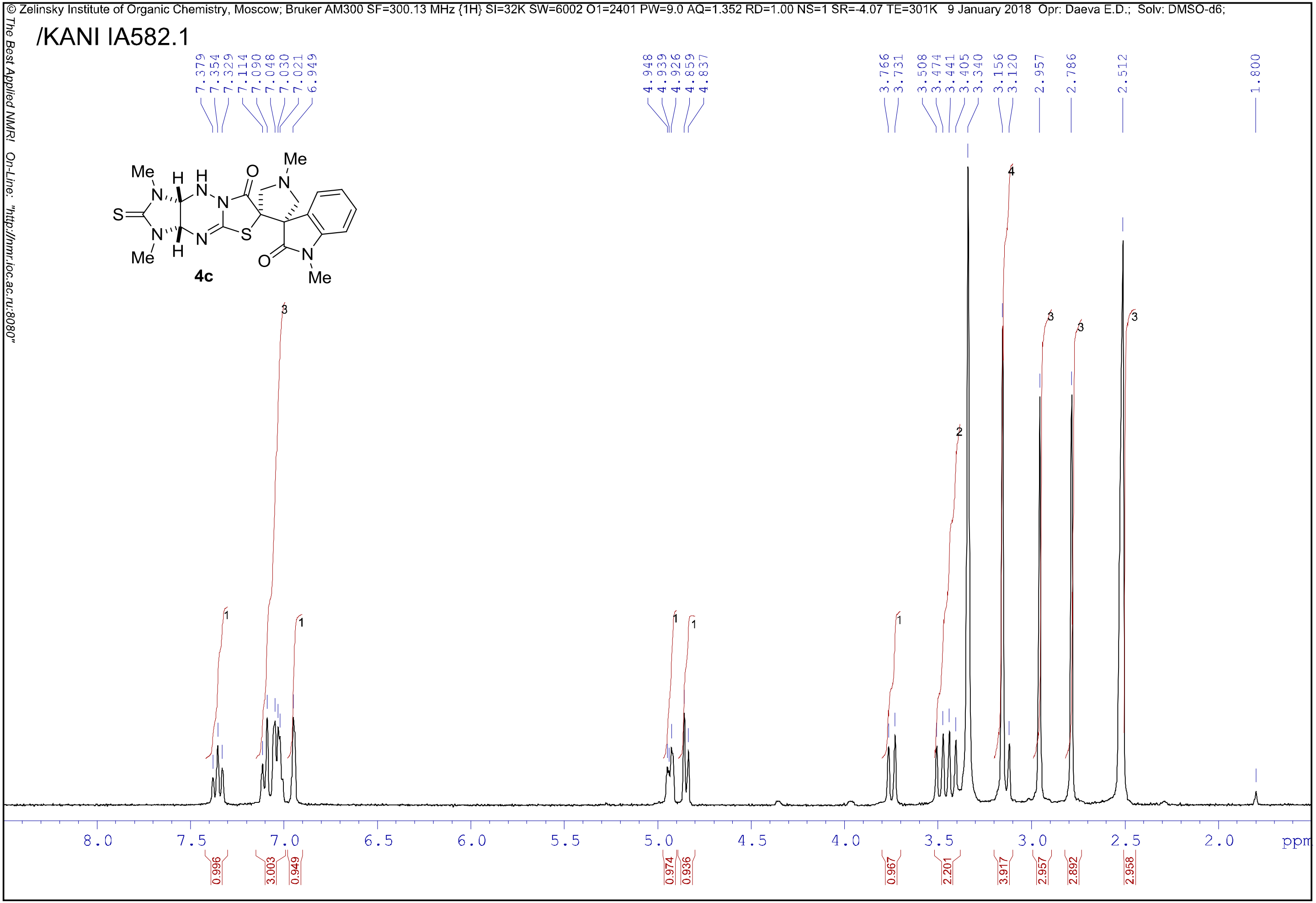
© Zelinsky Institute of Organic Chemistry, Moscow; Bruker AM300 SF=300.13 MHz {1H} SI=32K SW=6002 O1=2401 PW=9.0 AQ=1.352 RD=1.00 NS=1 SR=-4.07 TE=301K 9 January 2018 Opr: Daeva E.D.; Solv: DMSO-d6;
The Best Applied NMR! On-Line: "http://nmr.ioc.ac.ru:8080"
/KANI IA582.1
7.379 7.354 7.329 7.114 7.090 7.048 7.030 7.021 6.949
4.948 4.939 4.926 4.859 4.837
3.766 3.731 3.508 3.474 3.441 3.405 3.340 3.156 3.120 2.957 2.786 2.512 1.800
Me
H
H
N
N
O
Me
N
S
N
N
S
N
O
N
Me
H
Me
4c
8.0 7.5 7.0 6.5 6.0 5.5 5.0 4.5 4.0 3.5 3.0 2.5 2.0 ppm
0.996 3.003 0.949 0.974 0.936 0.967 2.201 3.917 2.957 2.892 2.958

© Zelinsky Institute of Organic Chemistry, Moscow; Bruker AM300 SF=75.47 MHz {13C} SI=32K SW=18114 O1=8301 PW=14.3 AQ=0.900 RD=1.00 NS=693 SR=39.28 TE=302K 10 January 2018 Opr: Daeva E.D.; Solv: DMSO-d6;

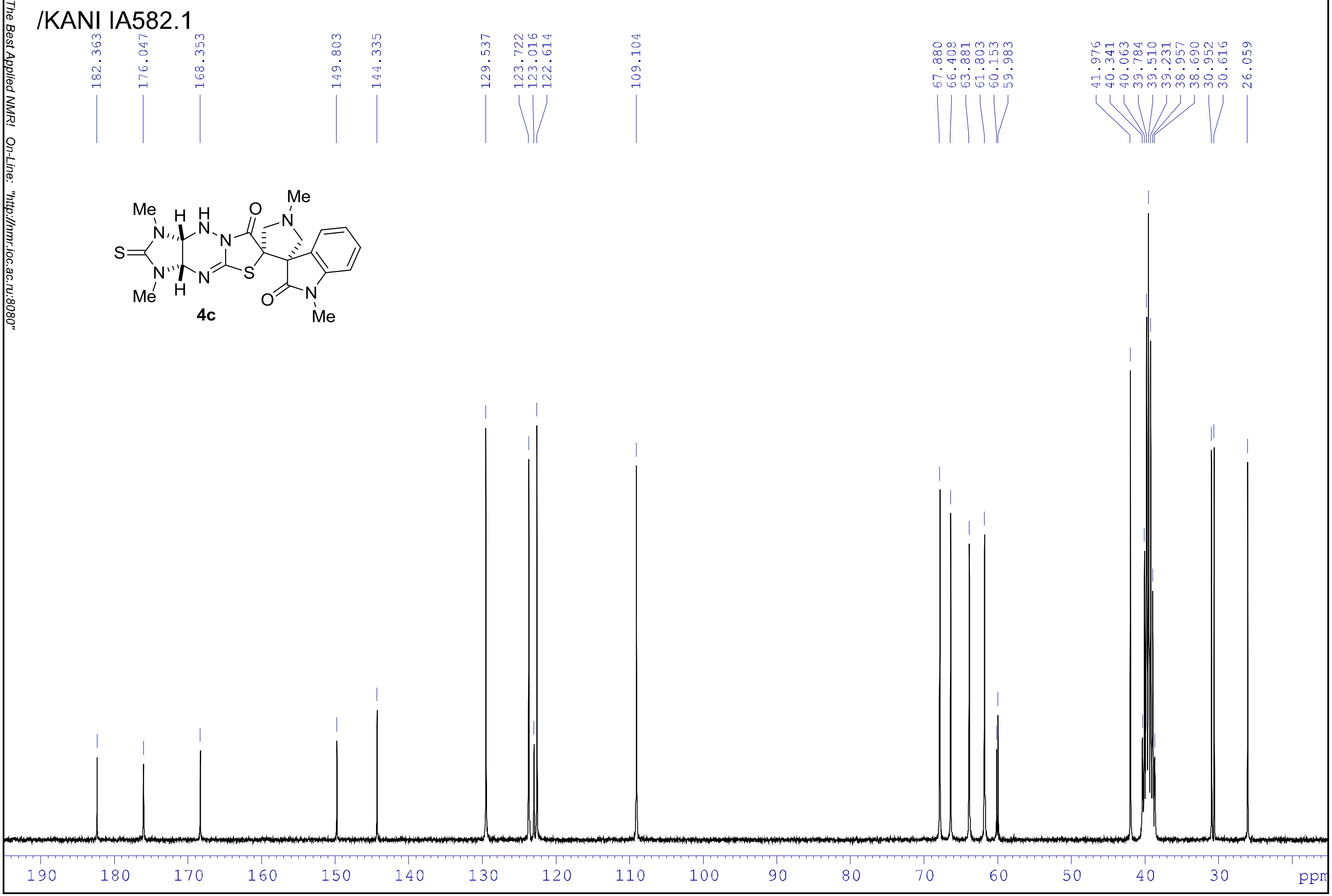

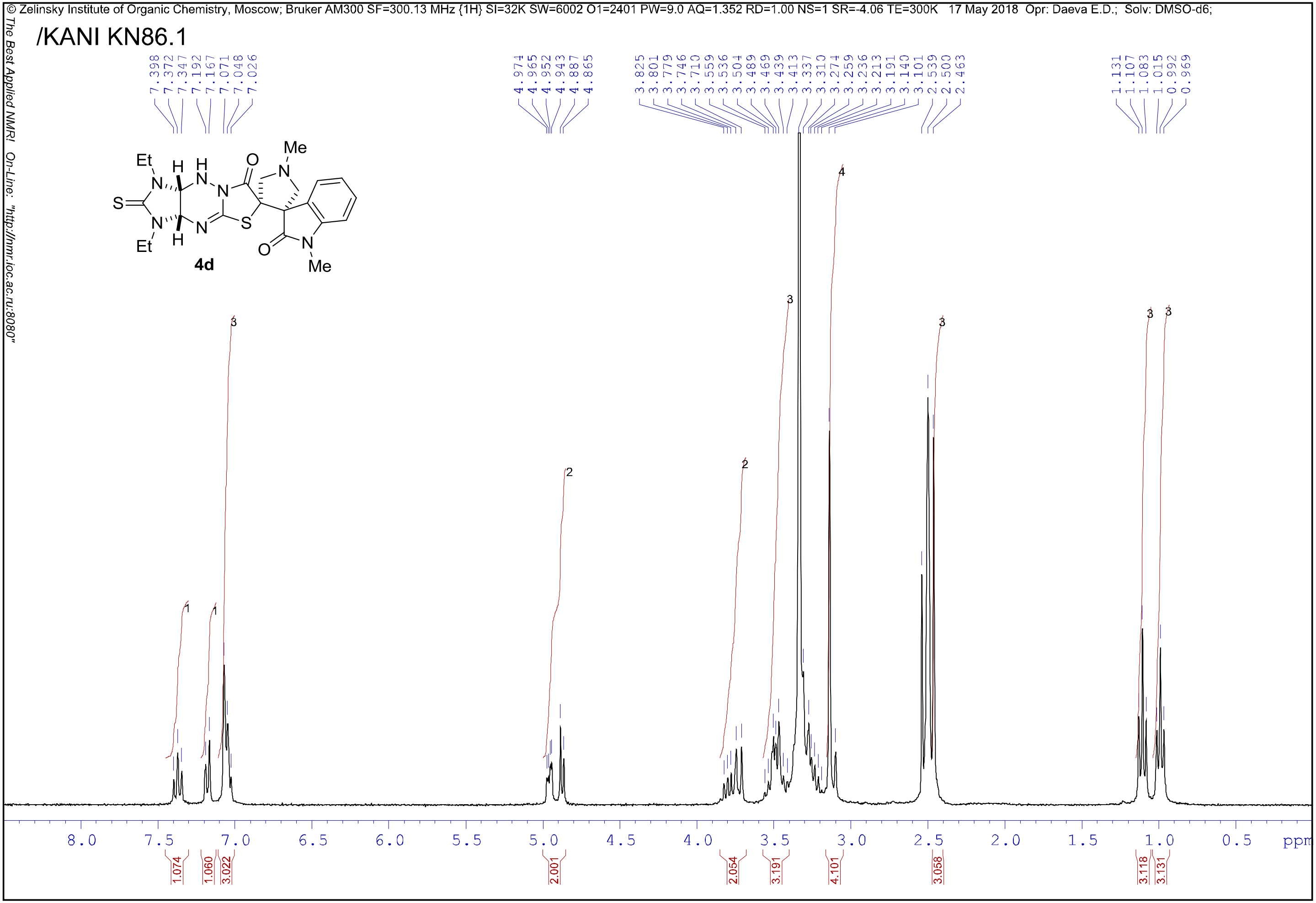
© Zelinsky Institute of Organic Chemistry, Moscow; Bruker AM300 SF=300.13 MHz {1H} SI=32K SW=6002 O1=2401 PW=9.0 AQ=1.352 RD=1.00 NS=1 SR=-4.06 TE=300K 17 May 2018 Opr: Daeva E.D.; Solv: DMSO-d6;
The Best Applied NMR! On-Line: "http://nmr.ioc.ac.ru:8080"
/KANI KN86.1
7.398 7.372 7.347 7.192 7.167 7.071 7.048 7.026
4.974 4.965 4.952 4.943 4.887 4.865
3.825 3.801 3.779 3.746 3.710 3.559 3.536 3.504 3.489 3.469 3.439 3.413 3.337 3.310 3.274 3.259 3.236 3.213 3.191 3.140 3.101 2.539 2.500 2.463
1.131 1.107 1.083 1.015 0.992 0.969
4d
8.0 7.5 7.0 6.5 6.0 5.5 5.0 4.5 4.0 3.5 3.0 2.5 2.0 1.5 1.0 0.5 ppm
1.074 1.060 3.022 2.001 2.054 3.191 4.101 3.058 3.118 3.131

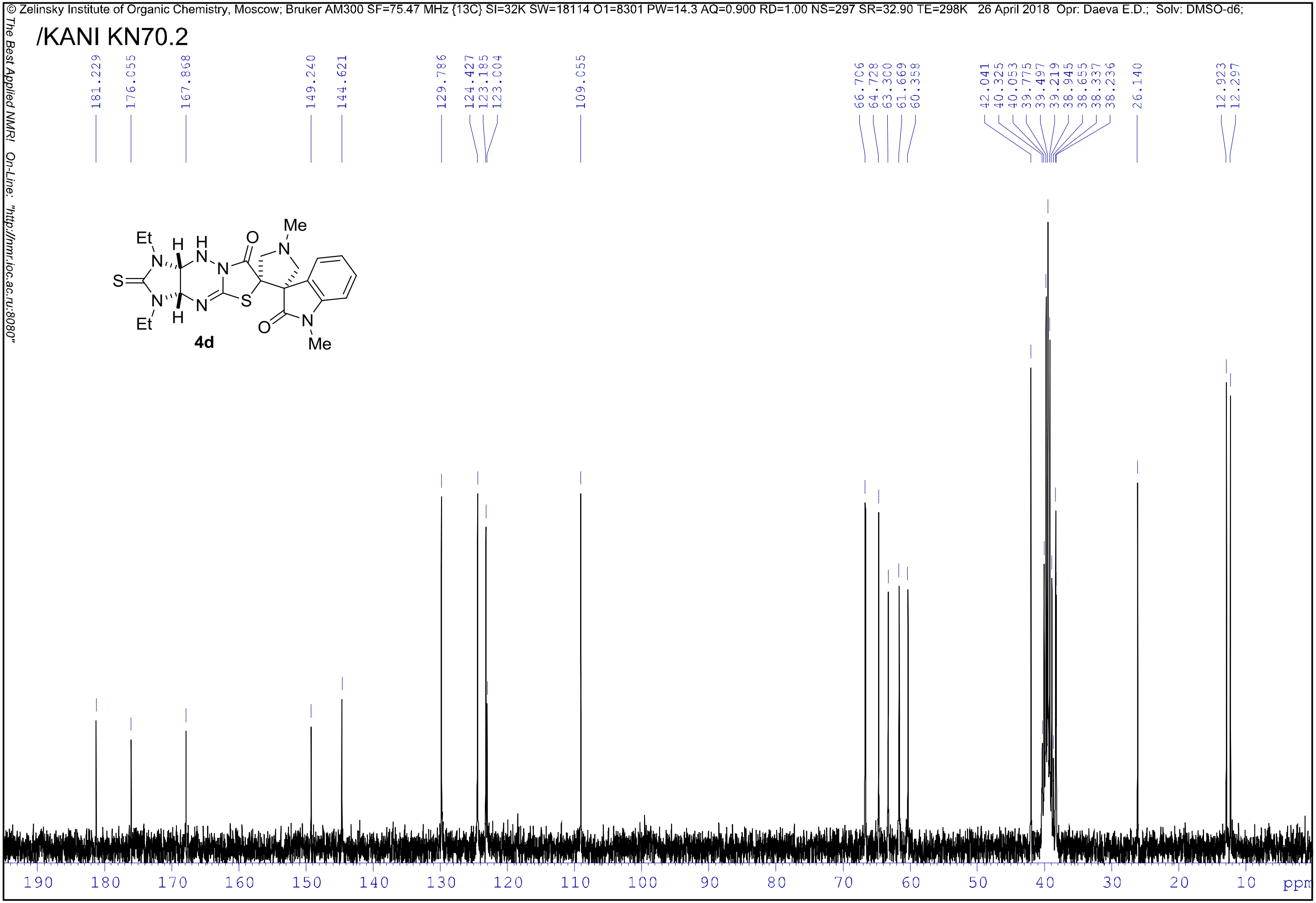
© Zelinsky Institute of Organic Chemistry, Moscow; Bruker AM300 SF=75.47 MHz {13C} SI=32K SW=18114 O1=8301 PW=14.3 AQ=0.900 RD=1.00 NS=297 SR=32.90 TE=298K 26 April 2018 Opr: Daeva E.D.; Solv: DMSO-d6;
The Best Applied NMR! On-Line: "http://nmr.ioc.ac.ru:8080"
/KANI KN70.2
181.229
176.055
167.868
149.240
144.621
129.786
124.427
123.185
123.004
109.055
66.706
64.728
63.300
61.669
60.358
42.041
40.325
40.053
39.775
39.497
39.219
38.945
38.655
38.337
38.236
26.140
12.923
12.297
4d
190 180 170 160 150 140 130 120 110 100 90 80 70 60 50 40 30 20 10 ppm

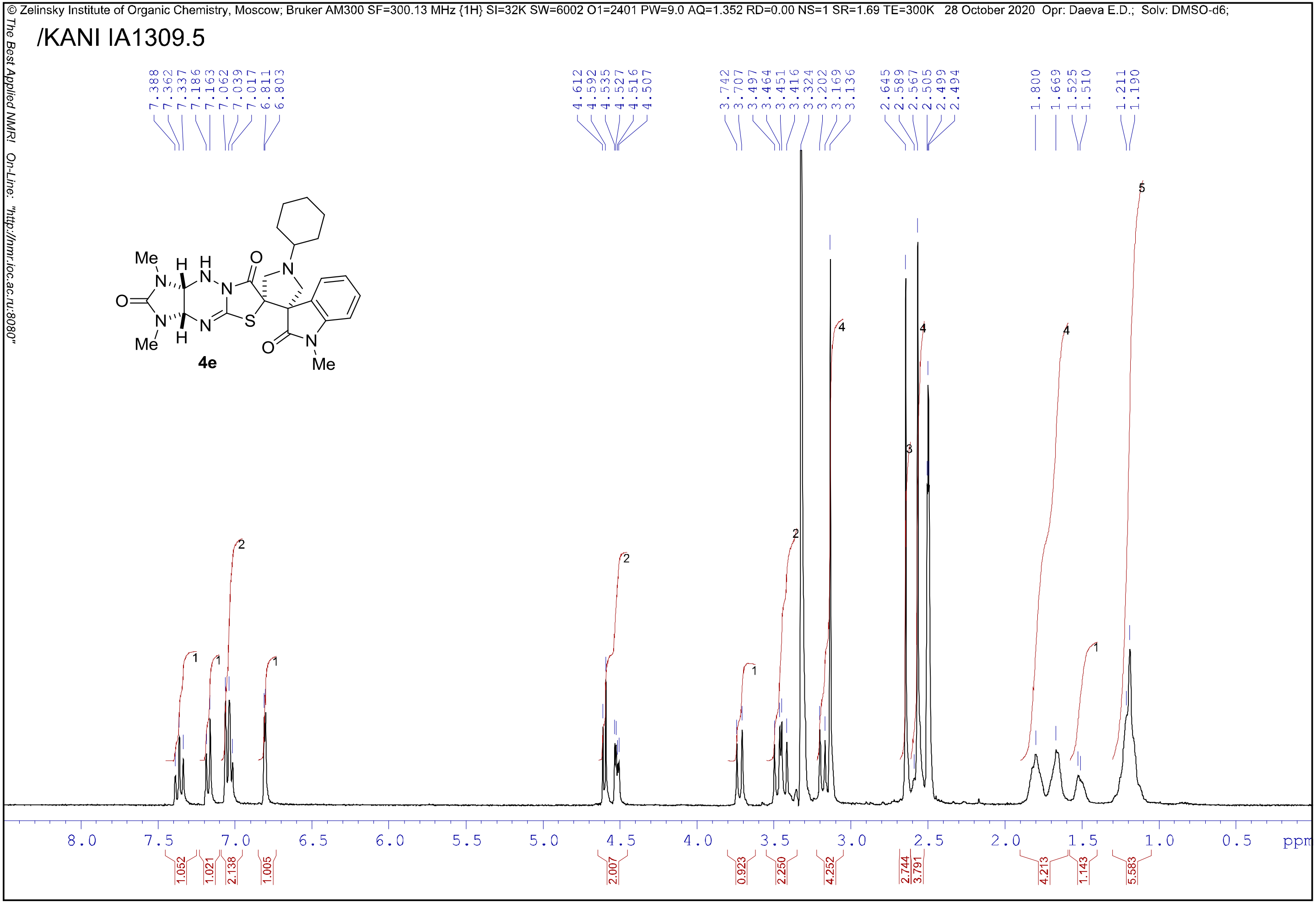
© Zelinsky Institute of Organic Chemistry, Moscow; Bruker AM300 SF=300.13 MHz {1H} SI=32K SW=6002 O1=2401 PW=9.0 AQ=1.352 RD=0.00 NS=1 SR=1.69 TE=300K 28 October 2020 Opr: Daeva E.D.; Solv: DMSO-d6;
The Best Applied NMR! On-Line: "http://nmr.ioc.ac.ru:8080"
/KANI IA1309.5
7.388 7.362 7.337 7.186 7.163 7.062 7.039 7.017 6.811 6.803
4.612 4.592 4.535 4.527 4.516 4.507
3.742 3.707 3.497 3.464 3.451 3.416 3.324 3.202 3.169 3.136
2.645 2.589 2.567 2.505 2.499 2.494
1.800 1.669 1.525 1.510
1.211 1.190
4e
8.0 7.5 7.0 6.5 6.0 5.5 5.0 4.5 4.0 3.5 3.0 2.5 2.0 1.5 1.0 0.5 ppm
1.052 1.021 2.138 1.005 2.007 0.923 2.250 4.252 2.744 3.791 4.213 1.143 5.583

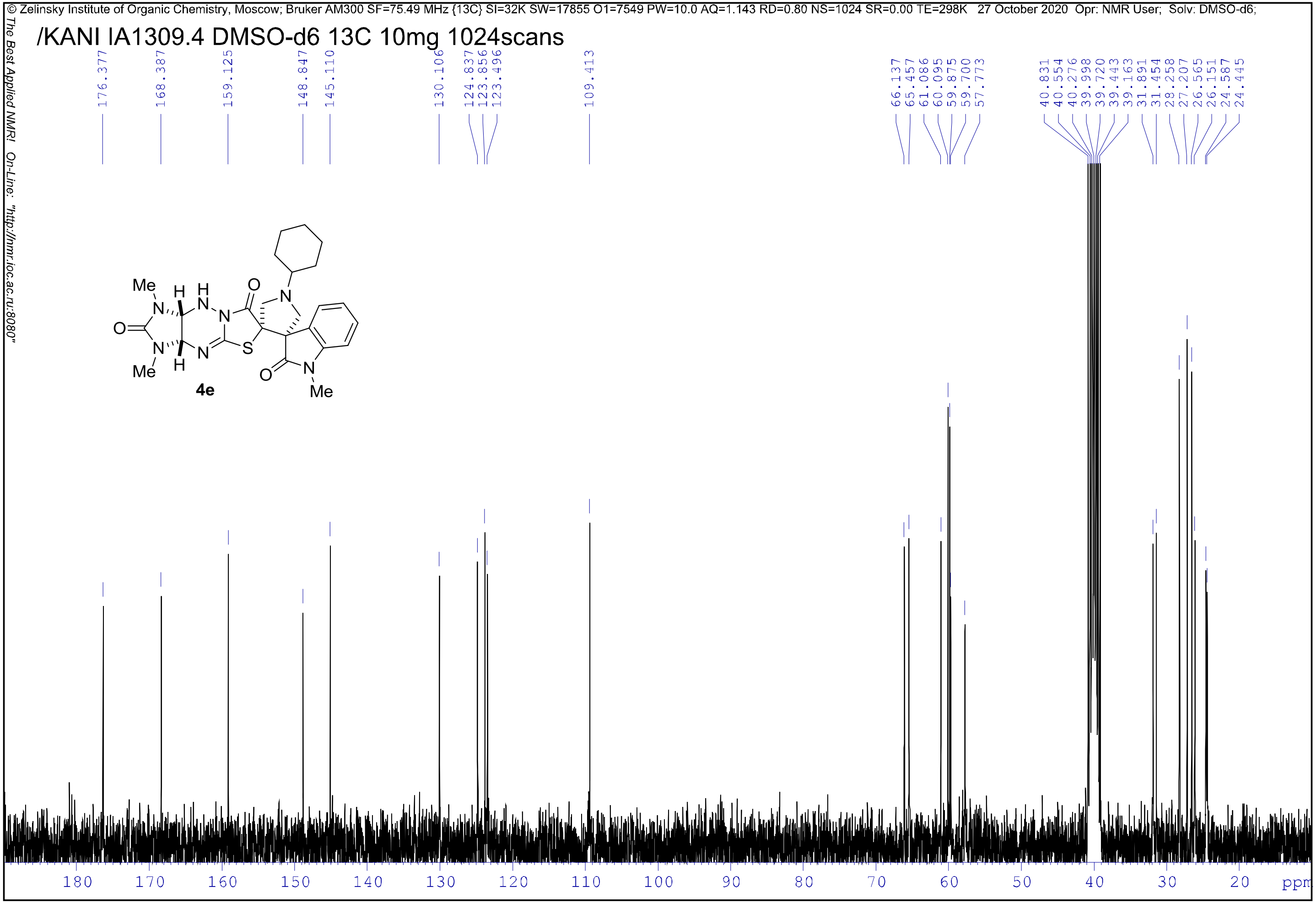
© Zelinsky Institute of Organic Chemistry, Moscow; Bruker AM300 SF=75.49 MHz {13C} SI=32K SW=17855 O1=7549 PW=10.0 AQ=1.143 RD=0.80 NS=1024 SR=0.00 TE=298K 27 October 2020 Opr: NMR User; Solv: DMSO-d6;
The Best Applied NMR! On-Line: "http://nmr.ioc.ac.ru:8080"
/KANI IA1309.4 DMSO-d6 13C 10mg 1024scans
176.377
168.387
159.125
148.847
145.110
130.106
124.837
123.856
123.496
109.413
66.137
65.457
61.086
60.095
59.875
59.700
57.773
40.831
40.554
40.276
39.998
39.720
39.443
39.163
31.891
31.454
28.258
27.207
26.565
26.151
24.587
24.445
Me
H
H
N
N
N
O
N
O
N
N
S
O
N
Me
H
4e
Me
180
170
160
150
140
130
120
110
100
90
80
70
60
50
40
30
20
ppm

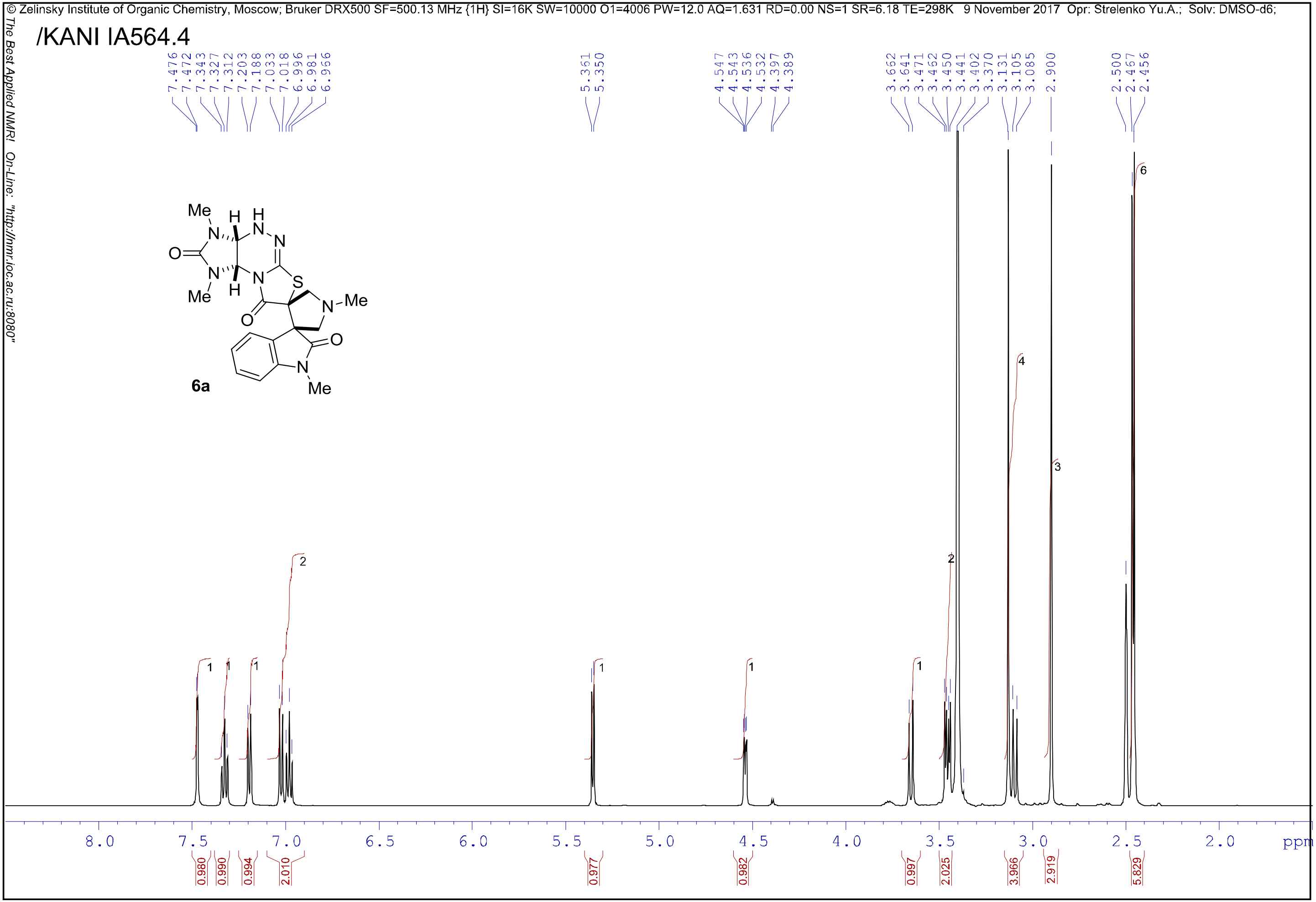
© Zelinsky Institute of Organic Chemistry, Moscow; Bruker DRX500 SF=500.13 MHz {1H} SI=16K SW=10000 O1=4006 PW=12.0 AQ=1.631 RD=0.00 NS=1 SR=6.18 TE=298K 9 November 2017 Opr: Strelenko Yu.A.; Solv: DMSO-d6;
The Best Applied NMR! On-Line: "http://nmr.ioc.ac.ru:8080"
/KANI IA564.4
7.476 7.472 7.343 7.327 7.312 7.203 7.188 7.033 7.018 6.996 6.981 6.956
5.361 5.350
4.547 4.543 4.536 4.532 4.397 4.389
3.662 3.641 3.471 3.462 3.450 3.441 3.402 3.370 3.131 3.105 3.085 2.900
2.500 2.457 2.456
Me
H
H
N
N
N
O
N
N
S
N
Me
Me
H
O
N
O
N
Me
6a
1 1 1 2 1 1 1 2 4 3 6
8.0 7.5 7.0 6.5 6.0 5.5 5.0 4.5 4.0 3.5 3.0 2.5 2.0 ppm
0.980 0.990 0.994 2.010 0.977 0.982 0.997 2.025 3.966 2.919 5.829

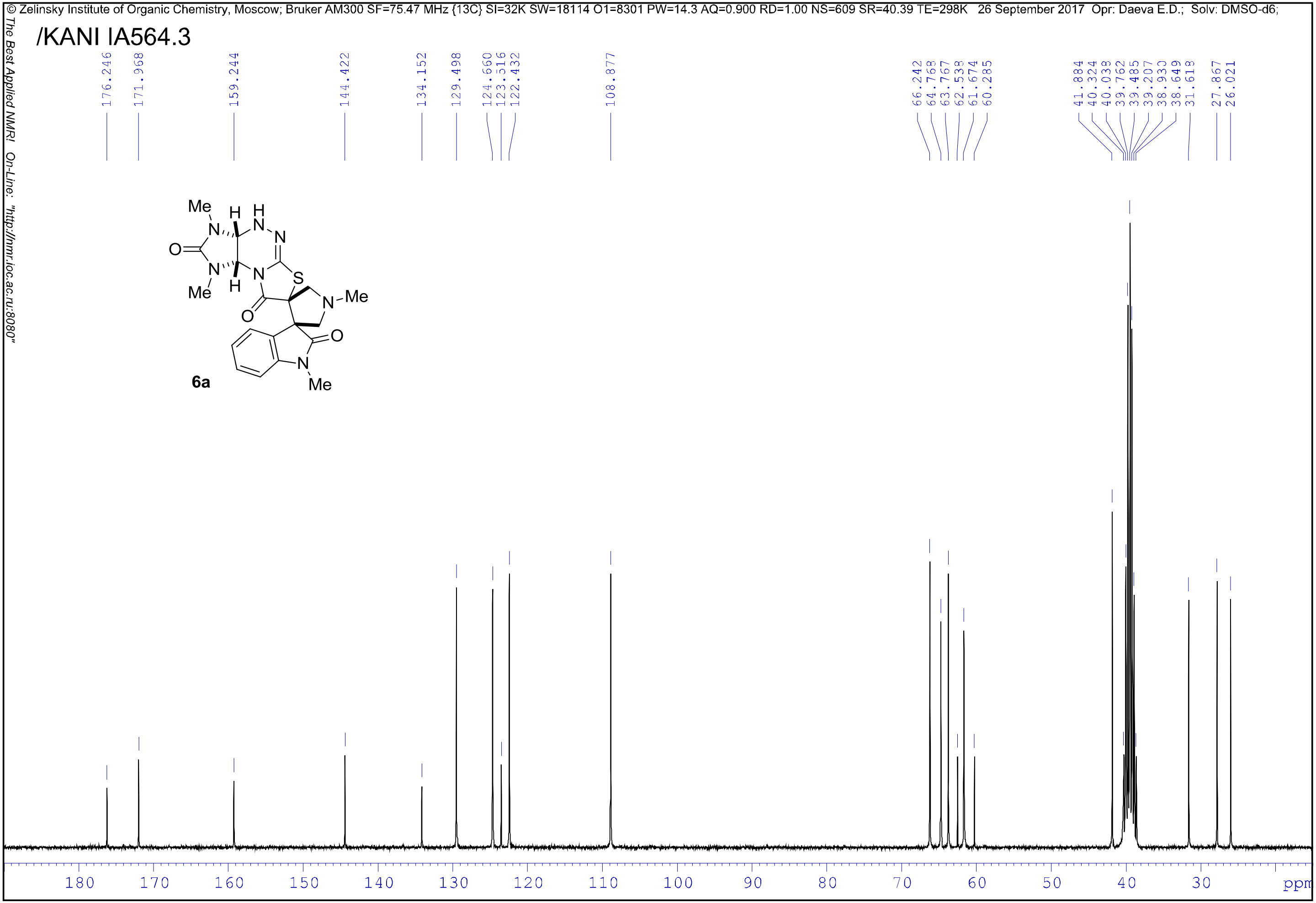
© Zelinsky Institute of Organic Chemistry, Moscow; Bruker AM300 SF=75.47 MHz {13C} SI=32K SW=18114 O1=8301 PW=14.3 AQ=0.900 RD=1.00 NS=609 SR=40.39 TE=298K 26 September 2017 Opr: Daeva E.D.; Solv: DMSO-d6;
The Best Applied NMR! On-Line: "http://nmr.ioc.ac.ru:8080"
/KANI IA564.3
176.246
171.968
159.244
144.422
134.152
129.498
124.660
123.516
122.432
108.877
66.242
64.768
63.767
62.538
61.674
60.285
41.884
40.324
40.038
39.762
39.485
39.207
38.930
38.649
31.618
27.867
26.021
6a
180
170
160
150
140
130
120
110
100
90
80
70
60
50
40
30
ppm

© Zelinsky Institute of Organic Chemistry, Moscow; Bruker DRX500 SF=500.13 MHz {1H-1H}NOESY SI=1024x512 SW=3894 O1=2106 PW=14.4 AQ=0.113 RD=1.00 NS=4x256 SR=6.18 TE=296K 26 September 2017 Opr: Strelenko Yu.A.; Solv: DMSO-d6;

The Best Applied NMR! On-Line: "http://nmr.ioc.ac.ru:8080"

/KANI IA564.2
NOESY

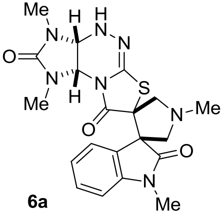

6a

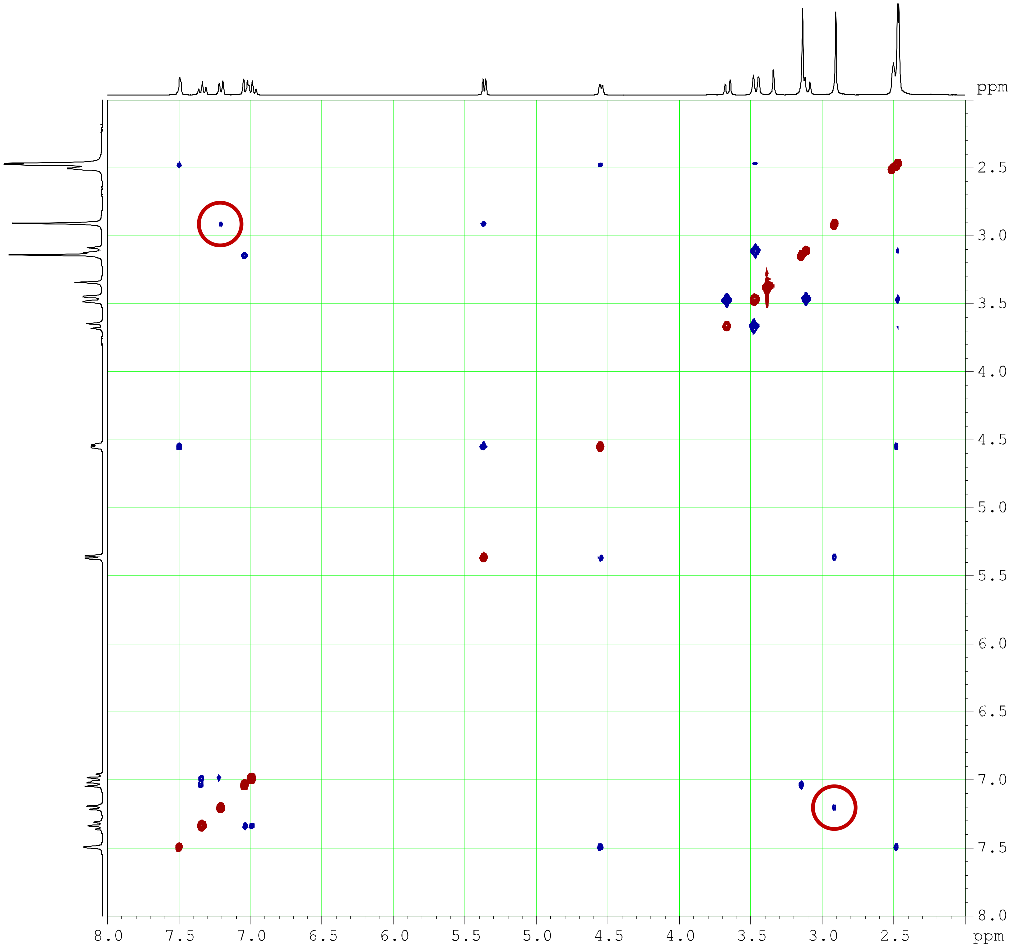

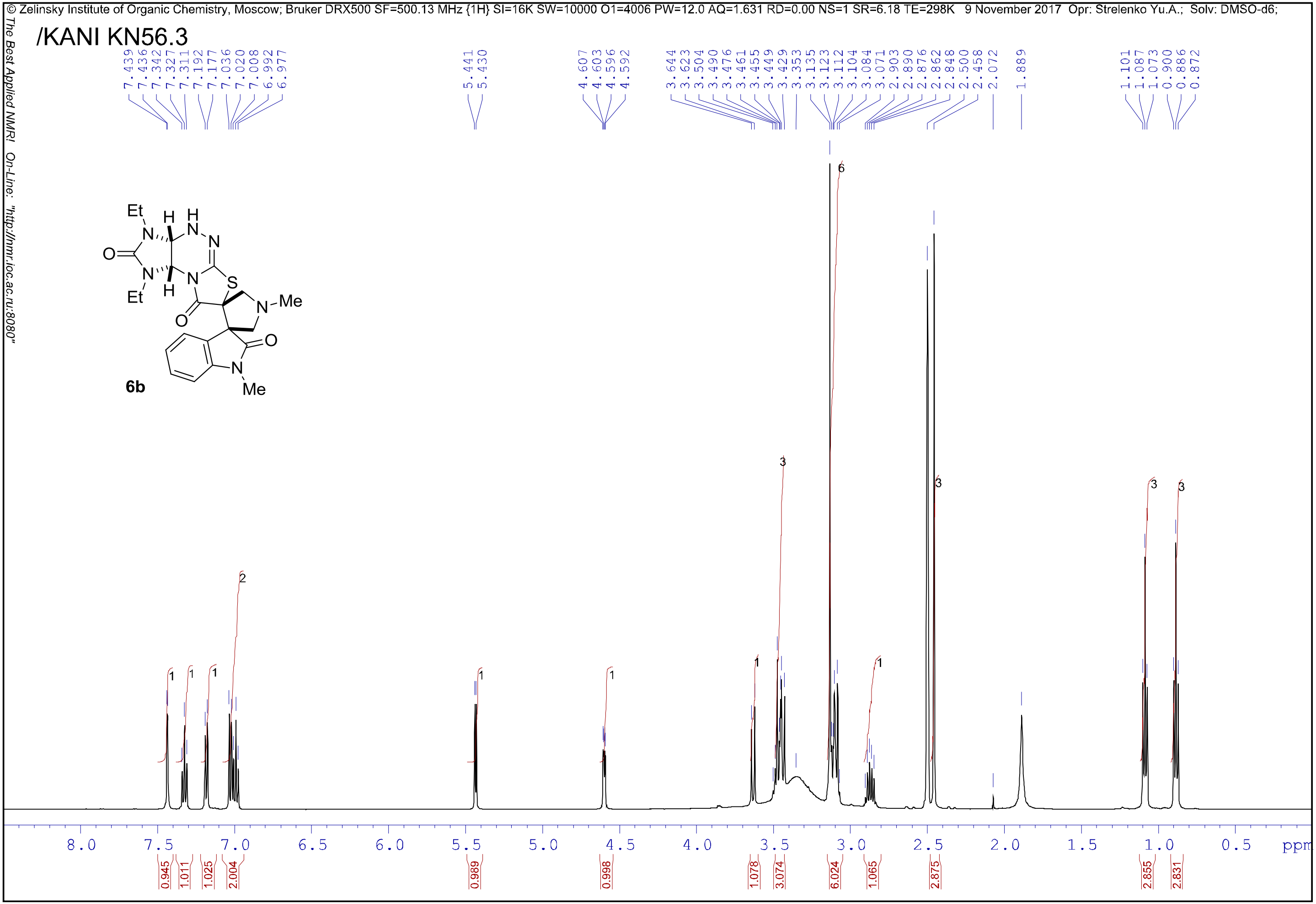
© Zelinsky Institute of Organic Chemistry, Moscow; Bruker DRX500 SF=500.13 MHz {1H} SI=16K SW=10000 O1=4006 PW=12.0 AQ=1.631 RD=0.00 NS=1 SR=6.18 TE=298K 9 November 2017 Opr: Strelenko Yu.A.; Solv: DMSO-d6;
/KANI KN56.3
The Best Applied NMR! On-Line: "http://nmr.ioc.ac.ru:8080"
7.439 7.436 7.342 7.327 7.311 7.192 7.177 7.036 7.020 7.008 6.992 6.977
5.441 5.430
4.607 4.603 4.596 4.592
3.644 3.623 3.504 3.490 3.476 3.461 3.455 3.449 3.429 3.353 3.135 3.123 3.112 3.104 3.084 3.071 2.903 2.890 2.876 2.862 2.848 2.500 2.458 2.072
1.889
1.101 1.087 1.073 0.900 0.886 0.872
6b
0.945 1.011 1.025 2.004 0.989 0.998 1.078 3.074 6.024 1.065 2.875 2.855 2.831
8.0 7.5 7.0 6.5 6.0 5.5 5.0 4.5 4.0 3.5 3.0 2.5 2.0 1.5 1.0 0.5 ppm

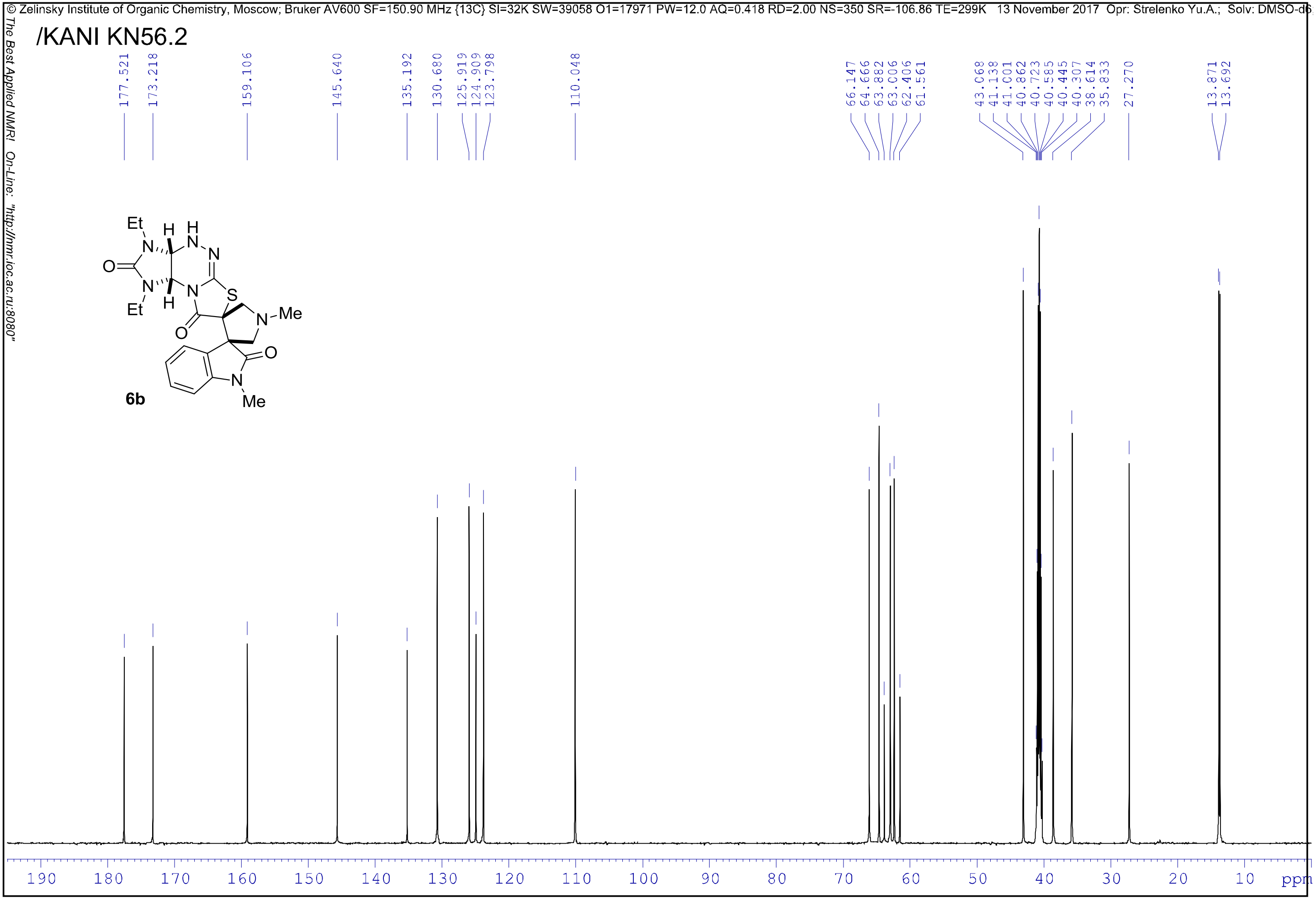
© Zelinsky Institute of Organic Chemistry, Moscow; Bruker AV600 SF=150.90 MHz {13C} SI=32K SW=39058 O1=17971 PW=12.0 AQ=0.418 RD=2.00 NS=350 SR=-106.86 TE=299K 13 November 2017 Opr: Strelenko Yu.A.; Solv: DMSO-d6
The Best Applied NMR! On-Line: "http://nmr.ioc.ac.ru:8080"
/KANI KN56.2
177.521
173.218
159.106
145.640
135.192
130.680
125.919
124.909
123.798
110.048
66.147
64.666
63.882
63.006
62.406
61.561
43.068
41.138
41.001
40.862
40.723
40.585
40.445
40.307
38.614
35.833
27.270
13.871
13.692
6b
190 180 170 160 150 140 130 120 110 100 90 80 70 60 50 40 30 20 10 ppm

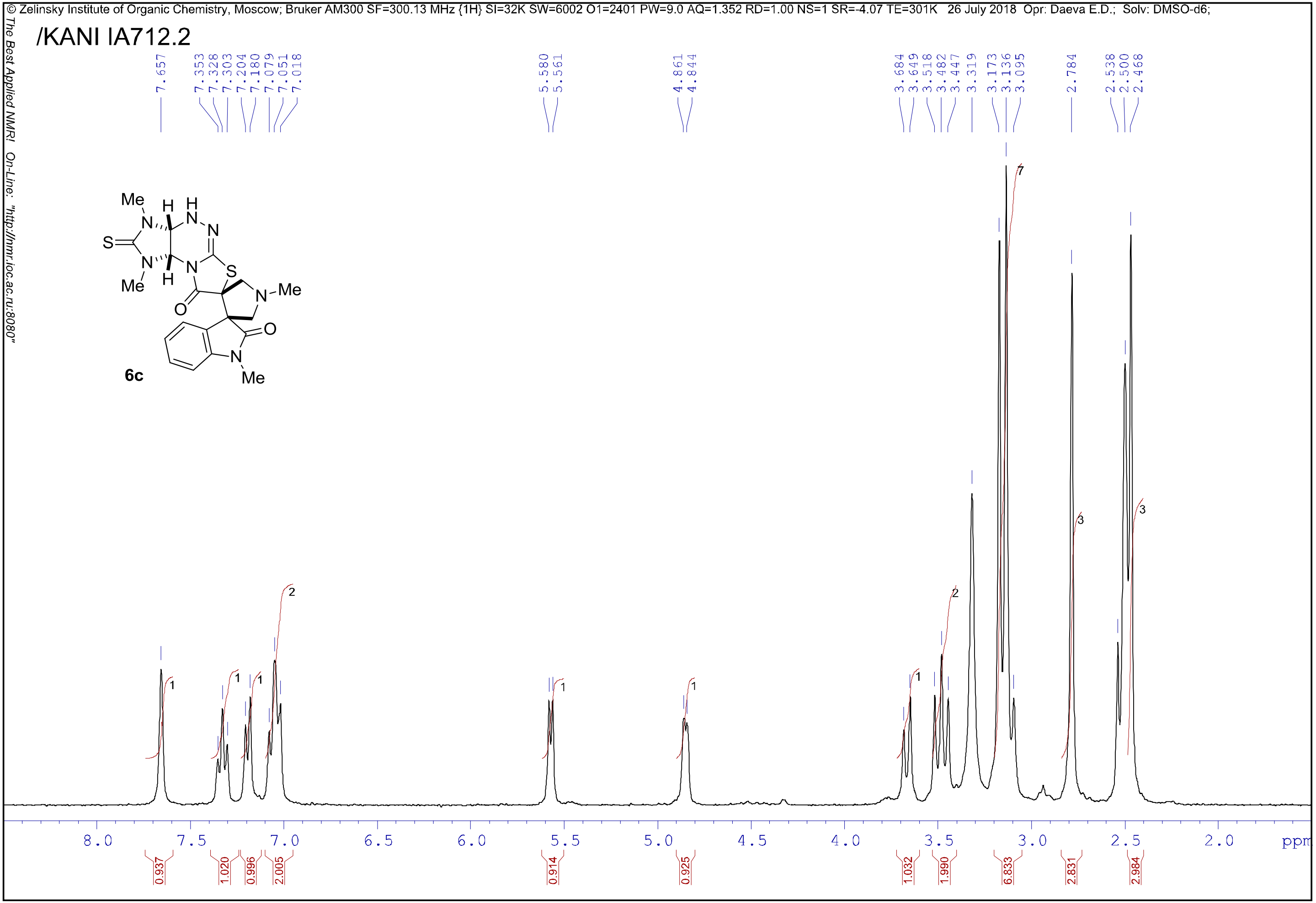
© Zelinsky Institute of Organic Chemistry, Moscow; Bruker AM300 SF=300.13 MHz {1H} SI=32K SW=6002 O1=2401 PW=9.0 AQ=1.352 RD=1.00 NS=1 SR=-4.07 TE=301K 26 July 2018 Opr: Daeva E.D.; Solv: DMSO-d6;
The Best Applied NMR! On-Line: "http://nmr.ioc.ac.ru:8080"
/KANI IA712.2
7.657
7.353
7.328
7.303
7.204
7.180
7.079
7.051
7.018
5.580
5.561
4.861
4.844
3.684
3.649
3.518
3.482
3.447
3.319
3.173
3.136
3.095
2.784
2.538
2.500
2.468
Me
H
H
N
N
N
S
N
N
S
N
Me
H
O
N
Me
O
N
Me
6c
8.0
7.5
7.0
6.5
6.0
5.5
5.0
4.5
4.0
3.5
3.0
2.5
2.0
ppm
0.937
1.020
0.996
2.005
0.914
0.925
1.032
1.990
6.833
2.831
2.984

© Zelinsky Institute of Organic Chemistry, Moscow; Bruker AM300 SF=75.47 MHz {13C} SI=32K SW=18114 O1=8301 PW=14.3 AQ=0.900 RD=1.00 NS=511 SR=39.76 TE=300K 26 July 2018 Opr: Daeva E.D.; Solv: DMSO-d6;

The Best Applied NMR! On-Line: "http://nmr.ioc.ac.ru:8080"

/KANI IA712.2

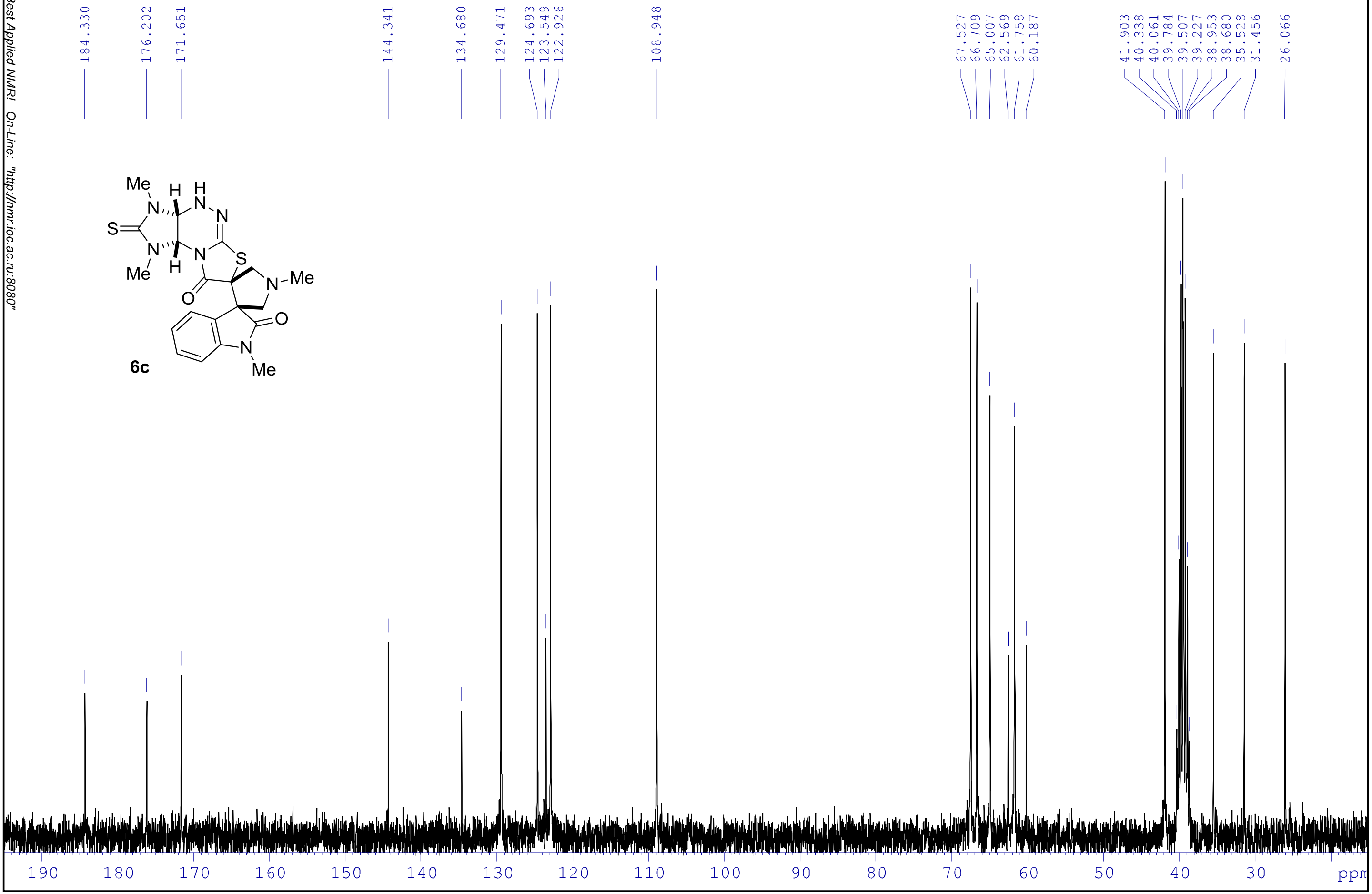

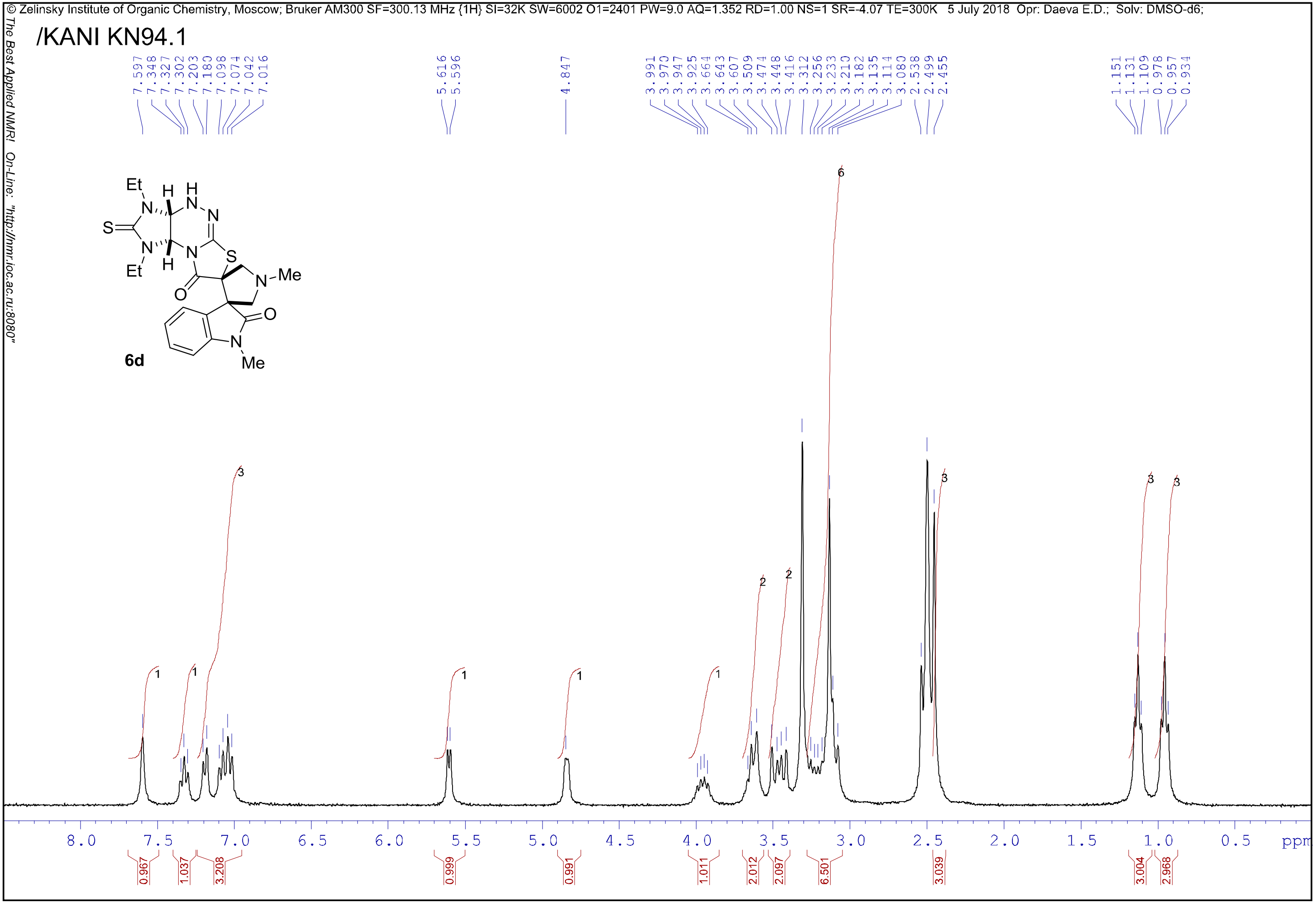
© Zelinsky Institute of Organic Chemistry, Moscow; Bruker AM300 SF=300.13 MHz {1H} SI=32K SW=6002 O1=2401 PW=9.0 AQ=1.352 RD=1.00 NS=1 SR=-4.07 TE=300K 5 July 2018 Opr: Daeva E.D.; Solv: DMSO-d6;
The Best Applied NMR! On-Line: "http://nmr.ioc.ac.ru:8080"
/KANI KN94.1
7.597 7.348 7.327 7.302 7.203 7.180 7.098 7.074 7.042 7.016
5.616 5.596
4.847
3.991 3.970 3.947 3.925 3.664 3.643 3.607 3.509 3.474 3.448 3.416 3.312 3.256 3.233 3.210 3.182 3.135 3.114 3.080 2.538 2.499 2.455
1.151 1.131 1.109 0.978 0.957 0.934
6d
8.0 7.5 7.0 6.5 6.0 5.5 5.0 4.5 4.0 3.5 3.0 2.5 2.0 1.5 1.0 0.5 ppm
0.967 1.037 3.208 0.999 0.991 1.011 2.012 2.097 6.501 3.039 3.004 2.968

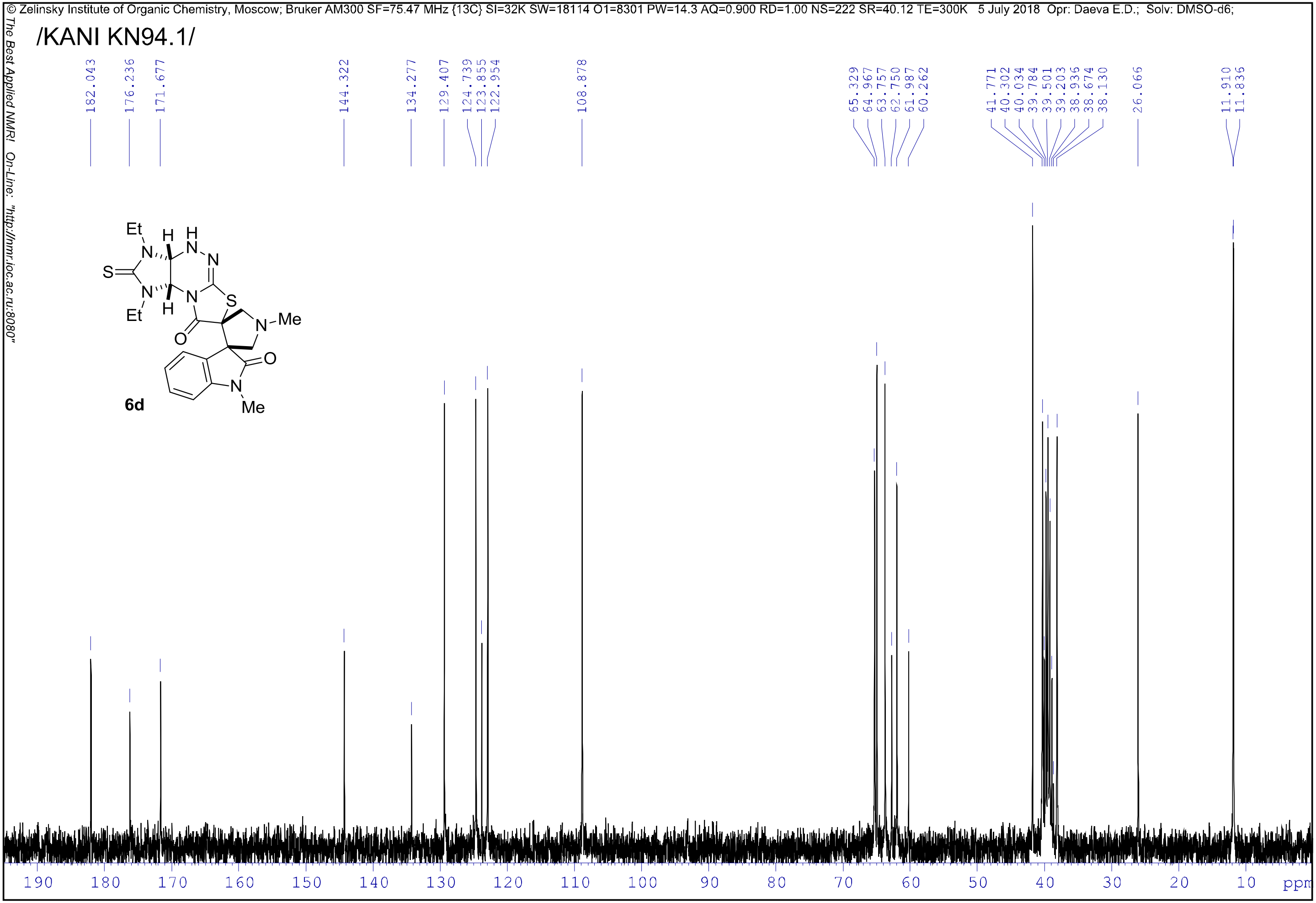
© Zelinsky Institute of Organic Chemistry, Moscow; Bruker AM300 SF=75.47 MHz {13C} SI=32K SW=18114 O1=8301 PW=14.3 AQ=0.900 RD=1.00 NS=222 SR=40.12 TE=300K 5 July 2018 Opr: Daeva E.D.; Solv: DMSO-d6;
/KANI KN94.1/
The Best Applied NMR! On-Line: "http://nmr.ioc.ac.ru:8080"
182.043
176.236
171.677
144.322
134.277
129.407
124.739
123.855
122.954
108.878
65.329
64.967
63.757
62.750
61.987
60.262
41.771
40.302
40.034
39.784
39.501
39.203
38.936
38.674
38.130
26.066
11.910
11.836
Et
H
H
N
N
N
S
N
N
S
Et
H
O
N
Me
O
N
Me
6d
190
180
170
160
150
140
130
120
110
100
90
80
70
60
50
40
30
20
10
ppm

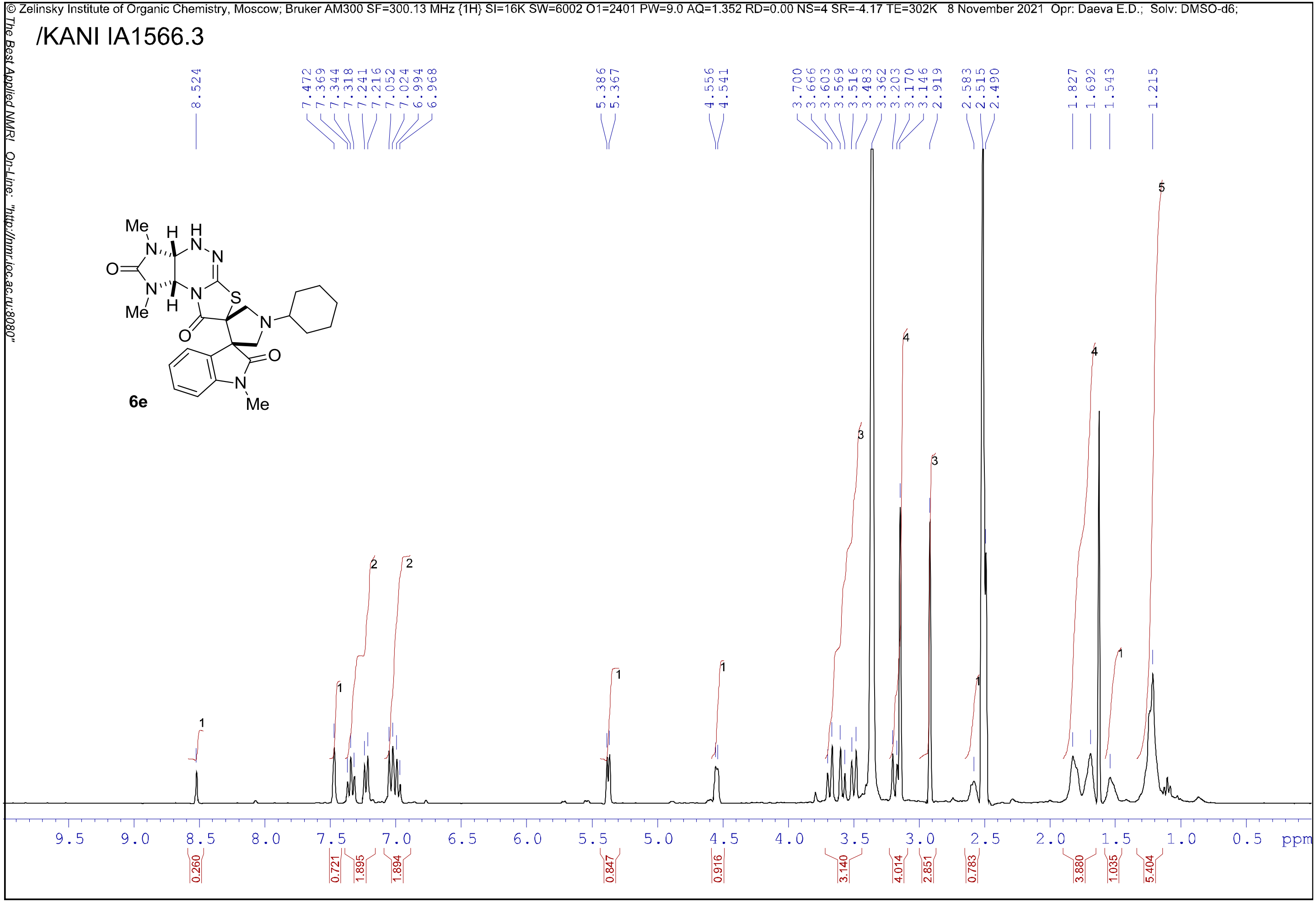
© Zelinsky Institute of Organic Chemistry, Moscow; Bruker AM300 SF=300.13 MHz {1H} SI=16K SW=6002 O1=2401 PW=9.0 AQ=1.352 RD=0.00 NS=4 SR=-4.17 TE=302K 8 November 2021 Opr: Daeva E.D.; Solv: DMSO-d6;
The Best Applied NMR! On-Line: "http://nmr.ioc.ac.ru:8080"
/KANI IA1566.3
8.524
7.472 7.369 7.344 7.318 7.241 7.216 7.052 7.024 6.994 6.968
5.386 5.367
4.556 4.541
3.700 3.666 3.603 3.569 3.516 3.483 3.362 3.203 3.170 3.146 2.919
2.583 2.515 2.490
1.827 1.692 1.543
1.215
6e
Me
H
N
O
S
ppm
0.260 0.721 1.895 1.894 0.847 0.916 3.140 4.014 2.851 0.783 3.880 1.035 5.404

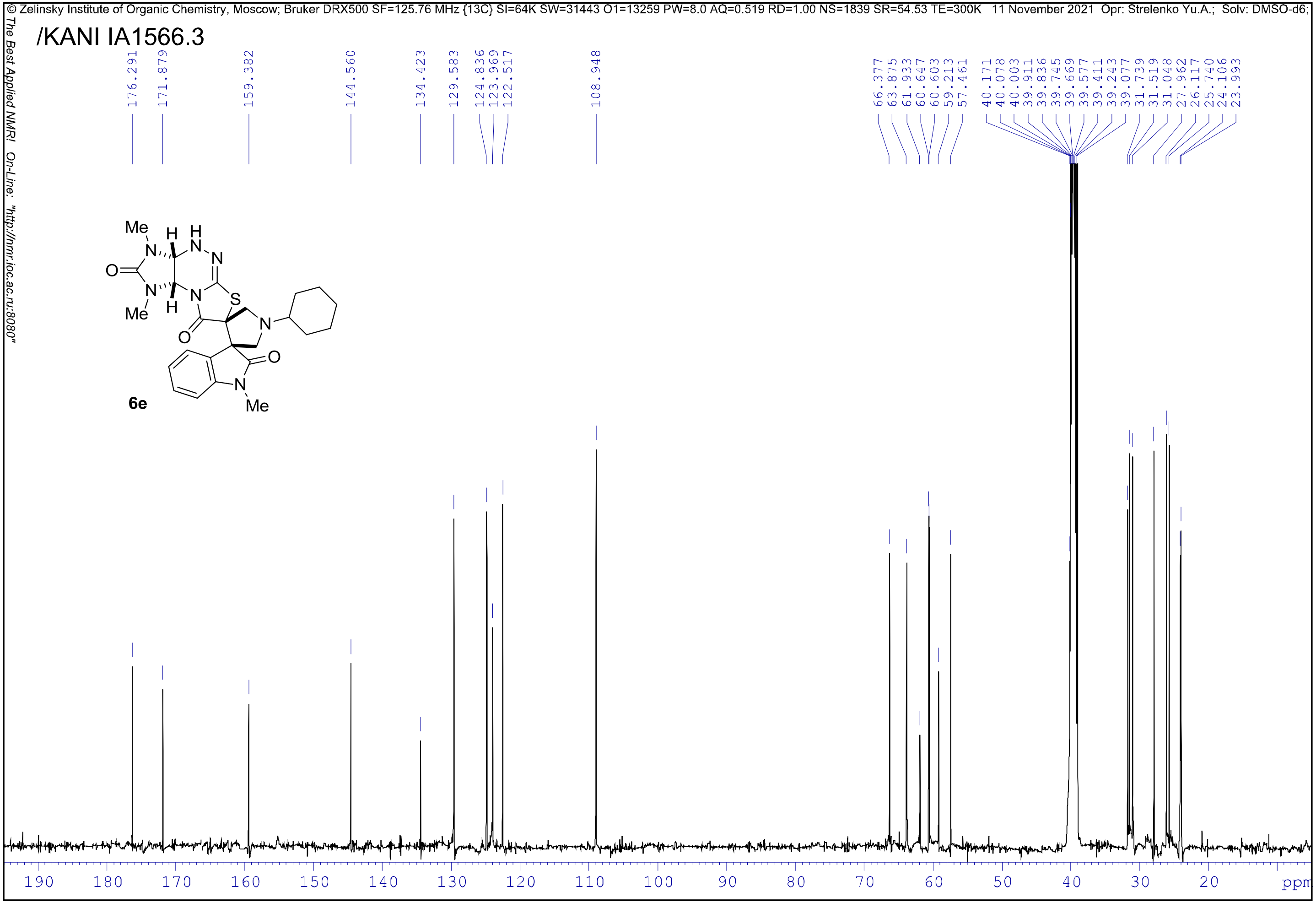
© Zelinsky Institute of Organic Chemistry, Moscow; Bruker DRX500 SF=125.76 MHz {13C} SI=64K SW=31443 O1=13259 PW=8.0 AQ=0.519 RD=1.00 NS=1839 SR=54.53 TE=300K 11 November 2021 Opr: Strelenko Yu.A.; Solv: DMSO-d6;
The Best Applied NMR! On-Line: "http://nmr.ioc.ac.ru:8080"
/KANI IA1566.3
176.291
171.879
159.382
144.560
134.423
129.583
124.836
123.969
122.517
108.948
66.377
63.875
61.933
60.647
60.603
59.213
57.461
40.171
40.078
40.003
39.911
39.836
39.745
39.669
39.577
39.411
39.243
39.077
31.739
31.519
31.048
27.962
26.117
25.740
24.106
23.993
Me
H
H
N
N
N
O
N
S
N
Me
H
N
O
N
O
6e
N
Me
190 180 170 160 150 140 130 120 110 100 90 80 70 60 50 40 30 20 ppm

© Zelinsky Institute of Organic Chemistry, Moscow; Bruker DRX500 SF=500.13 MHz {1H} SI=16K SW=10000 O1=4006 PW=12.0 AQ=1.631 RD=0.00 NS=1 SR=6.18 TE=298K 25 May 2017 Opr: Strelenko Yu.A.; Solv: DMSO-d6;

The Best Applied NMR! On-Line: "http://nmr.ioc.ac.ru:8080"

/KANI KN22.3

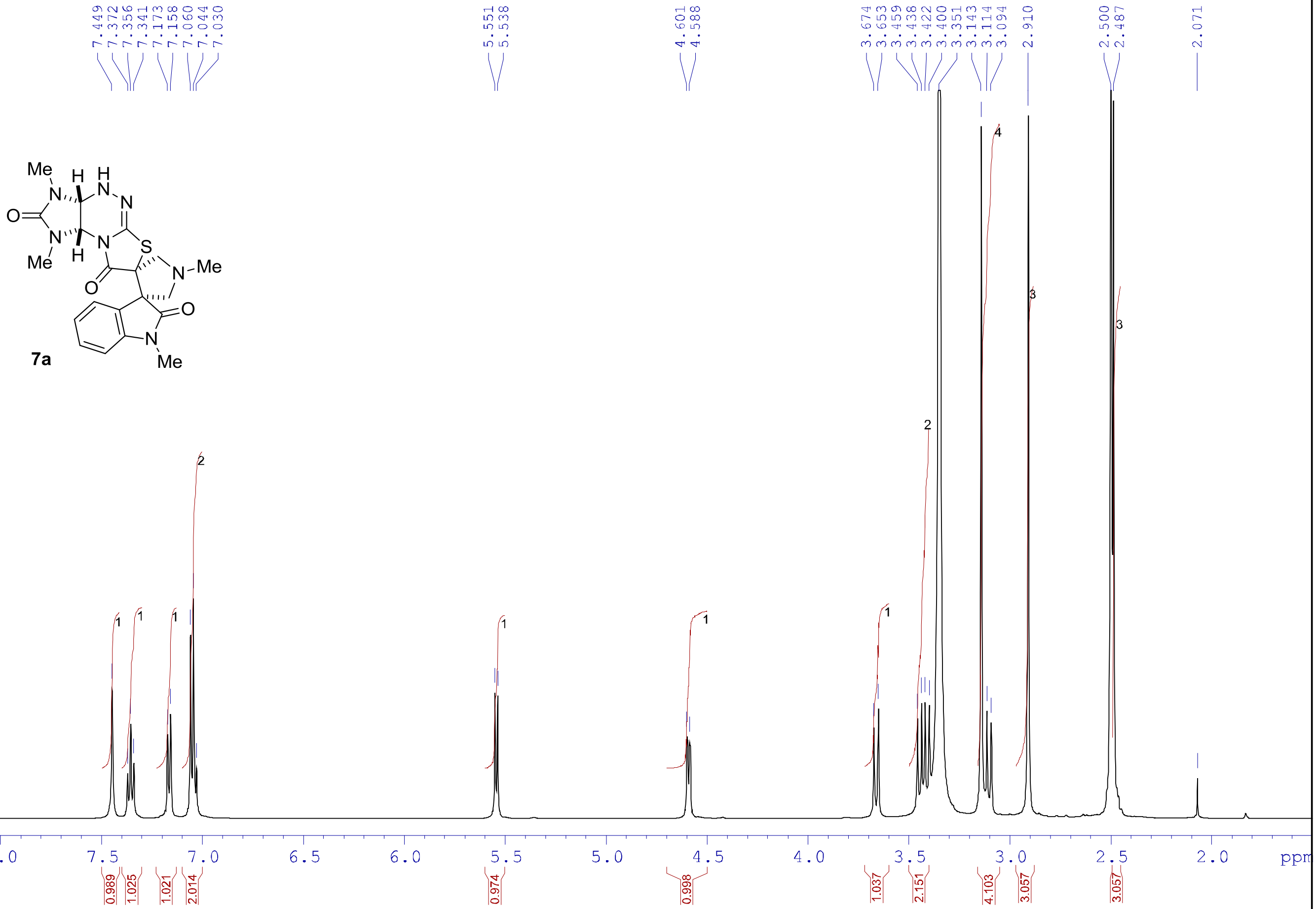

7a

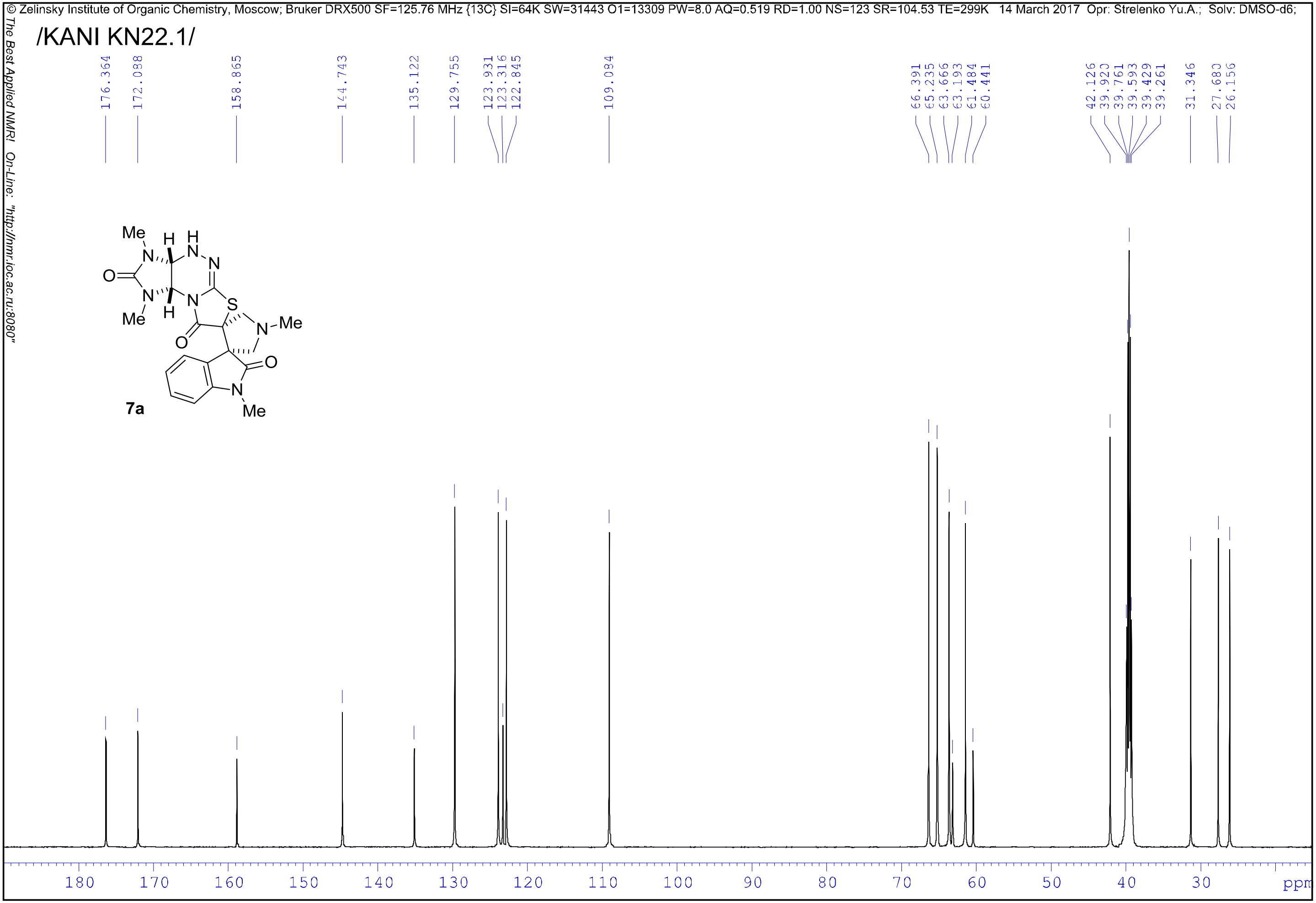
© Zelinsky Institute of Organic Chemistry, Moscow; Bruker DRX500 SF=125.76 MHz {13C} SI=64K SW=31443 O1=13309 PW=8.0 AQ=0.519 RD=1.00 NS=123 SR=104.53 TE=299K 14 March 2017 Opr: Strelenko Yu.A.; Solv: DMSO-d6;
/KANI KN22.1/
The Best Applied NMR! On-Line: "http://nmr.ioc.ac.ru:8080"
176.364
172.088
158.865
144.743
135.122
129.755
123.931
123.316
122.845
109.084
66.391
65.235
63.666
63.193
61.484
60.441
42.126
39.920
39.761
39.593
39.429
39.261
31.346
27.680
26.156
7a
180
170
160
150
140
130
120
110
100
90
80
70
60
50
40
30
ppm

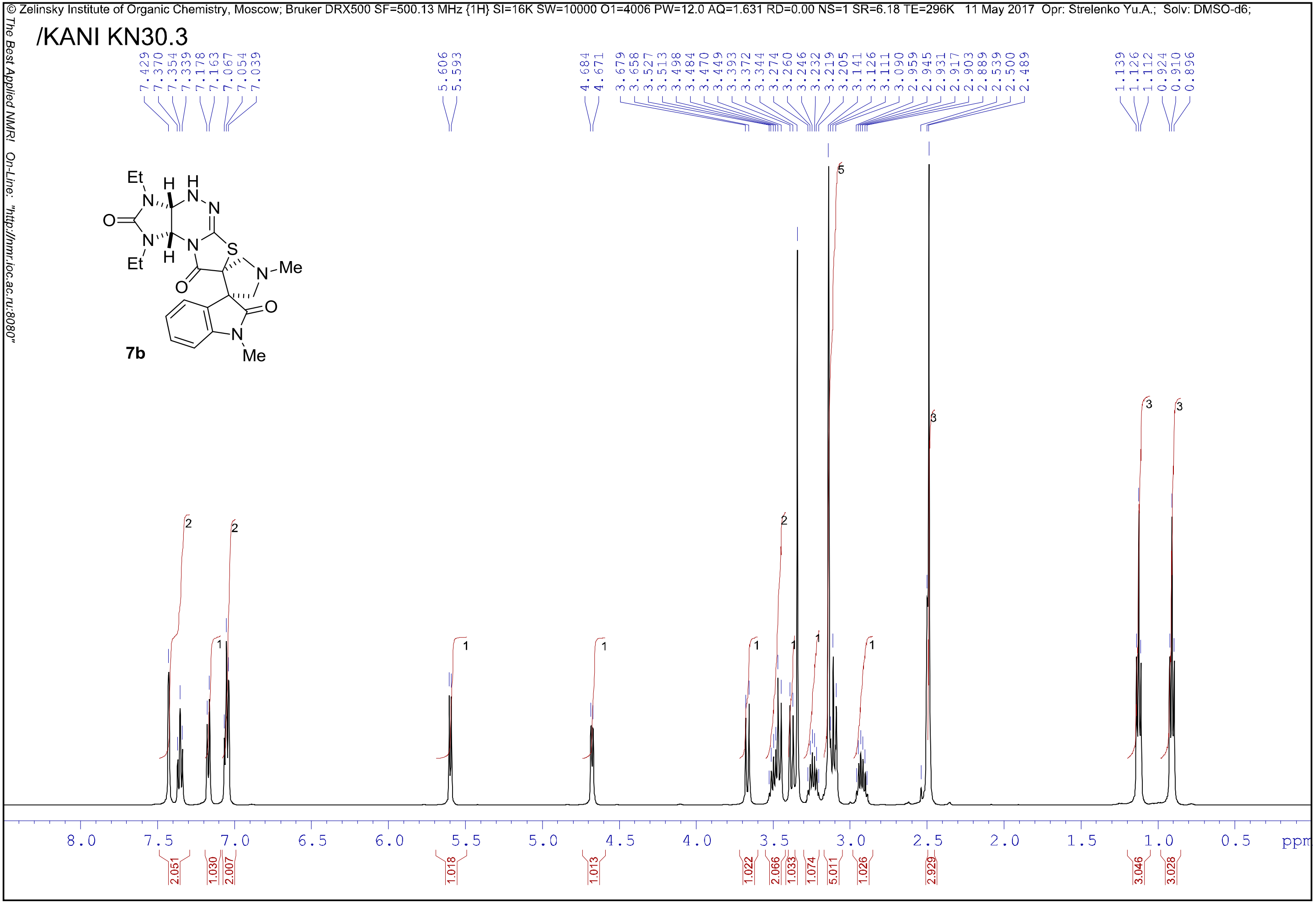
© Zelinsky Institute of Organic Chemistry, Moscow; Bruker DRX500 SF=500.13 MHz {1H} SI=16K SW=10000 O1=4006 PW=12.0 AQ=1.631 RD=0.00 NS=1 SR=6.18 TE=296K 11 May 2017 Opr: Strelenko Yu.A.; Solv: DMSO-d6;
The Best Applied NMR! On-Line: "http://nmr.ioc.ac.ru:8080"
/KANI KN30.3
7.429 7.370 7.354 7.339 7.178 7.163 7.067 7.054 7.039
5.606 5.593
4.684 4.671
3.679 3.658 3.527 3.513 3.498 3.484 3.470 3.449 3.393 3.372 3.344 3.274 3.260 3.246 3.232 3.219 3.205 3.141 3.126 3.111 3.090 2.959 2.945 2.931 2.917 2.903 2.889 2.539 2.500 2.489
1.139 1.126 1.112 0.924 0.910 0.896
Et
H
H
N
N
N
O
N
Et
H
N
S
N
Me
O
O
N
Me
7b
8.0 7.5 7.0 6.5 6.0 5.5 5.0 4.5 4.0 3.5 3.0 2.5 2.0 1.5 1.0 0.5 ppm
2.051 1.030 2.007 1.018 1.013 1.022 2.066 1.033 1.074 5.011 1.026 2.929 3.046 3.028

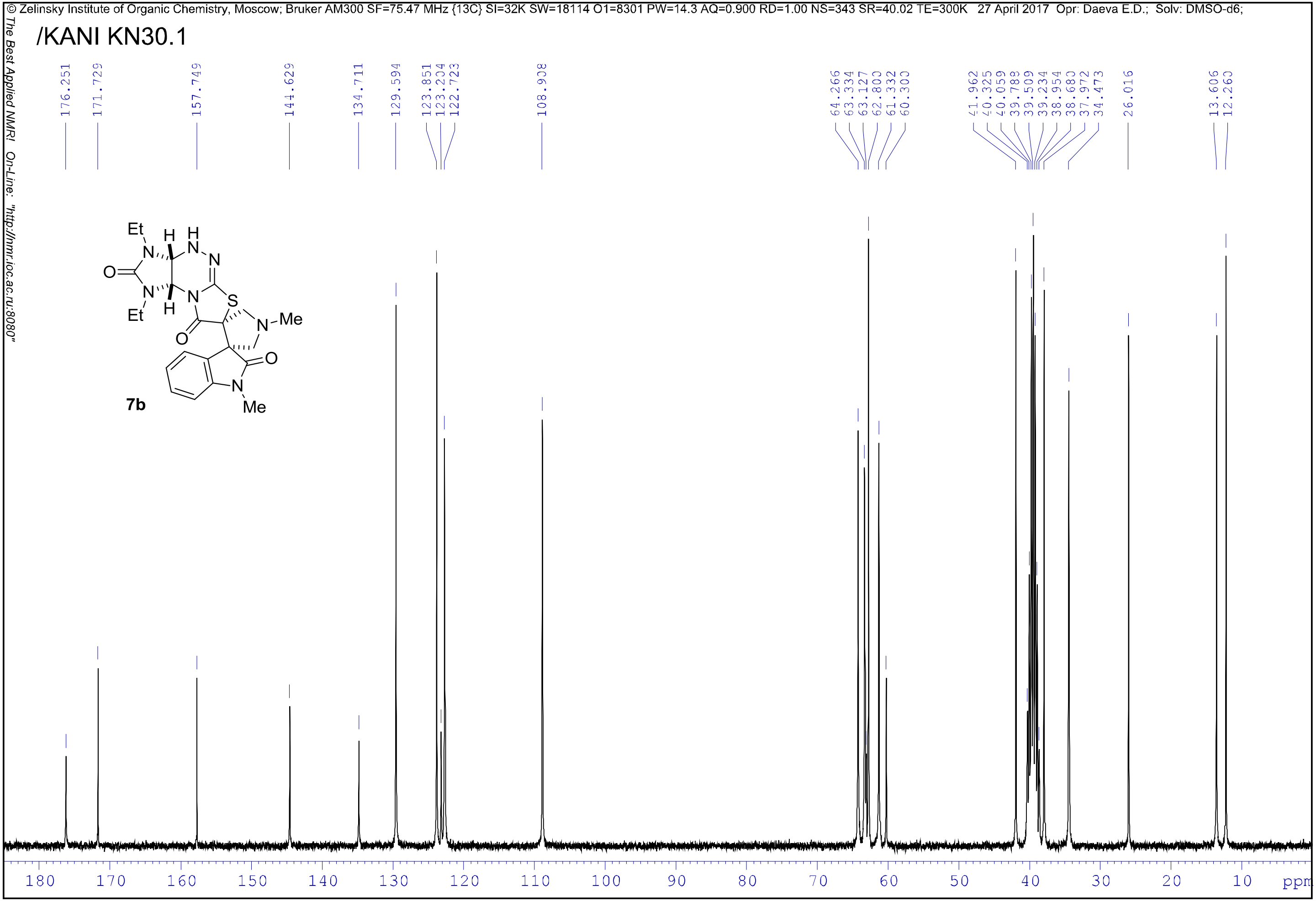
© Zelinsky Institute of Organic Chemistry, Moscow; Bruker AM300 SF=75.47 MHz {13C} SI=32K SW=18114 O1=8301 PW=14.3 AQ=0.900 RD=1.00 NS=343 SR=40.02 TE=300K 27 April 2017 Opr: Daeva E.D.; Solv: DMSO-d6;
The Best Applied NMR! On-Line: "http://nmr.ioc.ac.ru:8080"
/KANI KN30.1
176.251
171.729
157.749
144.629
134.711
129.594
123.851
123.234
122.723
108.908
64.266
63.334
63.127
62.800
61.332
60.300
41.962
40.325
40.059
39.783
39.509
39.234
38.954
38.680
37.972
34.473
26.016
13.606
12.263
7b
180 170 160 150 140 130 120 110 100 90 80 70 60 50 40 30 20 10 ppm

© Zelinsky Institute of Organic Chemistry, Moscow; Bruker DRX500 SF=500.13 MHz {1H-1H}NOESY SI=1024x256 SW=3931 O1=2065 PW=14.4 AQ=0.112 RD=1.00 NS=4x128 SR=6.18 TE=296K 11 May 2017 Opr: Strelenko Yu.A.; Solv: DMSO-d6;

The Best Applied NMR! On-Line: "http://nmr.ioc.ac.ru:8080"

/KANI KN30.3

NOESY

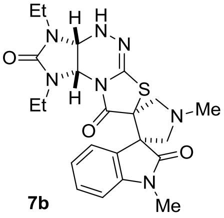

7b

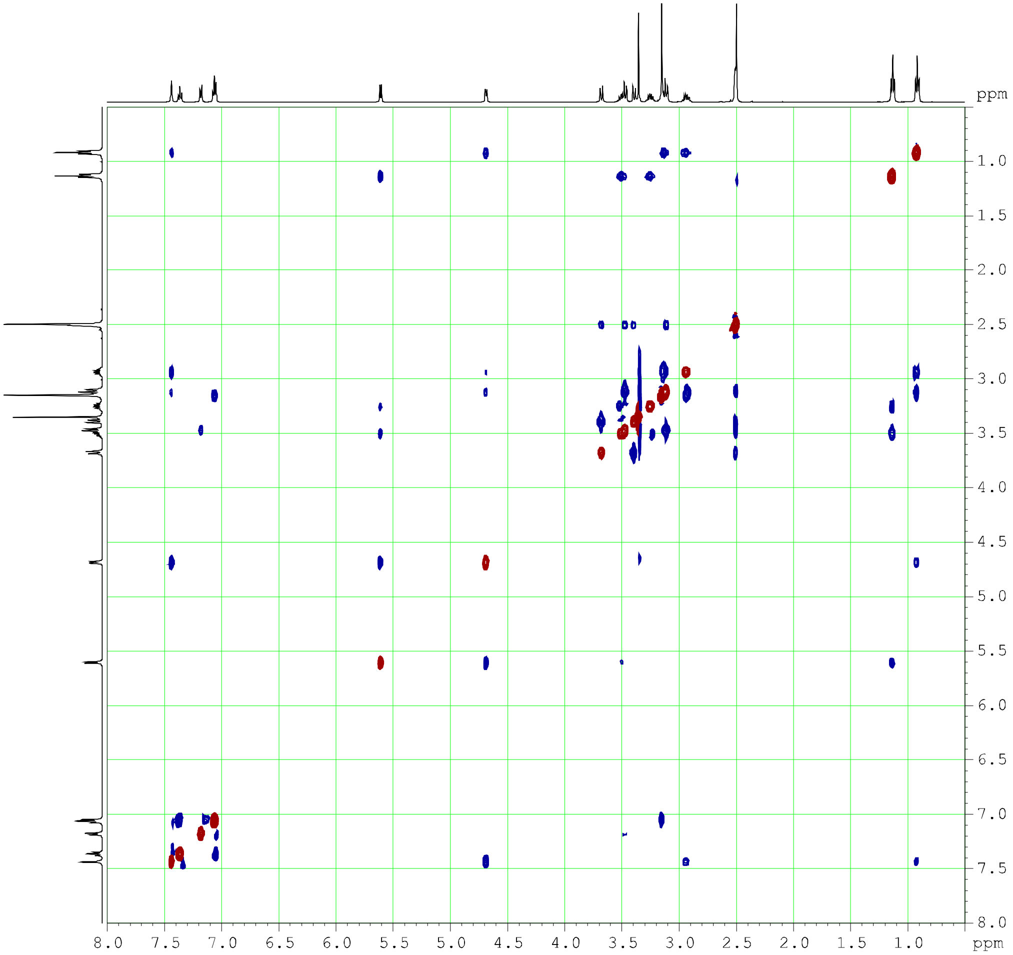

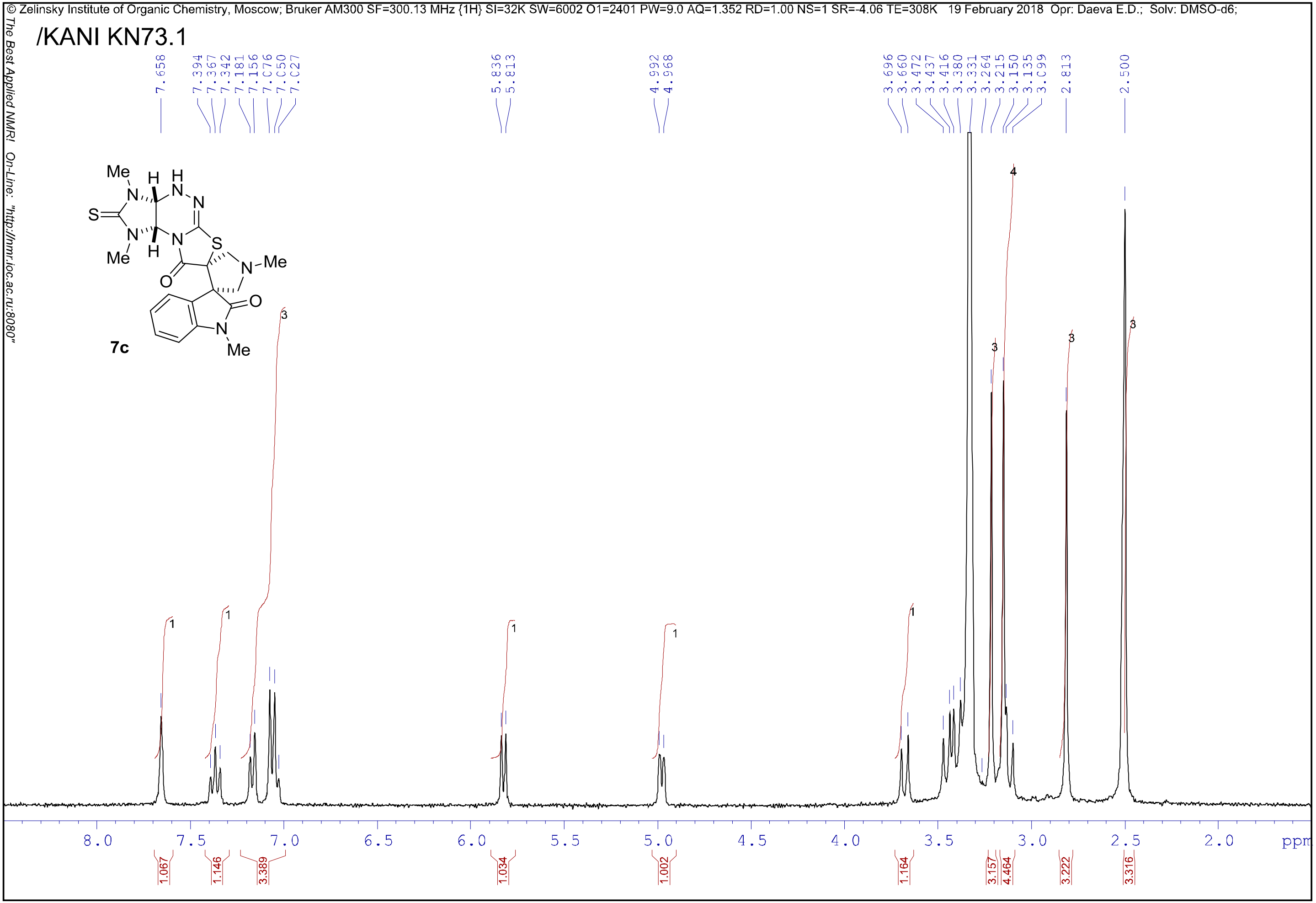
© Zelinsky Institute of Organic Chemistry, Moscow; Bruker AM300 SF=300.13 MHz {1H} SI=32K SW=6002 O1=2401 PW=9.0 AQ=1.352 RD=1.00 NS=1 SR=-4.06 TE=308K 19 February 2018 Opr: Daeva E.D.; Solv: DMSO-d6;
The Best Applied NMR! On-Line: "http://nmr.ioc.ac.ru:8080"
/KANI KN73.1
7.658 7.394 7.367 7.342 7.181 7.156 7.076 7.050 7.027
5.836 5.813
4.992 4.968
3.696 3.660 3.472 3.437 3.416 3.380 3.331 3.264 3.215 3.150 3.135 3.099 2.813 2.500
7c
1.067 1.146 3.389 1.034 1.002 1.164 3.157 4.464 3.222 3.316
ppm

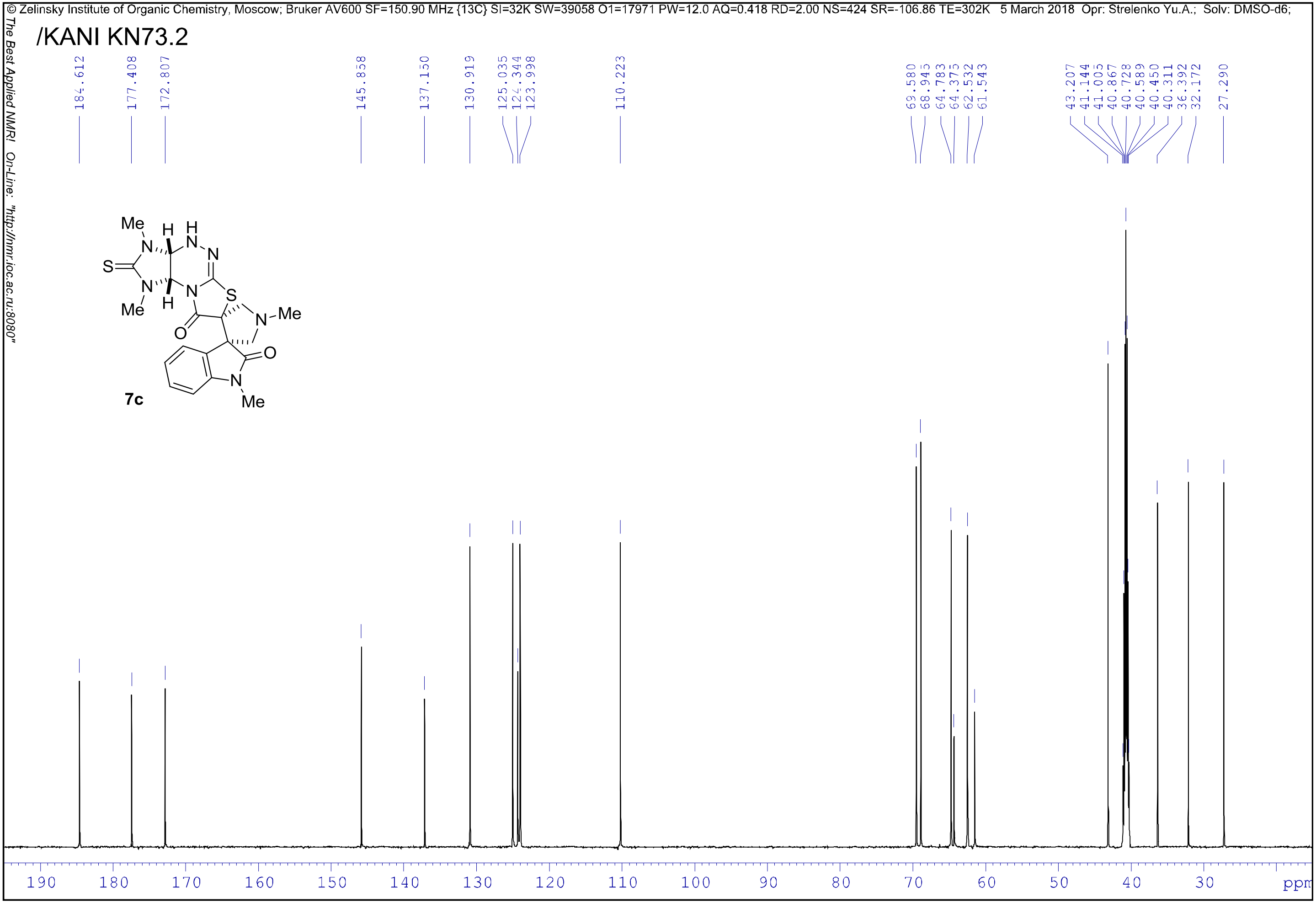
© Zelinsky Institute of Organic Chemistry, Moscow; Bruker AV600 SF=150.90 MHz {13C} SI=32K SW=39058 O1=17971 PW=12.0 AQ=0.418 RD=2.00 NS=424 SR=-106.86 TE=302K 5 March 2018 Opr: Strelenko Yu.A.; Solv: DMSO-d6;
/KANI KN73.2
The Best Applied NMR! On-Line: "http://nmr.ioc.ac.ru:8080"
184.612
177.408
172.807
145.858
137.150
130.919
125.035
124.344
123.998
110.223
69.580
68.945
64.783
64.375
62.532
61.543
43.207
41.144
41.005
40.867
40.728
40.589
40.450
40.311
36.392
32.172
27.290
7c
190 180 170 160 150 140 130 120 110 100 90 80 70 60 50 40 30 ppm

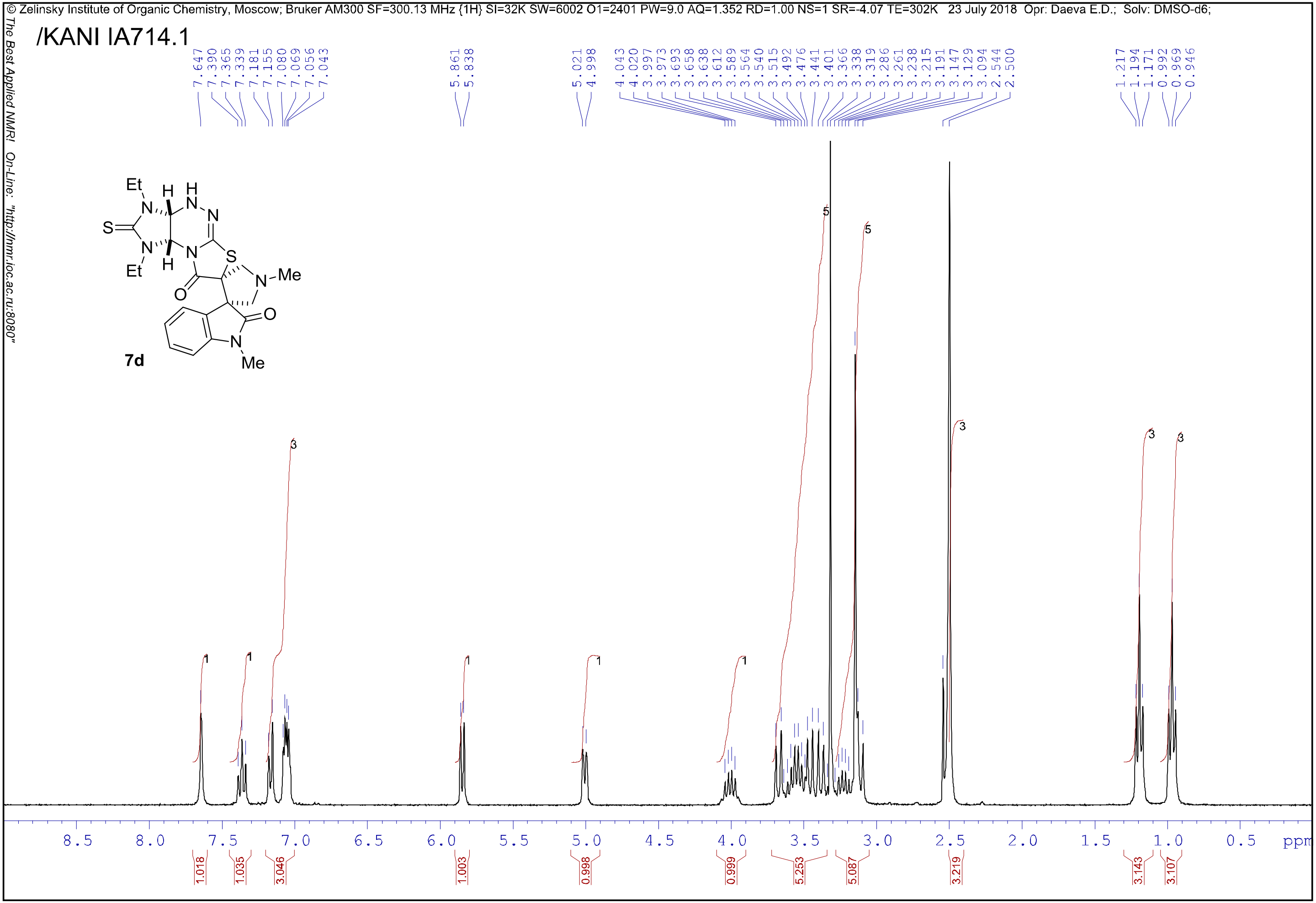
© Zelinsky Institute of Organic Chemistry, Moscow; Bruker AM300 SF=300.13 MHz {1H} SI=32K SW=6002 O1=2401 PW=9.0 AQ=1.352 RD=1.00 NS=1 SR=-4.07 TE=302K 23 July 2018 Opr: Daeva E.D.; Solv: DMSO-d6;
The Best Applied NMR! On-Line: "http://nmr.ioc.ac.ru:8080"
/KANI IA714.1
7.647 7.390 7.365 7.339 7.181 7.155 7.080 7.069 7.056 7.043
5.861 5.838
5.021 4.998
4.043 4.020 3.997 3.973 3.693 3.658 3.638 3.612 3.589 3.564 3.540 3.515 3.492 3.476 3.441 3.401 3.366 3.338 3.319 3.286 3.261 3.238 3.215 3.191 3.147 3.129 3.094 2.544 2.500
1.217 1.194 1.171 0.992 0.969 0.946
7d
8.5 8.0 7.5 7.0 6.5 6.0 5.5 5.0 4.5 4.0 3.5 3.0 2.5 2.0 1.5 1.0 0.5 ppm
1.018 1.035 3.046 1.003 0.998 0.999 5.253 5.087 3.219 3.143 3.107

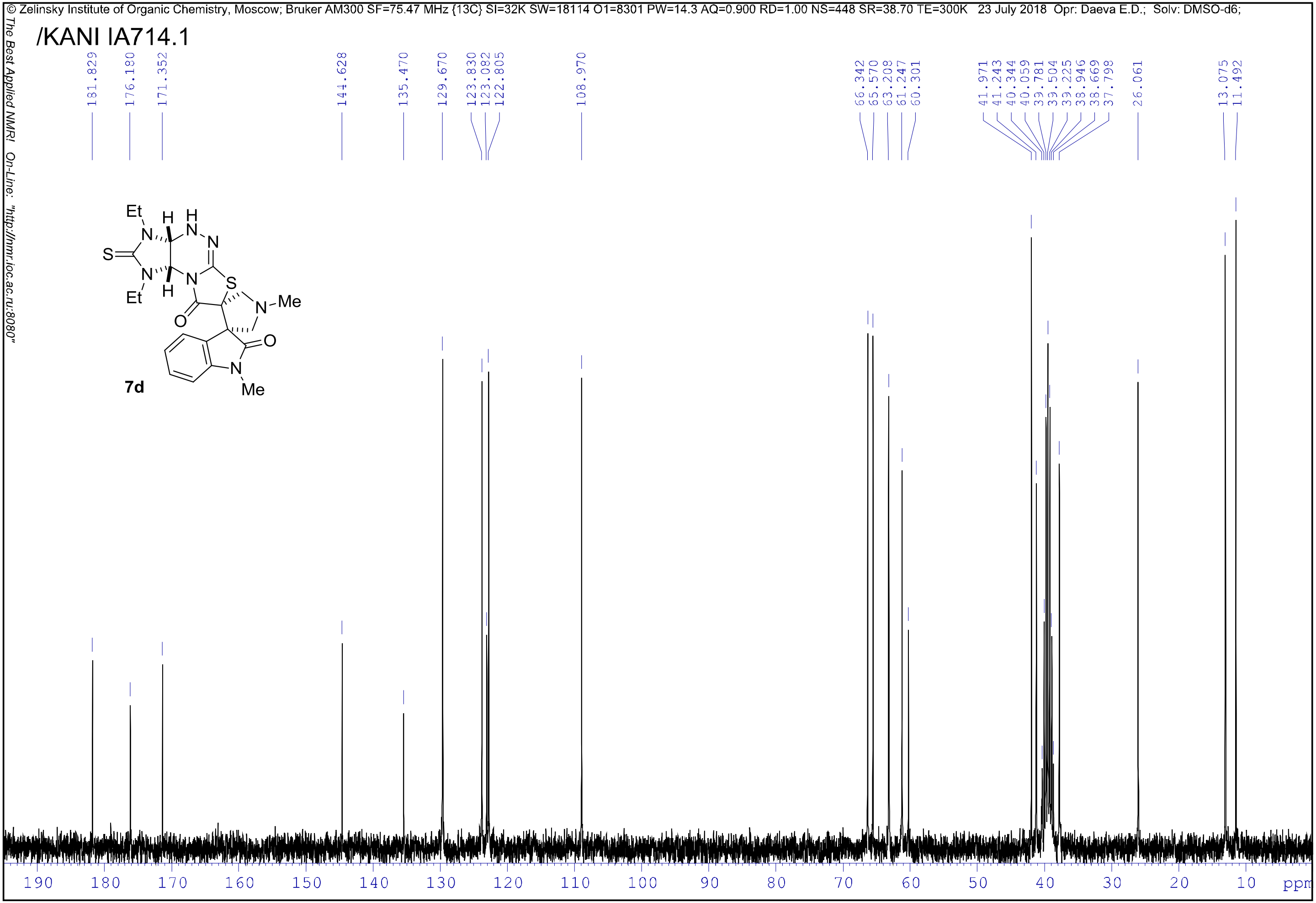
© Zelinsky Institute of Organic Chemistry, Moscow; Bruker AM300 SF=75.47 MHz {13C} SI=32K SW=18114 O1=8301 PW=14.3 AQ=0.900 RD=1.00 NS=448 SR=38.70 TE=300K 23 July 2018 Opr: Daeva E.D.; Solv: DMSO-d6;
The Best Applied NMR! On-Line: "http://nmr.ioc.ac.ru:8080"
/KANI IA714.1
181.829
176.180
171.352
144.628
135.470
129.670
123.830
123.082
122.805
108.970
66.342
65.570
63.208
61.247
60.301
41.971
41.243
40.344
40.059
39.781
39.504
39.225
38.946
38.669
37.798
26.061
13.075
11.492
7d
190 180 170 160 150 140 130 120 110 100 90 80 70 60 50 40 30 20 10 ppm

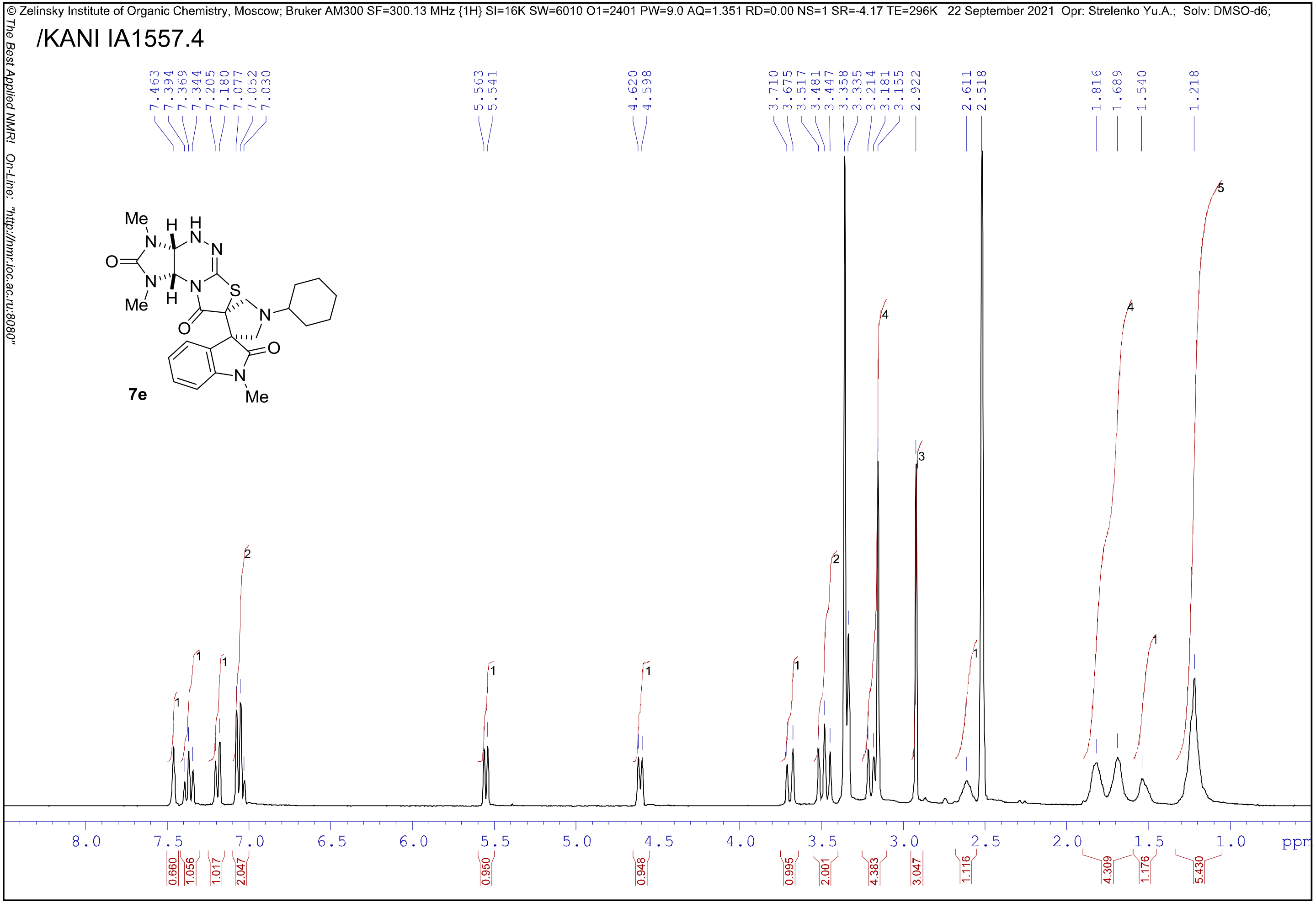
© Zelinsky Institute of Organic Chemistry, Moscow; Bruker AM300 SF=300.13 MHz {1H} SI=16K SW=6010 O1=2401 PW=9.0 AQ=1.351 RD=0.00 NS=1 SR=-4.17 TE=296K 22 September 2021 Opr: Strelenko Yu.A.; Solv: DMSO-d6;
The Best Applied NMR! On-Line: "http://nmr.ioc.ac.ru:8080"
/KANI IA1557.4
7.463 7.394 7.369 7.344 7.205 7.180 7.077 7.052 7.030
5.563 5.541
4.620 4.598
3.710 3.675 3.517 3.481 3.447 3.358 3.335 3.214 3.181 3.155 2.922
2.611 2.518
1.816 1.689 1.540
1.218
7e
ppm
0.660 1.056 1.017 2.047 0.950 0.948 0.995 2.001 4.383 3.047 1.116 4.309 1.176 5.430

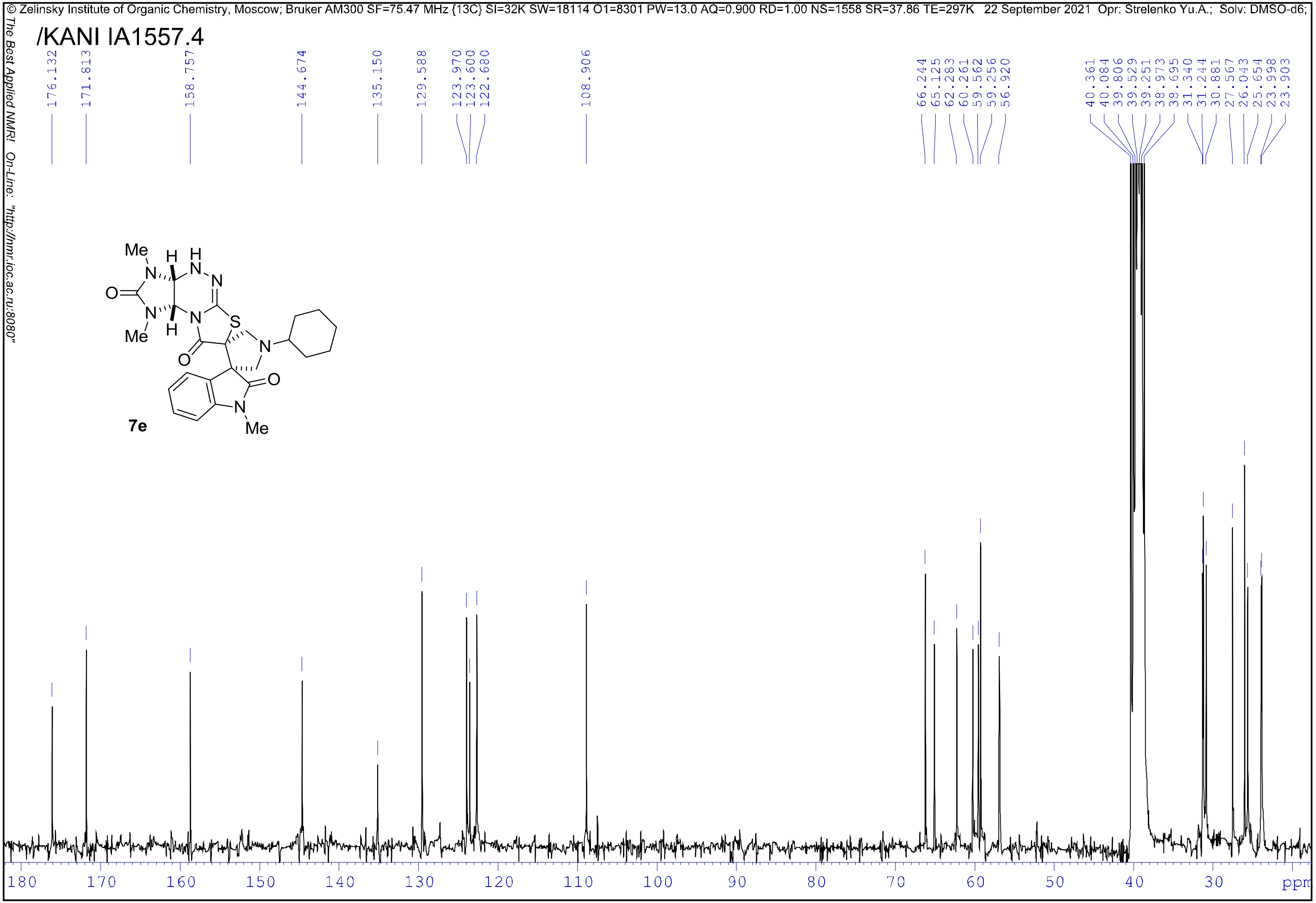
© Zelinsky Institute of Organic Chemistry, Moscow; Bruker AM300 SF=75.47 MHz {13C} SI=32K SW=18114 O1=8301 PW=13.0 AQ=0.900 RD=1.00 NS=1558 SR=37.86 TE=297K 22 September 2021 Opr: Strelenko Yu.A.; Solv: DMSO-d6;
The Best Applied NMR! On-Line: "http://nmr.ioc.ac.ru:8080"
/KANI IA1557.4
176.132
171.813
158.757
144.674
135.150
129.588
123.970
123.600
122.680
108.906
66.244
65.125
62.283
60.261
59.562
59.256
56.920
40.361
40.084
39.806
39.529
39.251
38.973
38.695
31.340
31.244
30.881
27.567
26.043
25.654
23.998
23.903
7e
180 170 160 150 140 130 120 110 100 90 80 70 60 50 40 30 ppm
